# Expedient synthesis of eumelanin-inspired 5,6-dihydroxyindole-2-carboxylate ethyl ester derivatives†

**DOI:** 10.1039/c8ra06148c

**Published:** 2018-08-07

**Authors:** Andrew H. Aebly, Jeffrey N. Levy, Benjamin J. Steger, Jonathan C. Quirke, Jason M. Belitsky

**Affiliations:** Department of Chemistry and Biochemistry, Oberlin College 119 Woodland St. Oberlin OH 44074 USA Jason.Belitsky@Oberlin.edu

## Abstract

Dihydroxyindoles such as 5,6-dihydroxyindole-2-carboxylic acid (DHICA) are the main monomer units of eumelanin, the black to brown pigment in humans, and have emerging biological roles beyond melanin. Elaboration of commercially available 5,6-dimethoxy-2-carboxylate ethyl ester provides ready access to DHICA-inspired small molecules, including 3-(hetero)aryl-indoles and 4,7-di-(hetero)aryl-indoles.

## Abstract 2

Two concise syntheses of novel aryl- and heteroaryl-substituted 5,6-dimethoxyindole-2-carboxylate ethyl esters utilizing regioselective halogenation/dehalogenation and Suzuki coupling are presented.

## Graphical abstract

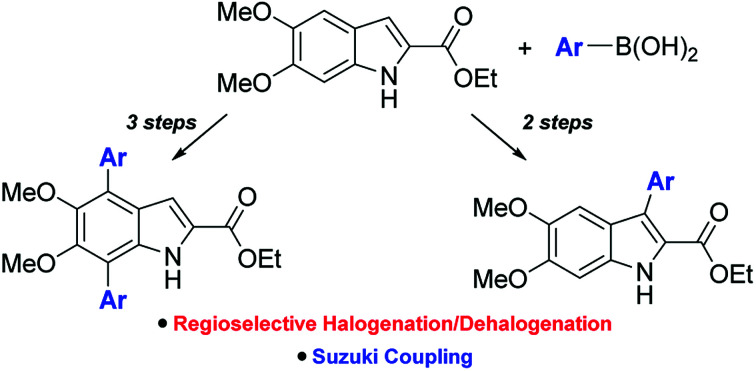

Eumelanin, the brown to black pigment found in the human body, has been the subject of investigation for nearly 100 years.^1^ This natural pigment, comprised of heterogeneous oligomers of 5,6-dihydroxyindoles that vary in both length and oxidation state, is formed through the oxidative polymerization of tyrosine *via* the catechol l-dopa.^2,3^ The most abundant monomers are 5,6-dihydroxyindole (DHI) and 5,6-dihydroxyindole-2-carboxylic acid (DHICA) (Fig. 1). The oxidative polymerization of dopamine to a DHI-containing material, polydopamine,^3,4^ has sparked an explosion of interest in the natural pigment and its analogs from the materials science community.^5^ Natural eumelanin, synthetic eumelanin derived from l-dopa, and polydopamine are being investigated for a wide variety of applications from batteries^6,7^ and organic electronics to water purification agents^8^ to bioadhesives and drug delivery systems.^3–5^ While the majority of work in this area has focused on the oxidative polymerization of l-dopa, dopamine, and other catechols,^7,9^ well-defined small molecule model systems^1,2,10–13^ can provide insight into these complex and heterogeneous polymeric systems.

**Fig. 1:**
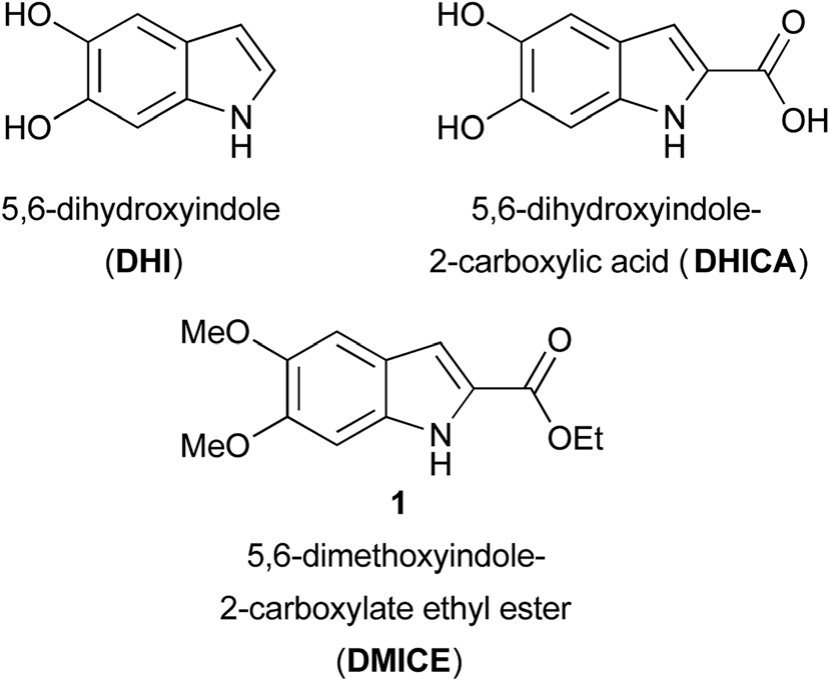
Eumelanin monomers DHI and DHICA and analog DMICE.

Small molecule models can also help elucidate the natural functions of free DHICA. It was long thought that free dihydroxyindoles were confined to the melanin-producing organelle known as the melanosome, but there is growing evidence that DHICA, in particular, has independent biological roles,^14^ including as a possible natural agonist of GPR35, an orphan G protein-coupled receptor (GPCR).^15^ A series of 3-aryl-DHICA derivatives 2 have been synthesized and tested as GPR35 agonists (Fig. 2a).^16^ The DHICA scaffold has also drawn interest for HIV-1 integrase inhibition, with bis- and hyperbranched DHICA esters showing potential as integrase inhibitors.^17,18^ Similar structures recently emerged as hits in a large scale virtual inhibition screening of influenza virus RNA-dependent RNA polymerase complex (RdRp).^19^ As DHICA itself is prone to auto-oxidation,^2^ we identified 5,6-dimethoxy-2-carboxylate ethyl ester 1 (DMICE) (Fig. 1) as a promising scaffold for the generation of DHICA-inspired small molecules.

**Fig. 2:**
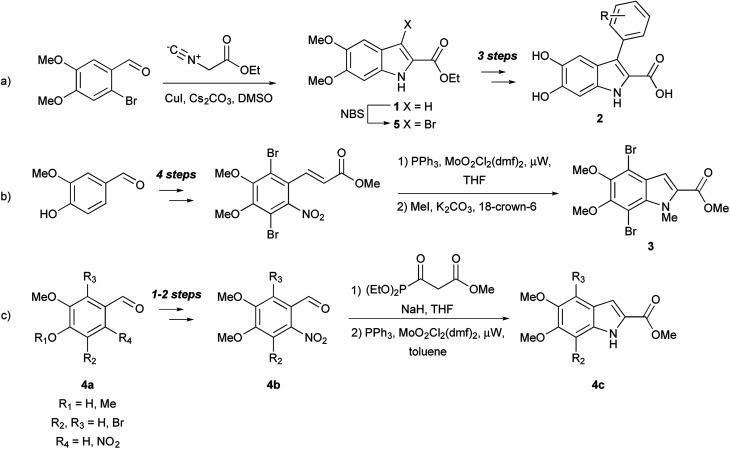
Previous synthetic approaches to DHICA/DMICE derivatives.

DHICA derivatives have been of interest to synthetic chemists since the 1960's, and have been produced *via* classical organic and biomimetic routes.^1^ Palladium-catalyzed chemistry, so widely used with other indoles,^20^ has been applied only sporadically toward dihydroxyindoles.^10−13,21^ En route to the formation of DHI oligomers, d'Ischia and co-workers have investigated Sonogashira couplings.^11,12^ The Nelson group has developed a six-step sequence from vanillin to create a 4,7-dibromo-DHICA scaffold 3 (Fig. 2b)^21^ and utilized a Sonogashira cross-coupling to elicit promising small molecule building blocks for organic semiconductors,^10^ and a polymerizable monomer.^21^ This work is of particular interest because DHICA is linked primarily through the 4,7-positions in natural eumelanin.^1,2^

Suzuki cross-coupling with alcohol- and nitrogen-protected DHICA derivatives was first reported in 2008 by Huleatt, Chai, and co-workers.^13^ These authors developed multiple synthetic schemes for the bromination of the 3-, 4- and 7-positions of the protected DHICA scaffold utilizing different starting materials.^22,23^ The synthetic route, from commercially available aldehydes (4a) *via* a Cadogan-type cyclization of intermediates (4b) to the indoles (4c), is shown in Fig. 2c. In order to generate potential GPR35 agonists, Deng and Fang used a strategy of halogenation and subsequent palladium-catalyzed coupling (Fig. 2a).^16^ DMICE (1), obtained from 2-bromo-4,5-dimethoxy-benzaldehyde, was regioselectively brominated to 3-bromo-DMICE (5). Suzuki cross-coupling of 5 with ten boronic acids, yielded eight 3-aryl derivatives, as well as was 3-pyridin-3-yl and 3-thiophen-3-yl derivatives. Removal of the ethyl and methyl groups generated the corresponding 3-aryl-DHICA analogs (2).

Now commercially available, 1 is an attractive scaffold for the generation of DHICA-inspired compounds. We explored the halogenation of DMICE and ultimately developed rapid routes to both 3- and 4,7-substituted aryl and heteroaryl derivatives without the need for protection of the indole nitrogen. Di-aryl/indole trimers and di-heteroaryl/indole trimers are accessible in three steps from DMICE with two purifications. The key advance toward these trimers was facile global bromination followed by regioselective debromination to selectively functionalize the eumelanin-relevant 4,7 positions. Suzuki coupling of the DMICE scaffold was optimized for 3- and 4,7-halogenated derivatives. Interestingly, 4,7-di-coupling proceeds more readily than coupling solely at the 3-position. The optimized mono- and di-Suzuki couplings proceeded in high yields with both electron-rich and electron-withdrawing substituents on the aryl boronic acid partners. We could thus generate a set of eumelanin-inspired model compounds with a wide range of substituents for future structure–activity relationship studies. The modularity, short linear sequences, and substrate tolerance of the synthetic approach suggest that it could ultimately serve as a platform for eumelanin oligomer synthesis. As a first step toward that goal and an exploration of DHICA-inspired compounds beyond the eumelanin context, here we describe the synthesis of twenty-six aryl- and heteroaryl DMICE derivatives, twenty-four of which have not been previously reported.

The functionalization of 1 began by exploring its reactivity with *N*-bromosuccinimide (NBS)^16^ under a variety of solvent and temperature conditions. Selective bromination at the 3-position occurs rapidly in acetonitrile at room temperature with a single equivalent of NBS to produce 5 (Scheme 1). With excess NBS at intermediate times and elevated temperature (2 h, 65 °C) 3,7-dibromo-DMICE is the major product, suggesting that the 7-position is more reactive than the 4-position (see ESI†). Triple bromination to 3,4,7-tribromo-DMICE (6) could be readily achieved with four equivalents of NBS at 65 °C for 24 h. The reactivity of the system is such that 4,7-dibromo-DMICE (7) is not a viable product directly from NBS halogenation. Nevertheless, 7 could be accessed in a two-step global halogenation/selective de-halogenation sequence, inspired by the work of Murakami *et al.*^24^ From 6, selective debromination at the 3-position is achieved by utilizing *m*-dimethoxybenzene under acidic conditions to trap the *in situ* generated bromine, giving 7 in a good yield over two steps (Scheme 1).^24^ The reactivity of the 3-position also allows for selective iodination with iodine under basic conditions to yield 3-iodo-DMICE (8).^25^

**Scheme 1:**
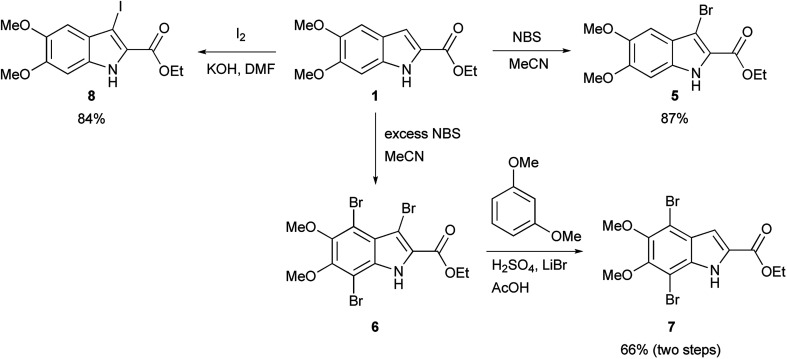
Halogenation of 1.

The Suzuki cross-coupling conditions were initially optimized with 5 and a substantial excess of 4-methoxyphenylboronic acid (Table 1, entry 1) utilizing XPhos Pd G2 as the catalyst in a THF/H_2_O mixture at 50 °C at 4% catalyst loading.^26^ A reduction in the equivalence of the boronic acid led to decreased conversion (entry 2). Switching the halogen from bromo-indole 5 to iodo-indole 8 (entry 3) and decreasing the amount of water in the reaction (entry 4) did not improve the conversion. Remaining with 8 and 1.25 equivalents of boronic acid, the reactivity was improved by switching to dioxane as the organic component of majority organic mixture and increasing the reaction temperature (entry 5). Further decreasing either the catalyst loading (entry 6) or the equivalence of the boronic acid (entry 7) resulted in incomplete conversion. The optimized conditions (Table 1, entry 5) improve upon the existing literature, reducing both the catalyst loading and the equivalence of the boronic acid coupling partner.^16^

**Table 1:** Optimization of Suzuki coupling of 5 and 8^a^

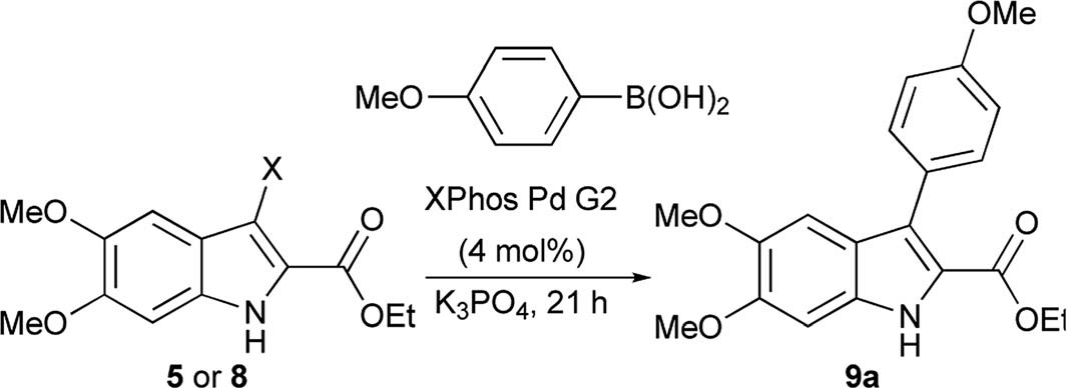					
Entry	X	Boronic acid (eq.)	Conditions	Conversion^b^	(Yield)^c^
1	Br	2.0	THF/H_2_O (1 : 1), 50 °C	100%	(94%)
2	Br	1.25	THF/H_2_O (1 : 1), 50 °C	70%	
3	I	1.25	THF/H_2_O (1 : 1), 50 °C	71%	
4	I	1.25	THF/H_2_O (11 : 1), 50 °C	70%	
5	I	1.25	Dioxane/H_2_O (11 : 1), 75 °C	100%	(99%)
6^d^	I	1.25	Dioxane/H_2_O (11 : 1), 75 °C	76%	
7	I	1.1	Dioxane/H_2_O (11 : 1), 75 °C	95%	(94%)

a Reaction conditions: 5 or 8 (0.04 mmol), 4-methoxyphenylboronic acid (0.044–0.080 mmol), XPhos Pd G2 (0.0016 mmol), K_3_PO_4_ (0.18 mmol), solvent (0.083 M) at 75 °C.

b Determined from crude ^1^H-NMR.

c Isolated yield.

d XPhos Pd G2 (1 mol%).

Having determined the appropriate conditions for the cross-coupling, a series of aryl boronic acids were examined to explore the scope of the reaction (Table 2). Yields were uniformly good for electron-rich and electron-poor boronic acids as well as the sterically hindered 1-naphthaleneboronic acid. A variety of heteroatom boronic acids also provided good yields including the unsubstituted indoles at both the 4- and 6-positions. However, some heteroaryl boronic acids were unsuccessful under these conditions. 4-Pyridineboronic acid and 4-pyrazoleboronic acid gave no desired product, presumably through deleterious coordination to the active Pd^0^ catalyst,^27^ while 2-furanboronic acid and 2-thiopheneboronic acid did not react, possibly as a result of competitive protodeborylation.^28^ Notably, 9e and 9j were synthesized in higher yields than the previously reported procedure,^16^ and the rest of the derivatives are novel compounds.

**Table 2:** Substrate scope of Suzuki coupling of 8^a^

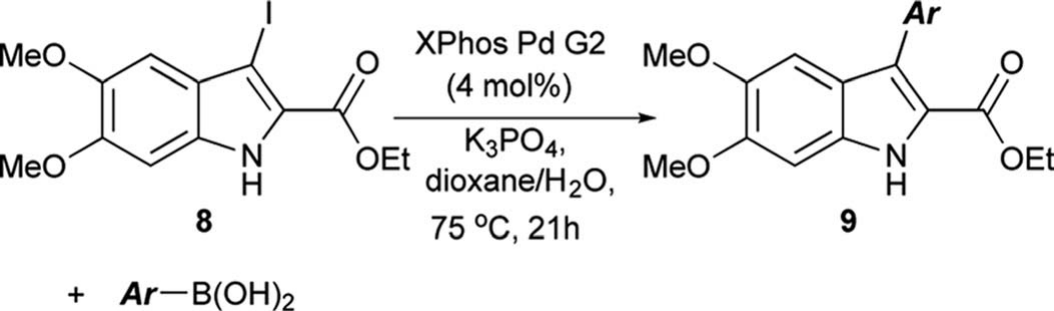
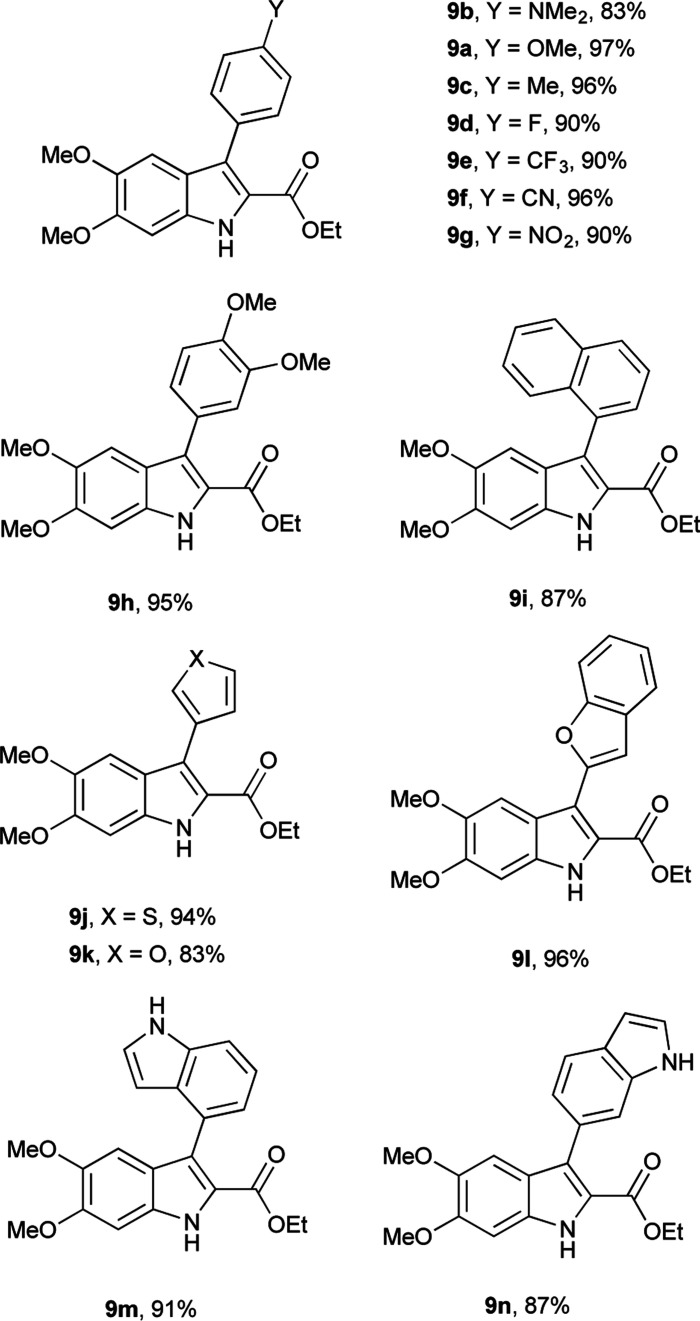

a Reaction conditions: 8 (0.06 mmol), boronic acid (0.075 mmol), XPhos Pd G2 (0.0024 mmol), K_3_PO_4_ (0.18 mmol), solvent (0.083 M) at 75 °C. Isolated yields shown.

Having shown the efficacy of the Suzuki cross-coupling on the simpler 3-I-DMICE (8), conditions for a double coupling of 4,7-diBr-DMICE (7) were explored. Upon monitoring the reaction with 4-methoxyphenyl boronic acid under the optimized conditions for the mono coupling, it became apparent that the substrate reacted much more rapidly in comparison to 8, providing a quantitative conversion in 3.5 hours (see ESI, Table S1†). Standard conditions^26^ established for the XPhos Pd G2 catalyst also supported a rapid reaction in excellent yield, particularly with 2.5 equivalents of the boronic acid partner. With the optimized conditions determined, a set of boronic acids was analyzed to explore the robustness of the reaction (Table 3). Again, both electron-rich and electron-poor substrates performed similarly, with the exception of 4-cyanophenylboronic acid which gave only a modest yield. Sulfur and oxygen-containing heterocycles also gave good to excellent yields. The indole trimer (10l) generated in three steps from commercially available DMICE 1 highlights the ability of this chemistry to generate model compounds with increasing relevance to eumelanin.

**Table 3:** Substrate scope of Suzuki coupling of 7^a^

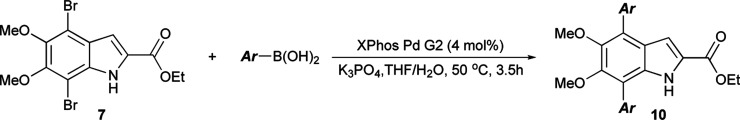
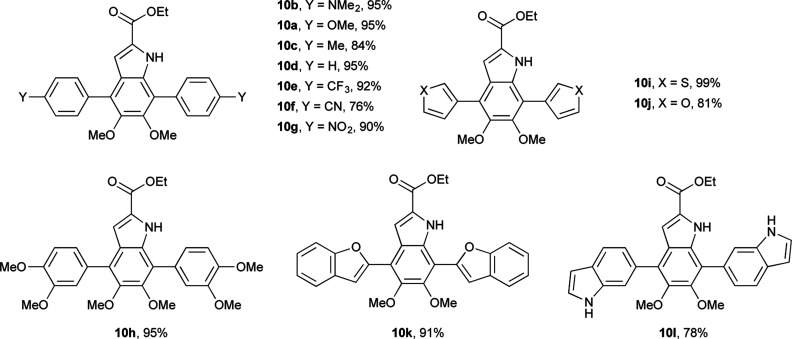

a Reaction conditions: 7 (0.04 mmol), boronic acid (0.096 mmol), XPhos Pd G2 (0.0016 mmol), 0.5 M K_3_PO_4_ (0.17 M), THF (0.17 M) at 50 °C. Isolated yields shown.

Having access to 26 DMICE derivatives with a range of aryl/heteroaryl substituents will allow us to systematically explore structure–activity relationships using these DHICA-inspired compounds. As a preliminary example, the unprotected indole–nitrogen in these compounds affords a proton in an uncluttered region of their ^1^H NMR spectra. Comparing the 3-aryl series, the chemical shift of this proton is clearly sensitive to remote substituents on the aryl ring in a Hammett fashion,^29^ varying from *δ* = 8.74 ppm for the dimethylamino substituent to *δ* = 9.05 ppm for the cyano substituent (see ESI, Table S2†). The 4,7-aryl series falls in much narrower range of *δ* = 8.64–8.73 ppm, perhaps indicating that steric interactions with the 7′-aryl ring are more significant than electronic differences of the remote aryl substituents. The 4,7-heteroaryl series have chemical shifts in a similar range, with the pronounced exception of 2-benzofuran at *δ* = 10.34 ppm, which is significantly deshielded compared to 3-furan (*δ* = 8.84 ppm) or 6-indole (*δ* = 8.72 ppm). This is potentially indicative of an intramolecular hydrogen bond with the indole NH as the donor and benzofuran O as the acceptor, although other factors such as ring current and anisotropy could also be responsible for the large chemical shift.^30^

In summation, regioselective halogenation and de-halogenation of commercially available DMICE (1) has been developed, providing access to two different DMICE scaffolds for derivatization. Exploration of additional substitution patterns (*i.e.*, 3,7-) is ongoing. Successful Suzuki cross-coupling conditions have been determined for 3-I-DMICE (8) and 4,7-diBr-DMICE (7) to produce 24 novel eumelanin-inspired compounds. Examination of the optical properties of these model compounds is underway. Also, this modular strategy is currently being explored for the synthesis of eumelanin dimers and trimers.

## Conflicts of interest

There are no conflicts of interest to declare.

## Supplementary Material

RA-008-C8RA06148C-s001
