# Correction: Expedient synthesis of eumelanin-inspired 5,6-dihydroxyindole-2-carboxylate ethyl ester derivatives

**DOI:** 10.1039/c9ra90065a

**Published:** 2019-09-03

**Authors:** Andrew H. Aebly, Jeffrey N. Levy, Benjamin J. Steger, Jonathan C. Quirke, Jason M. Belitsky

**Affiliations:** Department of Chemistry and Biochemistry, Oberlin College 119 Woodland St. Oberlin OH 44074 USA Jason.Belitsky@Oberlin.edu

## Abstract

Correction for ‘Expedient synthesis of eumelanin-inspired 5,6-dihydroxyindole-2-carboxylate ethyl ester derivatives’ by Andrew H. Aebly *et al.*, *RSC Adv.*, 2018, **8**, 28323–28328.

The authors regret that an incorrect grant number was shown in the acknowledgements section of the published article. The corrected section should read:

We thank the National Science Foundation (RUI grant CHE-1305919) and Oberlin College for financial support.

The Royal Society of Chemistry apologises for these errors and any consequent inconvenience to authors and readers.

## Supplementary Material

