# Supplementary material for: Expedient synthesis of eumelanin-inspired 5,6-dihydroxyindole-2-carboxylate ethyl ester derivatives
Source: RSC Adv. 2018 Aug 7;8(50):28323–8. doi: 10.1039/c8ra06148c (PMC9083952; doi:10.1039/c8ra06148c)
Supplement: RA-008-C8RA06148C-s001 [file RA-008-C8RA06148C-s001.pdf]

Electronic Supplementary Material (ESI) for RSC Advances.
This journal is © The Royal Society of Chemistry 2018

Supporting Information

Expedient Synthesis of Eumelanin-Inspired 5,6-Dihydroxyindole-2-Carboxylate Ethyl Ester Derivatives

Andrew H. Aebly, Jeffrey N. Levy, Benjamin J. Steger, Jonathan C. Quirke, and Jason M. Belitsky*

Oberlin College, Chemistry and Biochemistry Department, 119 Woodland Street, Oberlin, OH 44074, USA

E-mail: Jason.Belitsky@oberlin.edu

Table of Contents

General Information……………………………………………………………………….1

Tables………………………………………………………………………………………...1

Experimental Procedures…………………………………………………………………….2

References…………………………………………………………………………………..10

NMR Spectra……………………………………………………………………………….11

High Resolution Mass Spectra……………………………………………………………...73

General Information

DMICE was purchased from Alfa Aesar, BroadPharm, and Oxchem. All reagents, except for *DMICE and N-bromosuccinimide*, were used as received from the commercial vendor. *DMICE* was dissolved in a mixture of dichloromethane/THF and filtered through a short silica plug with EtOAc prior to use. *N*-bromosuccinimide was recrystallized from benzene. Solvents were purchased from Sigma-Aldrich in anhydrous form and used as received. Column chromatography was performed using silica gel (80-200 mesh, JT Baker). The ^{1}H and ^{13}C NMR spectra were obtained on a Varian 400 MHz instrument using either $CDCl_3$ or d_6-acetone (Cambridge Isotopes Laboratories), correcting the chemical shifts (reported in in ppm) to the corresponding residual deuterated solvent peak. The following abbreviations were used to identify the multiplicities in the NMR spectra: s (singlet), d (doublet), t (triplet), q (quartet), b (broad), and apt (apparent). High resolution mass spectra (HRMS) were obtained using an Agilent Q-TOF HPLC-MS (University of Michigan) or Kratos MS25 double focusing mass spectrometer (Case Western Reserve University).

Tables

Table S1 Optimization of Suzuki coupling for **7**

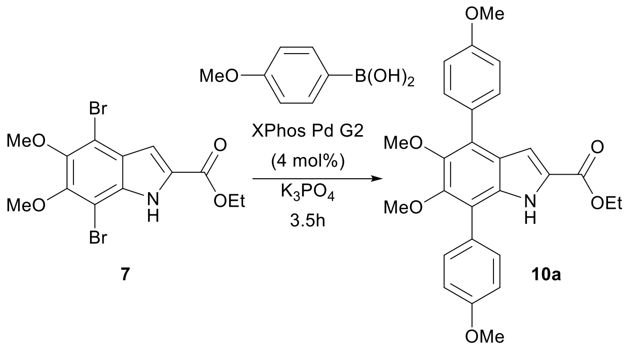

Entry	Conditions	Conversion^{b}	Yield^{c}
1	dioxane/H_2O (11:1), 75 °C	100%	82%
2	dioxane/H_2O (11:1), 50 °C	72%	71%
3	THF/H_2O (1:1), 50 °C	100%	97%
4^{d}	THF/H_2O (1:1), 50 °C	100%	89%

^{a}Reaction conditions: **7** (0.04 mmol),4-methoxyphenylboronic acid (0.096 mmol), XPhos Pd G2 (0.0016 mmol), K_3PO_4 (0.12 mmol), solvent (0.083 M), 3.5h.^{b}Determined from crude ^{1}H-NMR. ^{c}Isolated yield. ^{d}4-methoxyphenylboronic acid (0.084 mmol).

Table S2 ^{1}H-NMR indole N-H chemical shift

Substituent	3-Substituents Compound #	3-Substituents Chemical Shift	4,7-Substituents Compound #	4,7-Substituents Chemical Shift
4-Phenyl-NMe_2	**9b**	8.74	**10b**	8.71
4-Phenyl-OMe	**9a**	8.80	**10a**	8.64
4-Phenyl-Me	**9c**	8.81	**10c**	8.65
4-Phenyl-F	**9d**	8.83	--	--
Phenyl	--	--	**10d**	8.67
4-Phenyl-CF_3	**9e**	9.01	**10e**	8.64
4-Phenyl-CN	**9f**	9.05	**10f**	8.67
4-Phenyl-NO_2	**9g**	10.94*	**10g**	8.73
3,4-OMe	**9h**	8.79	**10h**	8.70
1-naphthyl	**9i**	9.09	--	--
3-thiophene	**9j**	8.82	**10i**	8.81
3-furan	**9k**	8.97	**10j**	8.84
2-benzofuran	**9l**	8.98	**10k**	10.34
4-indole	**9m**	8.98	--	--
6-indole	**9n**	8.84	**10l**	8.72

Spectra recorded in $CDCl_3$ except where noted. Chemical shift is in ppm.
*In acetone-d6

Experimental Procedures

Synthesis of **5**[1]

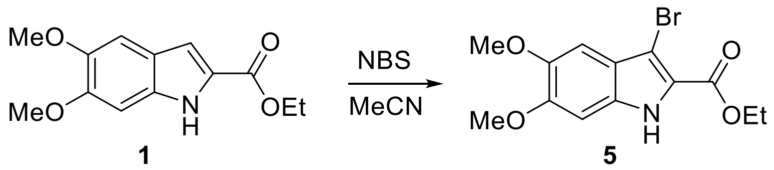

To a 50-mL round bottom flask added ethyl 5,6-dimethoxyindole-2-carboxylate **1** (1 equiv, 0.50 mmol) and partially dissolved in MeCN (2 mL). A solution of NBS (1.05 equiv, 0.525 mmol) in MeCN (7 mL) was added dropwise to the reaction flask over 5 min and allowed to stir at room temperature for an additional 30 min. The reaction was diluted with H_2O and filtered at 0 °C to produce **6**: 87% yield (off-white solid). **^{1}H NMR** (400 MHz, $CDCl_3$) δ 8.83 (s, 1H), 6.98 (s, 1H), 6.82 (s, 1H), 4.43 (q, J = 7.2 Hz, 2H), 3.97 (s, 3H), 3.94 (s, 3H), 1.44 (t, J = 7.1 Hz, 3H). **^{13}C NMR** (101 MHz, $CDCl_3$) δ 161.1, 151.2, 147.1, 130.5, 122.7, 121.4, 101.1, 98.1, 93.9, 61.2, 56.3, 56.2, 14.5.

Synthesis of **SI-1**

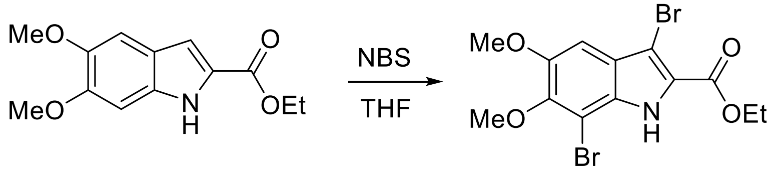

To a scintillation vial covered in tin foil added ethyl 5,6-dimethoxyindole-2-carboxylate **1** (1 equiv, 0.50 mmol) and NBS (4.0 equiv, 2.0 mmol). The starting materials were dissolved in THF (10 mL) and stirred at 65 °C for 2 h, then concentrated *in vacuo*. The crude product was dissolved in dichloromethane, washed with H_2O, dried with $MgSO_4$, concentrated and purified via column chromatography (silica, 8:1 hex/EtOAc) to produce **SI-1**: 69% yield (white solid). **^{1}H NMR** (400 MHz, $CDCl_3$) δ 8.90 (s, 1H), 7.01 (s, 1H), 4.46 (q, *J* = 7.0 Hz, 2H), 4.06 – 3.80 (m, 6H), 1.46 (t, *J* = 7.0 Hz, 3H). **^{13}C NMR** (101 MHz, $CDCl_3$) δ 160.5, 150.6, 147.8, 129.4, 124.7, 123.9, 101.4, 100.3, 98.4, 61.7, 61.4, 56.6, 14.5. **HRMS** (ESI) calculated for $C_{13}H_{14}NO_4Br_2$ ($[M+H]^+$): 405.9284; found: 405.9282; calculated ($[M+2+H]^+$): 407.9269; found: 407.9265; calculated ($[M+4+H]^+$): 409.9249; found: 409.9244.

Synthesis of **7**

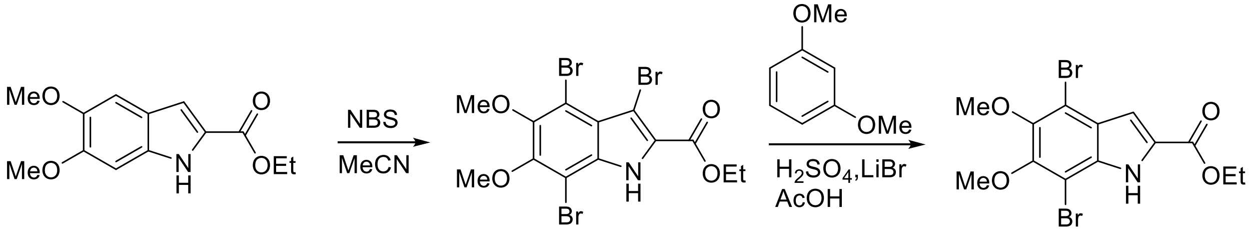

To a 20mL-scintillation vial covered in tin foil added ethyl 5,6-dimethoxyindole-2-carboxylate **1** (1 equiv, 0.80 mmol), N-bromosuccinimide (4 equiv, 3.20 mmol) and dissolved in acetonitrile (8 mL). The reaction was stirred at 65 °C for 24 h, concentrated *in vacuo* and filtered through a silica plug (3:1 to 2:1 hexanes:EtOAc) to yield **6**. The material was used in the following reaction without further purification. ^{1}H NMR (400 MHz, $CDCl_3$) δ 9.04 (s, 1H), 4.47 (q, *J* = 7.1 Hz, 2H), 3.97 (s, 3H), 3.90 (s, 3H), 1.46 (t, *J* = 7.1 Hz, 3H). ^{13}C NMR (101 MHz, $CDCl_3$) δ 160.3, 150.9, 147.9, 131.9, 126.3, 120.6, 110.4, 99.1, 98.7, 62.0, 61.7, 61.3, 14.5.

Adapted from Tani *et al.*[2] In a round-bottom flask **6** was dissolved in AcOH (0.07 M). To the reaction mixture LiBr (2.1 equiv, 0.6288 mmol), *m*-dimethoxybenzene (2.1 equiv, 0.6288 mmol) H_2SO_4 (2.1 equiv, 0.6288 mmol) was added and stirred at 65 °C for 1h. After cooling to room temperature, the product was precipitated out of solution with the addition of ice and filtered. The crude material was then purified via column chromatography (silica, hexanes: EtOAc: 8:1) to produce **7**: 66% yield, two steps (white solid). **^{1}H NMR** (400 MHz, $CDCl_3$) δ 8.96 (s, 1H), 7.26 (s, 1H), 4.43 (q, *J* = 7.1 Hz, 2H), 3.97 (s, 3H), 3.92 (s, 3H), 1.43 (t, *J* = 7.1 Hz, 3H). **^{13}C NMR** (101 MHz, $CDCl_3$) δ 161.3, 150.3, 147.1, 132.5, 128.7, 124.9, 110.0, 109.5, 99.0, 61.7, 61.6, 61.5, 14.5. **HRMS** (ESI) calculated for $C_{13}H_{14}NO_4Br_2$ ($[M+H]^+$): 405.9284; found: 405.9279; calculated ($[M+2+H]^+$): 407.9269; found: 407.9259; calculated ($[M+4+H]^+$): 409.9249; found: 409.9236.

Synthesis of **8**

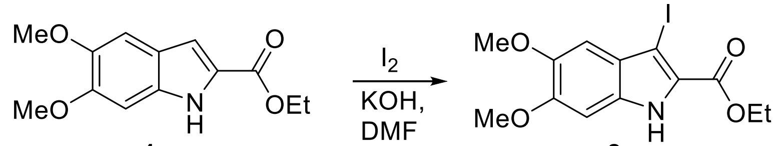

1 **8**

Adapted from Buchgraber *et al.*[3] To a 20mL-scintillation vial added ethyl 5,6-dimethoxyindole-2-carboxylate **1** (1 equiv, 0.80 mmol) and dissolved in DMF (0.8 mL). KOH (powdered, 5.03 eq, 4.024 mmol) was added to the reaction vial and stirred at 0^oC for 10 min. Over 5-10 minutes, 5 portions of I_2 (1.05 equiv, 0.84 mmol) were added to the reaction vial and continued stirring at 0^oC for an additional 30 min, then at r.t. for 45 min. The reaction was quenched by addition of sat NH_4Cl (8 mL) and sat $Na_2S_2O_3$ (8 mL). The precipitated solid was filtered, then re-dissolved in a mixture of ethyl acetate/dichloro-methane, dried with $MgSO_4$ and concentrated to produce **8**: 84% yield (off-white solid). **^{1}H NMR** (400 MHz, $CDCl_3$) δ 9.10 (s, 1H), 6.89 (s, 1H), 6.82 (s, 1H), 4.43 (q, *J* = 7.1 Hz, 2H), 3.98 (s, 3H), 3.94 (s, 3H), 1.45 (t, *J* = 7.1 Hz, 3H). **^{13}C NMR** (101 MHz, $CDCl_3$) δ 160.9, 151.2, 147.2, 131.0, 125.7, 125.0, 103.3, 93.7, 65.9, 61.3, 56.4, 56.3, 14.5. **HRMS** (ESI) calculated for $C_{13}H_{15}NO_4I$ ($[M+H]^+$): 376.0040, found: 376.0038.

General Procedure for Suzuki Coupling of 8

To a 1-dram reaction vial with a septum-cap added XPhosPd G2 (4 mol%, 0.0024 mmol), **8** (1 equiv, 0.06 mmol) and corresponding boronic acid (1.25 equiv, 0.075 mmol), evacuated with vacuum and purged with N_2. To the reaction vial added 1,4-dioxane (0.66 mL) and 0.5 M K_3PO_4 (3 equiv, 0.18 mmol). The reaction mixture was heated at 75 ^oC for 20 h and quenched with the addition of H_2O. The crude mixture was extracted with EtOAc (3 x 4 mL), dried with $MgSO_4$, filtered and concentrated *in vacuo*. The pure product was isolated via column chromatography (silica, hexanes: EtOAc: 3:2 to 2:1).

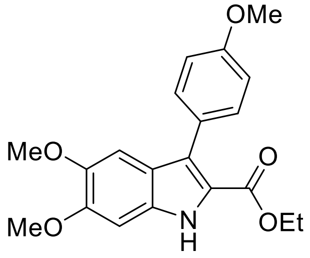

ethyl 5,6-dimethoxy-3-(4-methoxyphenyl)-1H-indole-2-carboxylate (**9a**): 97% yield (white solid). **^{1}H NMR** (400 MHz, $CDCl_3$) δ 8.80 (s, 1H), 7.49 (d, *J* = 8.6 Hz, 2H), 7.01 (d, *J* = 8.6 Hz, 2H), 6.97 (s, 1H), 6.86 (s, 1H), 4.27 (q, *J* = 7.1 Hz, 2H), 3.95 (s, 3H), 3.88 (s, 3H), 3.86 (s, 3H), 1.26 (d, *J* = 7.1 Hz, 3H). **^{13}C NMR** (101 MHz, $CDCl_3$) δ 162.0, 158.9, 150.5, 146.4, 131.7, 130.9, 126.2, 124.3, 121.3, 121.0, 113.5, 101.7, 93.7, 60.6, 56.3, 56.2, 55.4, 14.4. **HRMS** (ESI) calculated for $C_{20}H_{21}NO_5$ ($[M]^+$): 355.1420; found: 355.1426.

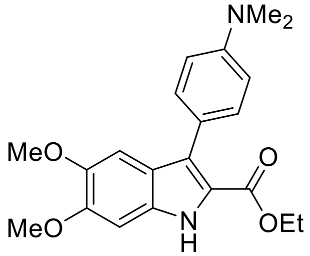

ethyl 3-(4-(dimethylamino)phenyl)-5,6-dimethoxy-1H-indole-2-carboxylate (**9b**): 83% yield (off-white solid). **^{1}H NMR** (400 MHz, $CDCl_3$) δ 8.74 (s, 1H), 7.48 (d, *J* = 8.7 Hz, 2H), 7.05 (s, 1H), 6.89 – 6.80 (m, 3H), 4.29 (q, *J* = 7.1 Hz, 2H), 3.95 (s, 3H), 3.86 (s, 3H), 3.03 (s, 6H), 1.28 (t, *J* = 7.1 Hz, 3H). **^{13}C NMR** (101 MHz, $CDCl_3$) δ 162.1, 150.4, 149.7, 146.2, 131.4, 131.1, 125.0, 121.0, 121.0, 112.1, 102.1, 93.6, 60.5, 56.3, 56.2, 40.8, 14.4. **HRMS** (ESI) calculated for $C_{21}H_{25}N_2O_4$ ($[M+H]^+$): 369.1809, found: 369.1819.

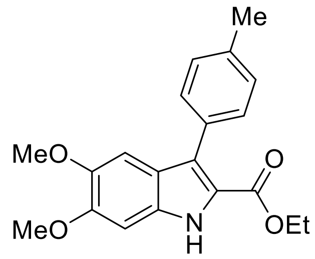

ethyl 5,6-dimethoxy-3-(p-tolyl)-1H-indole-2-carboxylate (**9c**): 96% yield (white solid). **^{1}H NMR** (400 MHz, $CDCl_3$) δ ^{1}H NMR (400 MHz, $CDCl_3$) δ 8.81 (s, 1H), 7.45 (d, *J* = 7.8 Hz, 2H), 7.27 (d, *J* = 7.8 Hz, 2H), 6.98 (s, 1H), 6.86 (s, 1H), 4.27 (q, *J* = 7.2 Hz, 2H), 3.95 (s, 3H), 3.85 (s, 3H), 2.43 (s, 3H), 1.25 (t, *J* = 7.1 Hz, 3H). **^{13}C NMR** (101 MHz, $CDCl_3$) δ 161.9, 150.5, 146.4, 136.9, 130.9, 130.9, 130.5, 128.7, 124.6, 121.4, 121.0, 101.8, 93.7, 60.6, 56.3, 56.2, 21.5, 14.3. **HRMS** (ESI) calculated for $C_{20}H_{22}NO_4$ ($[M+H]^+$): 340.1543, found: 340.1543.

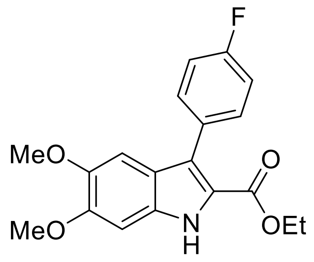

ethyl 3-(4-fluorophenyl)-5,6-dimethoxy-1H-indole-2-carboxylate (**9d**): 90% yield (white solid). **^{1}H NMR** (400 MHz, $CDCl_3$) δ 8.83 (s, 1H), 7.57 – 7.47 (m, 2H), 7.15 (appt t, *J* = 8.6 Hz, 2H), 6.90 (s, 1H), 6.87 (s, 1H), 4.27 (q, *J* = 7.1 Hz, 2H), 3.96 (s, 3H), 3.86 (s, 3H), 1.23 (t, *J* = 7.1 Hz, 3H). **^{13}C NMR** (101 MHz, $CDCl_3$) δ 162.2 (d, *J* = 244.4 Hz), 161.8, 150.6, 146.6, 132.2 (d, *J* = 8.1 Hz), 130.9, 129.9 (d, *J* = 3.4 Hz), 123.4, 121.6, 120.9, 114.9 (d, *J* = 21.6 Hz), 101.4, 93.7, 60.8, 56.3, 56.2, 14.3. **HRMS** (ESI) calculated for $C_{19}H_{19}NO_4F$ ($[M+H]^+$): 344.1293, found: 344.1294.

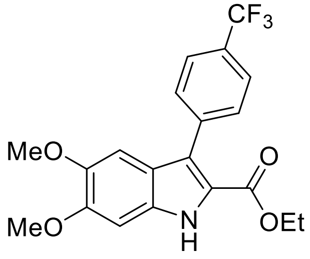

ethyl 5,6-dimethoxy-3-(4-(trifluoromethyl)phenyl)-1H-indole-2-carboxylate (**9e**): 90% (white solid). **^{1}H NMR** (400 MHz, $CDCl_3$) δ ^{1}H NMR) δ 9.01 (s, 1H), 7.72 (d, *J* = 8.2 Hz, 2H), 7.67 (d, *J* = 8.2 Hz, 2H), 6.90 (s, 1H), 6.89 (s, 1H), 4.28 (q, *J* = 7.1 Hz, 2H), 3.96 (s, 3H), 3.86 (s, 3H), 1.22 (t, *J* = 7.1 Hz, 3H). **^{13}C NMR** (101 MHz, $CDCl_3$) δ 161.7, 150.8, 146.9, 138.0, 130.9, 130.9 (2C), 129.2 ($^{2}J_{C\text{-}F}$ = 32 Hz), 124.9 ($^{3}J_{C\text{-}F}$ = 4 Hz), 124.5 ($^{1}J_{C\text{-}F}$ = 272 Hz), 122.8, 121.7, 120.7, 101.1, 93.8, 60.9, 56.3, 56.2, 14.24. **HRMS** (ESI) calculated for $C_{20}H_{19}NO_4F_3$ ($[M+H]^+$): 394.1261, found: 394.1265.

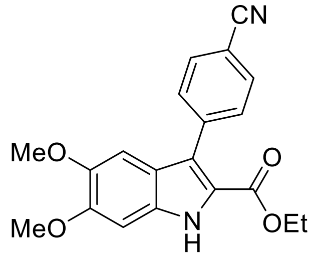

ethyl 3-(4-cyanophenyl)-5,6-dimethoxy-1H-indole-2-carboxylate (**9f**): 96% yield (white solid). **^{1}H NMR** (400 MHz, $CDCl_3$) δ) δ 9.05 (s, 1H), 7.75 (d, *J* = 8.1 Hz, 2H), 7.66 (d, *J* = 8.1 Hz, 2H), 6.89 (s, 1H), 6.87 (s, 1H), 4.28 (q, *J* = 7.1 Hz, 2H), 3.95 (s, 3H), 3.86 (s, 3H), 1.24 (t, *J* = 7.2 Hz, 3H). **^{13}C NMR** (101 MHz, $CDCl_3$) δ 161.4, 150.8, 147.1, 139.3, 131.8, 131.4, 130.9, 122.2, 121.7, 120.4, 119.3, 110.8, 100.8, 93.8, 61.0, 56.4, 56.3, 14.3. **HRMS** (ESI) calculated for $C_{20}H_{19}N_2O_4$ ($[M+H]^+$): 351.1339, found: 351.1342.

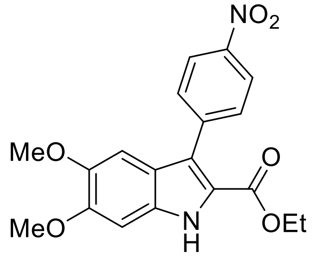

ethyl 5,6-dimethoxy-3-(4-nitrophenyl)-1H-indole-2-carboxylate (**9g**): 90% yield (yellow solid). **^{1}H NMR** (400 MHz, Acetone-d_6) δ 10.94 (s, 1H), 8.32 (d, *J* = 8.8 Hz, 2H), 7.87 (d, *J* = 8.8 Hz, 2H), 7.10 (s, 1H), 7.03 (s, 1H), 4.25 (q, *J* = 7.1 Hz, 2H), 3.88 (s, 3H), 3.78 (s, 3H), 1.22 (t, *J* = 7.1 Hz, 3H). ^{13}C NMR (101 MHz, Acetone-d_6) δ 161.8, 152.0, 148.4, 147.6, 142.9, 132.7, 132.6, 123.8, 123.0, 121.8, 121.0, 101.9, 95.5, 61.2, 56.5, 56.2, 14.6 **HRMS** (ESI) calculated for $C_{19}H_{19}N_2O_6$ ($[M+H]^+$): 371.1238; found: 371.1237.

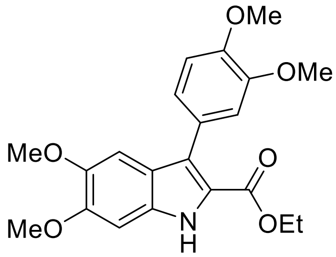

ethyl 3-(3,4-dimethoxyphenyl)-5,6-dimethoxy-1H-indole-2-carboxylate (**9h**): 95% yield (white solid). **^{1}H NMR** (400 MHz, $CDCl_3$) δ 8.79 (s, 1H), 7.16 – 7.09 (m, 2H), 7.01 – 6.96 (m, 2H), 6.87 (s, 1H), 4.28 (q, *J* = 7.1 Hz, 2H), 3.96 (s, 6H), 3.91 (s, 3H), 3.86 (s, 3H), 1.26 (t, *J* = 7.1 Hz, 3H). **^{13}C NMR** (101 MHz, $CDCl_3$) δ 161.9, 150.6, 148.4, 148.4, 146.5, 130.9, 126.5, 124.4, 123.0, 121.4, 121.1, 114.1, 110.9, 101.8, 93.7, 60.7, 56.3, 56.3, 56.1, 14.5. **HRMS** (ESI) calculated for $C_{21}H_{24}NO_6$ ($[M+H]^+$): 386.1598, found: 386.1601.

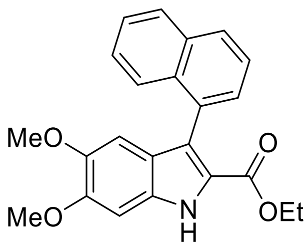

ethyl 5,6-dimethoxy-3-(naphthalen-1-yl)-1H-indole-2-carboxylate (**9i**): 87% yield (white solid). **^{1}H NMR** (400 MHz, $CDCl_3$) δ 9.09 (s, 1H), 7.92 (d, *J* = 8.2 Hz, 2H), 7.68 (d, *J* = 8.5 Hz, 1H), 7.60 – 7.51 (m, 2H), 7.47 (ddd, *J* = 8.1, 6.7, 1.2 Hz, 1H), 7.34 (ddd, *J* = 8.3, 6.7, 1.3 Hz, 1H), 6.94 (s, 1H), 6.65 (s, 1H), 4.04 – 3.95 (m, 5H), 3.71 (s, 3H), 0.73 (t, *J* = 7.1 Hz, 3H). **^{13}C NMR** (101 MHz, $CDCl_3$) δ 162.1, 150.6, 146.5, 133.7, 133.1, 132.3, 131.0, 128.4, 128.2, 127.8, 126.6, 125.8, 125.7, 125.3, 123.3, 122.1, 122.1, 101.9, 93.8, 60.4, 56.3, 56.3, 13.6. **HRMS** (ESI) calculated for $C_{23}H_{22}NO_4$ ($[M+H]^+$): 376.1543; found: 376.1545.

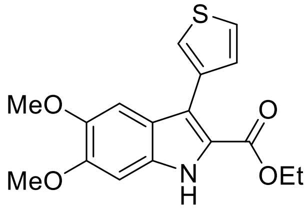

ethyl 5,6-dimethoxy-3-(thiophen-3-yl)-1H-indole-2-carboxylate (**9j**): 94% yield (white solid). **^{1}H NMR** (400 MHz, $CDCl_3$) δ 8.82 (s, 1H), 7.54 – 7.48 (m, 1H), 7.44 – 7.35 (m, 2H), 7.07 (s, 1H), 6.86 (s, 1H), 4.31 (q, *J* = 7.1 Hz, 2H), 3.95 (s, 3H), 3.89 (s, 3H), 1.30 (t, *J* = 7.1 Hz, 3H). **^{13}C NMR** (101 MHz, $CDCl_3$) δ 162.2, 150.9, 146.9, 134.1, 131.3, 130.5, 124.6, 124.4, 121.9, 121.3, 119.4, 102.2, 94.0, 61.1, 56.7, 56.5, 14.7. **HRMS** (ESI) calculated for $C_{17}H_{18}NO_4S$ ($[M+H]^+$): 332.0951, found: 332.0948.

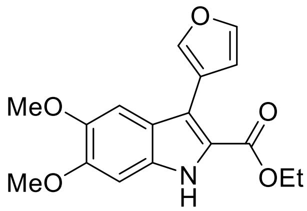

ethyl 3-(furan-3-yl)-5,6-dimethoxy-1H-indole-2-carboxylate (**9k**): 83% yield (white solid). **^{1}H NMR** (400 MHz, $CDCl_3$) δ 8.97 (s, 1H), 7.71 (s, 1H), 7.65 (d, *J* = 7.6 Hz, 1H), 7.57 (d, *J* = 8.0 Hz, 1H), 7.55 (s, 1H), 7.34 – 7.24 (m, 2H), 6.87 (s, 1H), 4.43 (q, *J* = 7.1 Hz, 2H), 3.99 (s, 3H), 3.97 (s, 3H), 1.41 (t, *J* = 7.1 Hz, 3H). **^{13}C NMR** (101 MHz, $CDCl_3$) δ 161.7, 150.6, 146.5, 142.3, 141.6, 131.0, 121.6, 120.7, 117.7, 114.9, 112.7, 101.8, 93.7, 60.8, 56.4, 56.2, 14.5. **HRMS** (ESI) calculated for $C_{17}H_{18}NO_5$ ($[M+H]^+$): 316.1178, found: 316.1179.

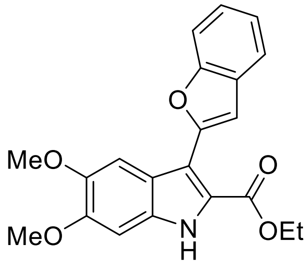

ethyl 3-(benzofuran-2-yl)-5,6-dimethoxy-1H-indole-2-carboxylate (**9l**): 96% yield (off-white solid). **^{1}H NMR** (400 MHz, $CDCl_3$) δ 8.98 (s, 1H), 7.71 (s, 1H), 7.65 (d, *J* = 7.3 Hz, 1H), 7.61 – 7.50 (m, 2H), 7.34 – 7.26 (m, 2H), 6.86 (s, 1H), 4.43 (q, *J* = 7.1 Hz, 2H), 3.99 (s, 3H), 3.96 (s, 3H), 1.41 (t, *J* = 7.1 Hz, 3H). **^{13}C NMR** (101 MHz, $CDCl_3$) δ 161.1, 154.6, 151.3, 150.8, 146.8, 130.9, 129.3, 124.1, 122.9, 121.9, 121.2, 120.0, 113.2, 111.0, 106.9, 103.7, 93.4, 61.2, 56.4, 56.2, 14.6. **HRMS** (ESI) calculated for $C_{21}H_{20}NO_5$ ($[M+H]^+$): 366.1336, found: 366.1336.

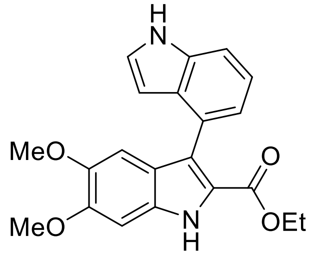

ethyl 5,6-dimethoxy-1H,1'H-[3,4'-biindole]-2-carboxylate (**9m**): 91% yield (white solid). **^{1}H NMR** (400 MHz, $CDCl_3$) δ 8.98 (s, 1H), 8.24 (s, 1H), 7.43 (d, J = 7.4 Hz, 1H), 7.33 – 7.26 (m, 2H), 7.17 (appt t, J = 2.8 Hz, 1H), 6.90 (s, 1H), 6.88 (s, 1H), 6.36 – 6.26 (m, 1H), 4.21 – 4.09 (m, 2H), 3.96 (s, 3H), 3.75 (s, 3H), 1.01 (t, J = 7.1 Hz, 3H). **^{13}C NMR** (101 MHz, $CDCl_3$) δ 161.9, 150.6, 148.4, 148.3, 146.5, 130.9, 126.5, 124.3, 122.9, 121.3, 121.1, 114.1, 110.9, 101.7, 93.7, 60.7, 56.3, 56.2, 56.1, 14.5. **HRMS** (ESI) calculated for $C_{21}H_{21}N_2O_4$ ($[M+H]^+$): 365.1496, found: 365.1492.

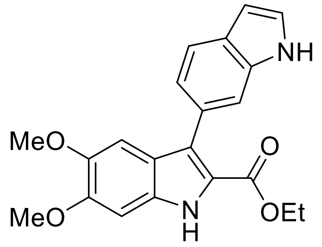

ethyl 5,6-dimethoxy-1H,1'H-[3,6'-biindole]-2-carboxylate (**9n**): 87% yield (off-white solid). **^{1}H NMR** (400 MHz, $CDCl_3$) δ 8.84 (s, 1H), 8.24 (s, 1H), 7.72 (d, J = 8.2 Hz, 1H), 7.59 (s, 1H), 7.32 (dd, J = 8.2, 1.4 Hz, 1H), 7. 26 – 7.24 (m, 1H), 7.02 (s, 1H), 6.87 (s, 1H), 6.63 – 6.60 (m, 1H), 4.25 (q, J = 7.1 Hz, 2H), 3.96 (s, 3H), 3.82 (s, 3H), 1.21 (t, J = 7.1 Hz, 3H). **^{13}C NMR** (101 MHz, $CDCl_3$) δ 162.0, 150.5, 146.4, 135.9, 130.9, 127.6, 127.2, 125.7, 124.7, 123.1, 121.4, 120.0, 113.0, 102.7, 102.1, 93.6, 60.6, 56.4, 56.2, 14.4. **HRMS** (ESI) calculated for $C_{21}H_{21}N_2O_4$ ($[M+H]^+$): 365.1496, found: 365.1492.

General Procedure for the Di-Suzuki Coupling of 7

To a 1-dram reaction vial with a septum-cap added XPhosPd G2 (4 mol%, 0.0024 mmol), **7** (1 equiv, 0.04 mmol) and corresponding boronic acid (2.4 equiv, 0.096 mmol), evacuated with vacuum and purged with N_2. To the reaction vial added THF (0.17 M) and 0.5 M K_3PO_4 (0.17 M). The reaction mixture was heated at 50 °C for 3.5 h and quenched with the addition of H_2O. The crude mixture was extracted with EtOAc (3 x 4 mL), dried with $MgSO_4$, filtered and concentrated *in vacuo*. The pure product was isolated via column chromatography (silica, hexanes: EtOAc: 2:1 to 9:1).

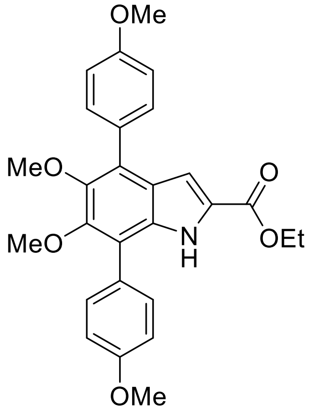

ethyl 5,6-dimethoxy-4,7-bis(4-methoxyphenyl)-1H-indole-2-carboxylate (**10a**): 95% yield (white solid). **^{1}H NMR** (400 MHz, $CDCl_3$) δ 8.64 (s, 1H), 7.56 (d, J = 8.1 Hz, 2H), 7.51 (d, J = 8.2 Hz, 2H), 7.11 (d, J = 2.2 Hz, 1H), 7.08 (d, J = 8.2 Hz, 2H), 7.05 (d, J = 8.3 Hz, 2H), 4.33 (q, J = 7.1 Hz, 2H), 3.91 (s, 3H), 3.90 (s, 3H), 3.74 (s, 3H), 3.66 (s, 3H), 1.34 (t, J = 7.1 Hz, 3H). **^{13}C NMR** (101 MHz, $CDCl_3$) δ 161.9, 159.4, 159.0, 150.0, 146.2, 133.0, 131.3, 130.9, 128.4, 127.8, 127.4, 126.1, 123.6, 118.4, 114.7, 113.9, 109.1, 61.6, 61.4, 61.1, 55.53, 55.45, 14.5. **HRMS** (ESI) calculated for $C_{27}H_{27}NO_6$ ($[M]^+$): 461.1838; found: 461.1826.

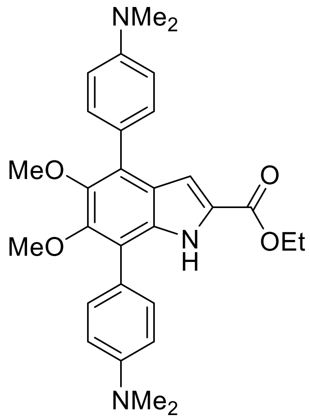

ethyl 4,7-bis(4-(dimethylamino)phenyl)-5,6-dimethoxy-1H-indole-2-carboxylate (**10b**): 95% yield (yellow solid). **^{1}H NMR** (400 MHz, $CDCl_3$) δ 8.71 (s, 1H), 7.54 (d, *J* = 8.5 Hz, 2H), 7.48 (d, *J* = 8.5 Hz, 2H), 7.19 (d, *J* = 2.2 Hz, 1H), 6.88 (dd, *J* = 8.5, 6.3 Hz, 4H), 4.32 (q, *J* = 7.1 Hz, 2H), 3.75 (s, 3H), 3.68 (s, 3H), 3.05 (s, 6H), 3.04 (s, 6H), 1.34 (t, *J* = 7.1 Hz, 3H). **^{13}C NMR** (101 MHz, $CDCl_3$) δ 162.0, 150.1, 150.0, 149.8, 146.1, 133.3, 130.9, 130.4, 127.4, 127.4, 124.1, 123.5, 121.7, 118.3, 112.9, 112.3, 109.5, 61.5, 61.3, 60.9, 40.7, 40.7, 14.5. **HRMS** (ESI) calculated for $C_{29}H_{34}N_3O_4$ ($[M+H]^+$): 488.2544, found: 488.2539.

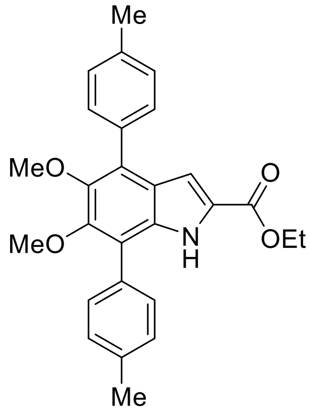

ethyl 5,6-dimethoxy-4,7-di-p-tolyl-1H-indole-2-carboxylate (**10c**): 84% yield (white solid). **^{1}H NMR** (400 MHz, $CDCl_3$) δ ^{1}H NMR (400 MHz, $CDCl_3$) δ 8.65 (s, 1H), 7.52 (d, *J* = 8.0 Hz, 2H), 7.48 (d, *J* = 8.0 Hz, 2H), 7.36 (d, *J* = 7.9 Hz, 2H), 7.32 (d, *J* = 7.9 Hz, 2H), 7.10 (d, *J* = 2.2 Hz, 1H), 4.32 (q, *J* = 7.1 Hz, 2H), 3.75 (s, 3H), 3.67 (s, 3H), 2.47 (s, 3H), 2.46 (s, 3H), 1.34 (t, *J* = 7.1 Hz, 3H). **^{13}C NMR** (101 MHz, $CDCl_3$) δ 161. 9, 149.9, 146.2, 137.8, 137.2, 133.1, 132.9, 131.0, 130.0, 129.9, 129.6, 129.1, 127.9, 127.8, 123.5, 118.8, 109.1, 61.6, 61.4, 61.0, 21.5, 14.5. **HRMS** (ESI) calculated for $C_{27}H_{28}NO_4$ ($[M+H]^+$): 430.2013, found: 430.2010.

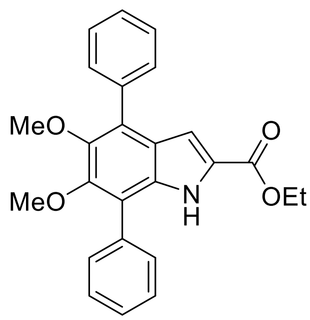

ethyl 5,6-dimethoxy-4,7-diphenyl-1H-indole-2-carboxylate (**10d**). 95% yield (white solid). **^{1}H NMR** (400 MHz, $CDCl_3$) δ 8.67 (s, 1H), 7.66 – 7.40 (m, 10H), 7.09 (d, *J* = 2.2 Hz, 1H), 4.33 (q, *J* = 7.1 Hz, 2H), 3.75 (s, 3H), 3.67 (s, 3H), 1.34 (t, *J* = 7.1 Hz, 3H). **^{13}C NMR** (101 MHz, $CDCl_3$) δ 161.9, 149.9, 146.2, 136.1, 134.0, 132.8, 130.2, 129.8, 129.2, 128.4, 128.2, 128.1, 128.0, 127.5, 123.6, 119.1, 109.0, 61.7, 61.5, 61.1, 14.5. . **HRMS** (ESI) calculated for $C_{25}H_{24}NO_4$ ($[M+H]^+$): 402.1700, found: 402.1699.

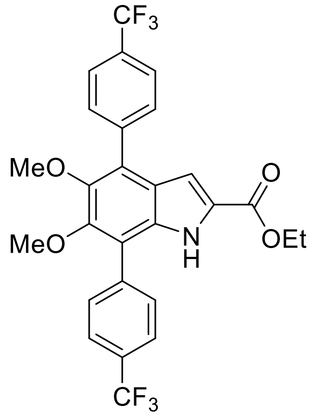

ethyl 5,6-dimethoxy-4,7-bis(4-(trifluoromethyl)phenyl)-1H-indole-2-carboxylate (**10e**). 92% yield (white solid). **^{1}H NMR** (400 MHz, $CDCl_3$) δ 8.64 (s, 1H), 7.83 (d, *J* = 8.1 Hz, 2H), 7.75 (dd, *J* = 16.8, 7.6 Hz, 7H), 7.05 (d, *J* = 2.2 Hz, 1H), 4.34 (q, *J* = 7.1 Hz, 2H), 3.76 (s, 3H), 3.68 (s, 3H), 1.36 (t, *J* = 7.1 Hz, 3H). **^{13}C NMR** (101 MHz, $CDCl_3$) δ 161.6, 149.9, 146.3, 139.6, 137.6, 132.3, 130.5 (2C), 130.4 ($^2J_{C\text{-}F}$ = 32 Hz), 130.2 (2C), 129.7 ($^2J_{C\text{-}F}$ = 32 Hz), 128.6, 127.4, 126.2 ($^3J_{C\text{-}F}$ = 4 Hz), 125.4 ($^3J_{C\text{-}F}$ = 4 Hz), 124.4 ($^1J_{C\text{-}F}$ = 272 Hz), 124.2 ($^1J_{C\text{-}F}$ = 272 Hz), 123.5, 118.4, 108.5, 61.8, 61.6, 61.4, 14.5. **HRMS** (ESI) calculated for $C_{27}H_{22}NO_4F_6$ ($[M+H]^+$): 538.1448, found: 538.1444.

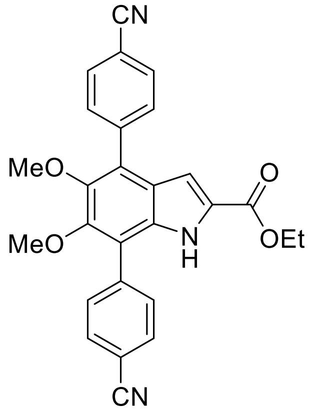

ethyl 4,7-bis(4-cyanophenyl)-5,6-dimethoxy-1H-indole-2-carboxylate (**10f**): 76% yield (white solid). **^{1}H NMR** (400 MHz, $CDCl_3$) δ ^{1}H NMR (400 MHz, $CDCl_3$) δ 8.67 (s, 1H), 7.86 (d, *J* = 8.1 Hz, 2H), 7.82 (d, *J* = 8.1 Hz, 2H), 7.78 – 7.69 (m, 4H), 7.03 (d, *J* = 2.1 Hz, 1H), 4.35 (q, *J* = 7.1 Hz, 2H), 3.75 (s, 3H), 3.66 (s, 3H), 1.36 (t, *J* = 7.1 Hz, 3H). **^{13}C NMR** (101 MHz, $CDCl_3$) δ 161.5, 149.8, 146.3, 140.7, 138.7, 133.0, 132.9, 132.3, 132.0, 131.0, 130.6, 128.9, 128.1, 127.3, 123.3, 119.0, 118.7, 118.3, 112.2, 111.5, 108.2, 61.8, 61.6, 61.5, 14.5. **HRMS** (ESI) calculated for $C_{27}H_{22}N_3O_4$ ($[M+H]^+$): 452.1605, found: 452.1598.

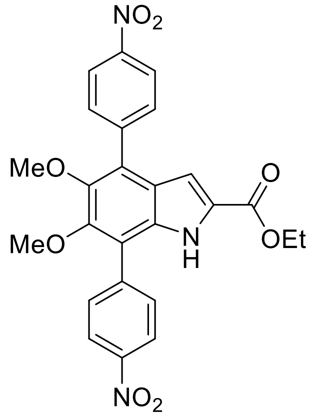

ethyl 5,6-dimethoxy-4,7-bis(4-nitrophenyl)-1H-indole-2-carboxylate (**10g**): 90% yield (yellow solid). **^{1}H NMR** (400 MHz, $CDCl_3$) δ 8.73 (s, 1H), 8.43 (d, *J* = 8.7 Hz, 2H), 8.39 (d, *J* = 8.7 Hz, 2H), 7.82 (d, *J* = 8.7 Hz, 2H), 7.80 (d, *J* = 8.7 Hz, 2H), 7.05 (d, *J* = 2.1 Hz, 1H), 4.35 (q, *J* = 7.1 Hz, 2H), 3.77 (s, 3H), 3.68 (s, 3H), 1.36 (t, *J* = 7.1 Hz, 3H). **^{13}C NMR** (101 MHz, $CDCl_3$) δ 161.5, 149.9, 147.7, 147.4, 146.4, 142.7, 140.6, 132.0, 131.1, 130.8, 129.1, 127.1, 124.6, 123.7, 123.3, 118.2, 108.2, 61.8, 61.6, 61.5, 14.5. **HRMS** (ESI) calculated for $C_{25}H_{22}N_3O_8$ ($[M+H]^+$): 492.1401, found: 492.1394.

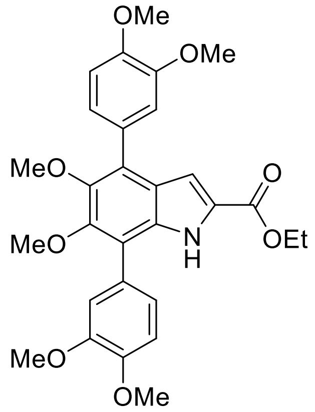

ethyl 4,7-bis(3,4-dimethoxyphenyl)-5,6-dimethoxy-1H-indole-2-carboxylate (**10h**): 95% yield (white solid). **^{1}H NMR** (400 MHz, $CDCl_3$) δ 8.70 (s, 1H), 7.21 – 7.10 (m, 5H), 7.08 – 7.01 (m, 2H), 4.33 (q, *J* = 7.1 Hz, 2H), 3.99 (s, 3H), 3.98 (s, 3H), 3.93 (s, 3H), 3.92 (s, 3H), 3.75 (s, 3H), 3.68 (s, 3H), 1.35 (t, *J* = 7.1 Hz, 3H). **^{13}C NMR** (101 MHz, $CDCl_3$) δ 161.8, 149.9, 149.5, 148.9, 148.8, 148.5, 146.1, 132.9, 128.7, 127.8, 127.6, 126.4, 123.6, 122.5, 121.8, 118.5, 113.5, 113.1, 111.8, 111.1, 109.0, 61.6, 61.4, 61.1, 56.3, 56.1, 56.0, 14.51. **HRMS** (ESI) calculated for $C_{29}H_{32}NO_8$ ($[M+H]^+$): 522.2122, found: 522.2122.

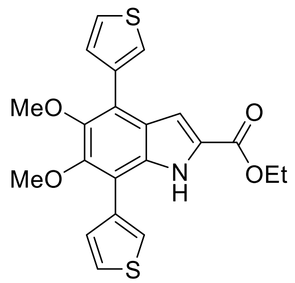

ethyl 5,6-dimethoxy-4,7-di(thiophen-3-yl)-1H-indole-2-carboxylate (**10i**): 99% yield (off-white solid). **^{1}H NMR** (400 MHz, $CDCl_3$)δ 8.81 (s, 1H), 7.65 – 7.57 (m, 2H), 7.56 – 7.52 (m, 1H), 7.49 (d, *J* = 4.8 Hz, 1H), 7.46 (dd, *J* = 5.0, 3.0 Hz, 1H), 7.42 (d, *J* = 4.8 Hz, 1H), 4.36 (q, *J* = 7.1 Hz, 2H), 3.78 (s, 3H), 3.71 (s, 3H), 1.38 (t, *J* = 7.1 Hz, 3H). **^{13}C NMR** (101 MHz, $CDCl_3$) δ 161.8, 150.2, 146.4, 135.9, 133.8, 132.8, 129.5, 128.8, 127.9, 126.4, 124.9, 124.5, 124.1, 123.3, 122.9, 114.0, 109.2, 61.5, 61.3, 61.2, 14.5. **HRMS** (ESI) calculated for $C_{21}H_{20}NO_4S_2$ ($[M+H]^+$): 414.0828, found: 414.0827.

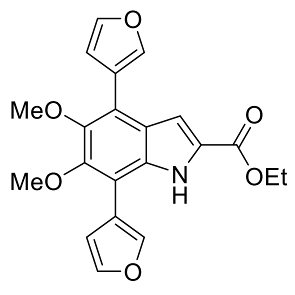

ethyl 4,7-di(furan-3-yl)-5,6-dimethoxy-1H-indole-2-carboxylate (**10j**): 81% yield (off-white solid). **^{1}H NMR** (400 MHz, $CDCl_3$) δ 8.84 (s, 1H), 7.96 – 7.89 (m, 1H), 7.88 – 7.82 (m, 1H), 7.64 (d, J = 1.6 Hz, 1H), 7.58 (d, J = 1.6 Hz, 1H), 7.34 (d, J = 2.1 Hz, 1H), 6.95 – 6.90 (m, 1H), 6.85 – 6.77 (m, 1H), 4.39 (q, J = 7.1 Hz, 2H), 3.84 (s, 3H), 3.78 (s, 3H), 1.40 (t, J = 7.1 Hz, 3H). **^{13}C NMR** (101 MHz, $CDCl_3$) δ 161.8, 150.3, 146.5, 143.8, 142.9, 141.9, 141.1, 132.7, 127.9, 122.8, 119.9, 119.0, 117.9, 111.8, 111.2, 109.9, 109.2, 61.3, 61.2, 61.0, 14.6. **HRMS** (ESI) calculated for $C_{21}H_{20}NO_6$ ($[M+H]^+$): 382.1285, found: 382.1282.

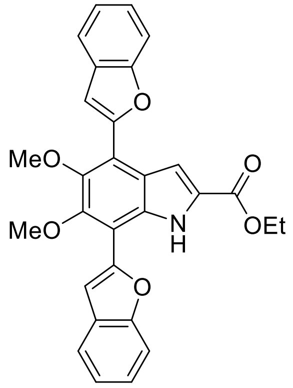

ethyl 4,7-di(benzofuran-2-yl)-5,6-dimethoxy-1H-indole-2-carboxylate (**10k**): 91% yield (light green solid). **^{1}H NMR** (400 MHz, $CDCl_3$) δ 10.34 (s, 1H), 8.03 (d, J = 2.4 Hz, 1H), 7.73 – 7.63 (m, 4H), 7.61 (d, J = 1.0 Hz, 1H), 7.57 (d, J = 1.0 Hz, 1H), 7.41 – 7.27 (m, 4H), 4.50 (q, J = 7.2 Hz, 2H), 4.08 (s, 3H), 3.99 (s, 3H), 1.50 (t, J = 7.1 Hz, 3H). **^{13}C NMR** (101 MHz, $CDCl_3$) δ 161.0, 154.6, 153.7, 151.5, 151.0, 150.0, 147.3, 129.3, 129.0, 127.6, 124.9, 124.2, 123.6, 123.6, 123.5, 122.9, 121.6, 121.2, 112.4, 110.99, 110.97, 108.7, 107.5, 107.1, 105.3, 61.4, 60.3, 56.4, 14.5. **HRMS** (ESI) calculated for $C_{29}H_{24}NO_6$ ($[M+H]^+$): 482.1598, found: 482.1595.

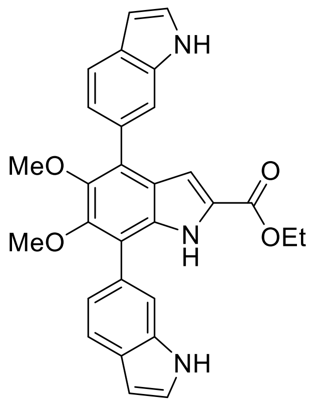

ethyl 5',6'-dimethoxy-1H,1'H,1''H-[6,4':7',6''-terindole]-2'-carboxylate (**10l**): 78% yield (white solid). **^{1}H NMR** (400 MHz, $CDCl_3$) δ 8.72 (s, 1H), 8.38 (s, 3H), 8.35 (s, 2H), 7.82 (d, J = 8.1 Hz, 1H), 7.78 (d, J = 8.1 Hz, 1H), 7.68 (s, 1H), 7.63 (s, 1H), 7.43 (dd, J = 8.1, 1.4 Hz, 1H), 7.36 (dd, J = 8.1, 1.4 Hz, 1H), 7.34 – 7.27 (m, 2H), 7.16 (d, J = 2.2 Hz, 1H), 6.70 – 6.60 (m, 2H), 4.29 (q, J = 7.1 Hz, 2H), 3.73 (s, 3H), 3.66 (s, 3H), 1.30 (t, J = 7.1 Hz, 3H). **^{13}C NMR** (101 MHz, $CDCl_3$) δ 162.0, 150.0, 146.2, 133.3, 130.0, 128.6, 127.7, 127.6, 127.3, 125.3, 124.9, 123.9, 122.6, 121.7, 121.4, 120.4, 119.6, 112.7, 112.4, 109.5, 102.8, 102.7, 61.7, 61.5, 61.0, 14.5. **HRMS** (ESI) calculated for $C_{29}H_{26}N_3O_4$ ($[M+H]^+$): 480.1918, found: 480.1916.

References

1. H. Deng, Y. Fang, *ACS Med. Chem. Lett.* 2012, **3**, 550-554.

2. M. Tani, M. Goto, M. Shimizu, Y. Mochimaru, J. Amemiya, N. Mizuno, R. Sato, Y. Murakami, *Synlett* 1996, 931-932.

3. P. Buchgraber, M. M. Domostoj, B. Scheiper, C. Wirtz, R. Mynott, J. Rust, A. Furstner, *Tetrahedron* 2009, **65**, 6519-6534.

NMR Spectra

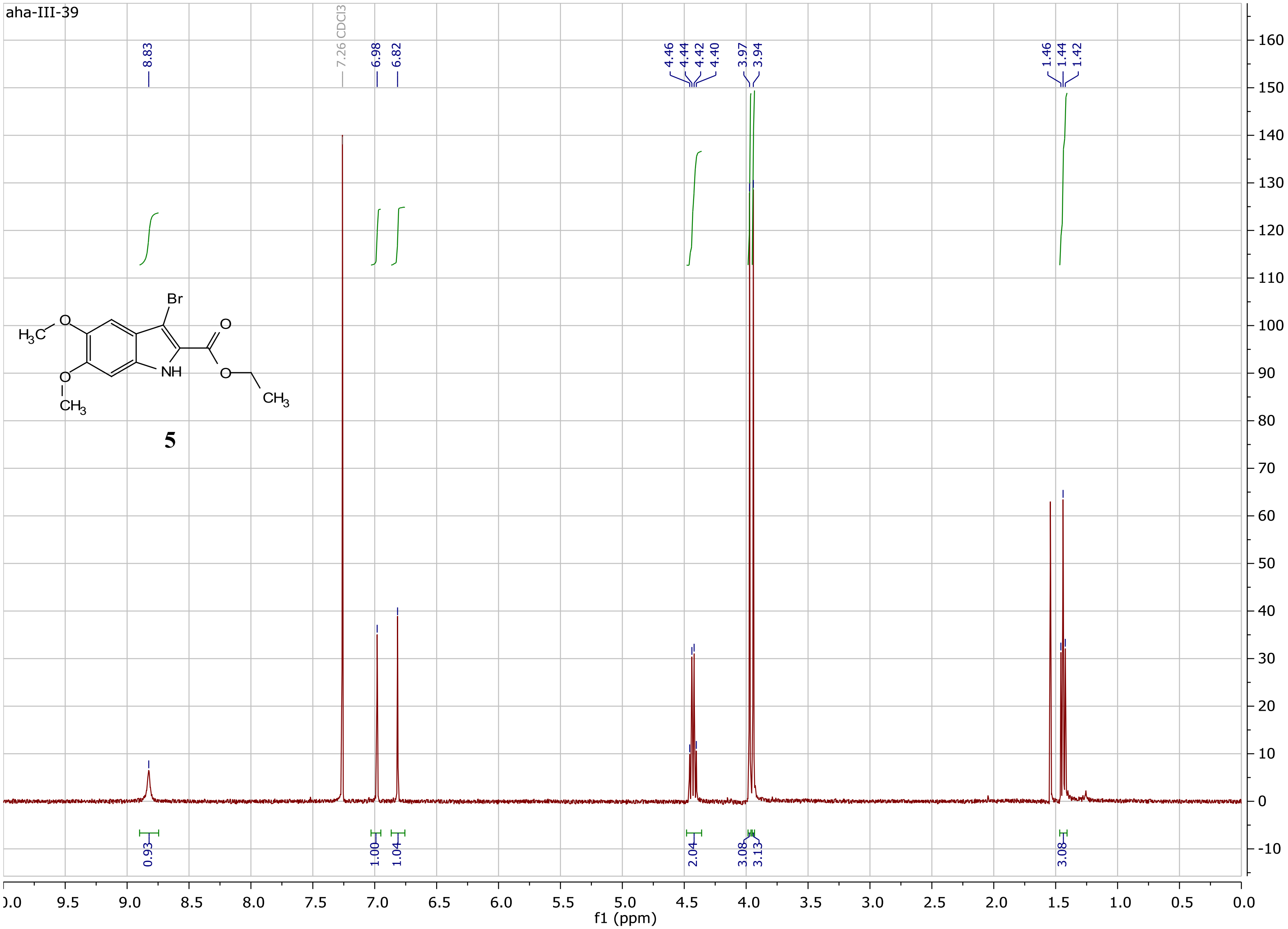
aha-III-39
8.83
7.26 CDCl3
6.98
6.82
4.46
4.44
4.42
4.40
3.97
3.94
1.46
1.44
1.42
Br
O
H3C
NH
O
O
O
CH3
CH3
5
0.93
1.00
1.04
2.04
3.08
3.13
3.08
f1 (ppm)

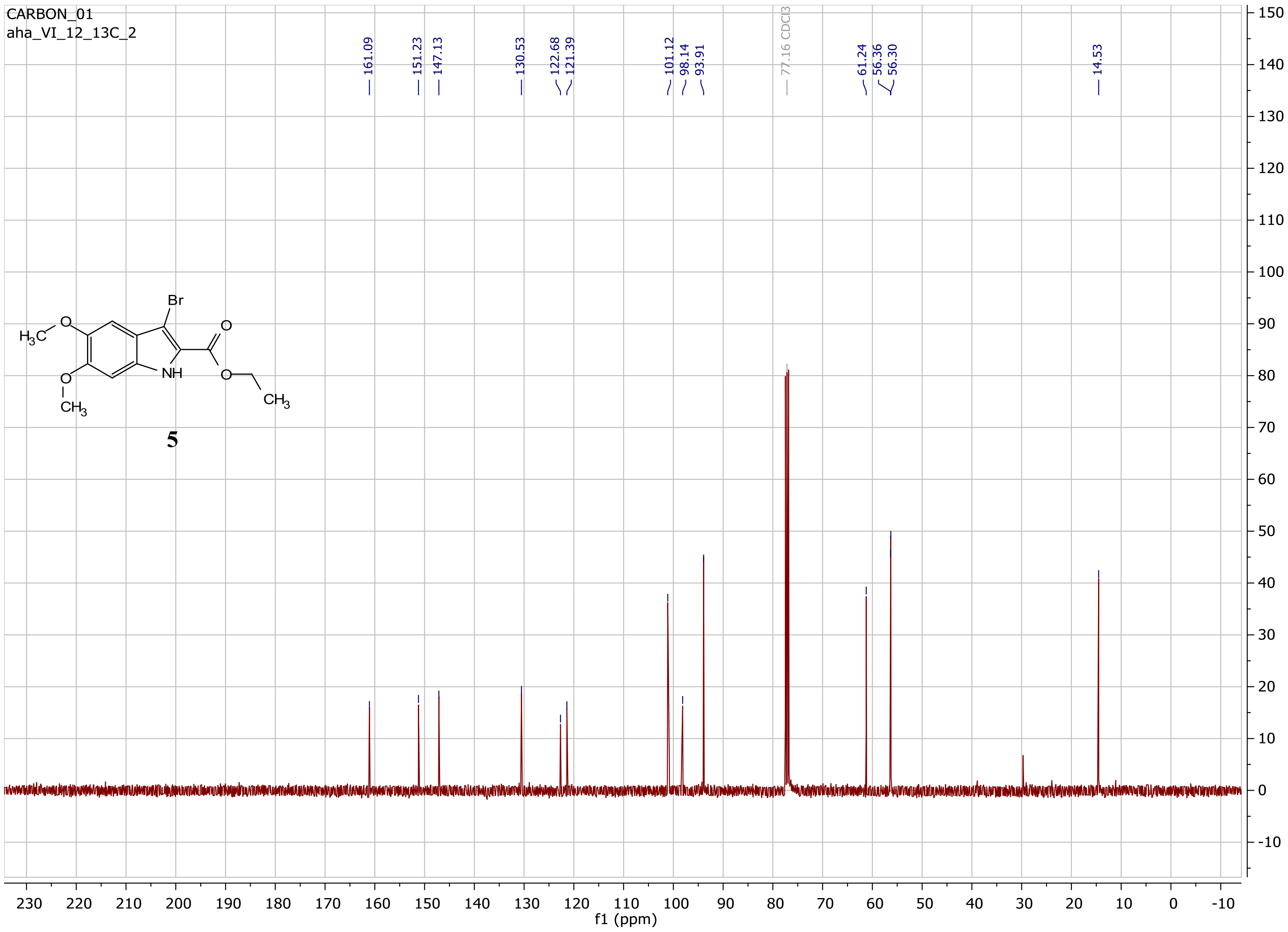

CARBON_01
aha_VI_12_13C_2
161.09
151.23
147.13
130.53
122.68
121.39
101.12
98.14
93.91
77.16 CDCl3
61.24
56.36
56.30
14.53
Br
O
H3C
O
NH
O
O
CH3
CH3
5
f1 (ppm)

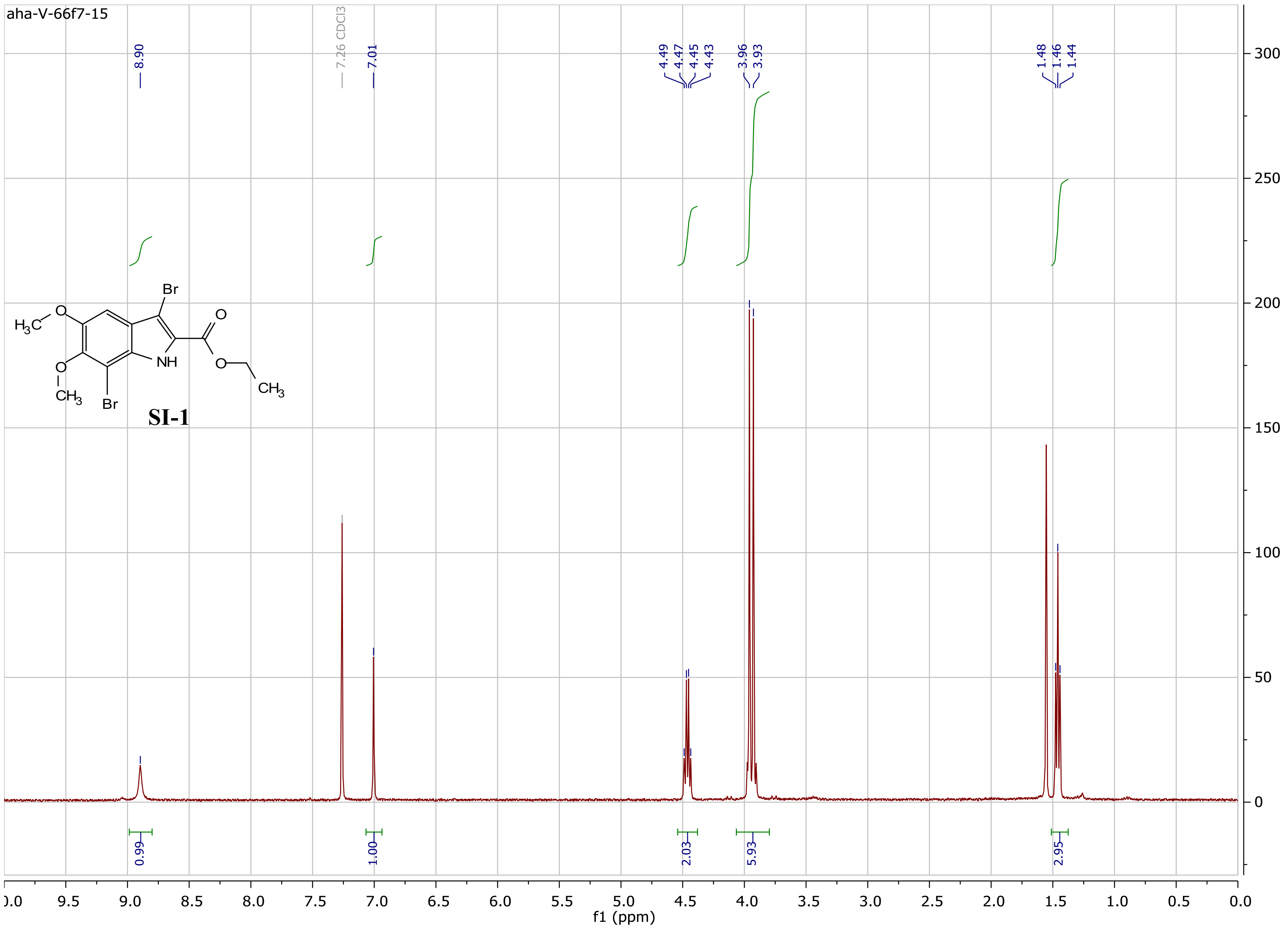
aha-V-66f7-15
8.90
7.26 CDCl3
7.01
4.49
4.47
4.45
4.43
3.96
3.93
1.48
1.46
1.44
Br
O
H3C
O
O
NH
O
CH3
CH3
Br
0.99
1.00
2.03
5.93
2.95
f1 (ppm)

SI-1

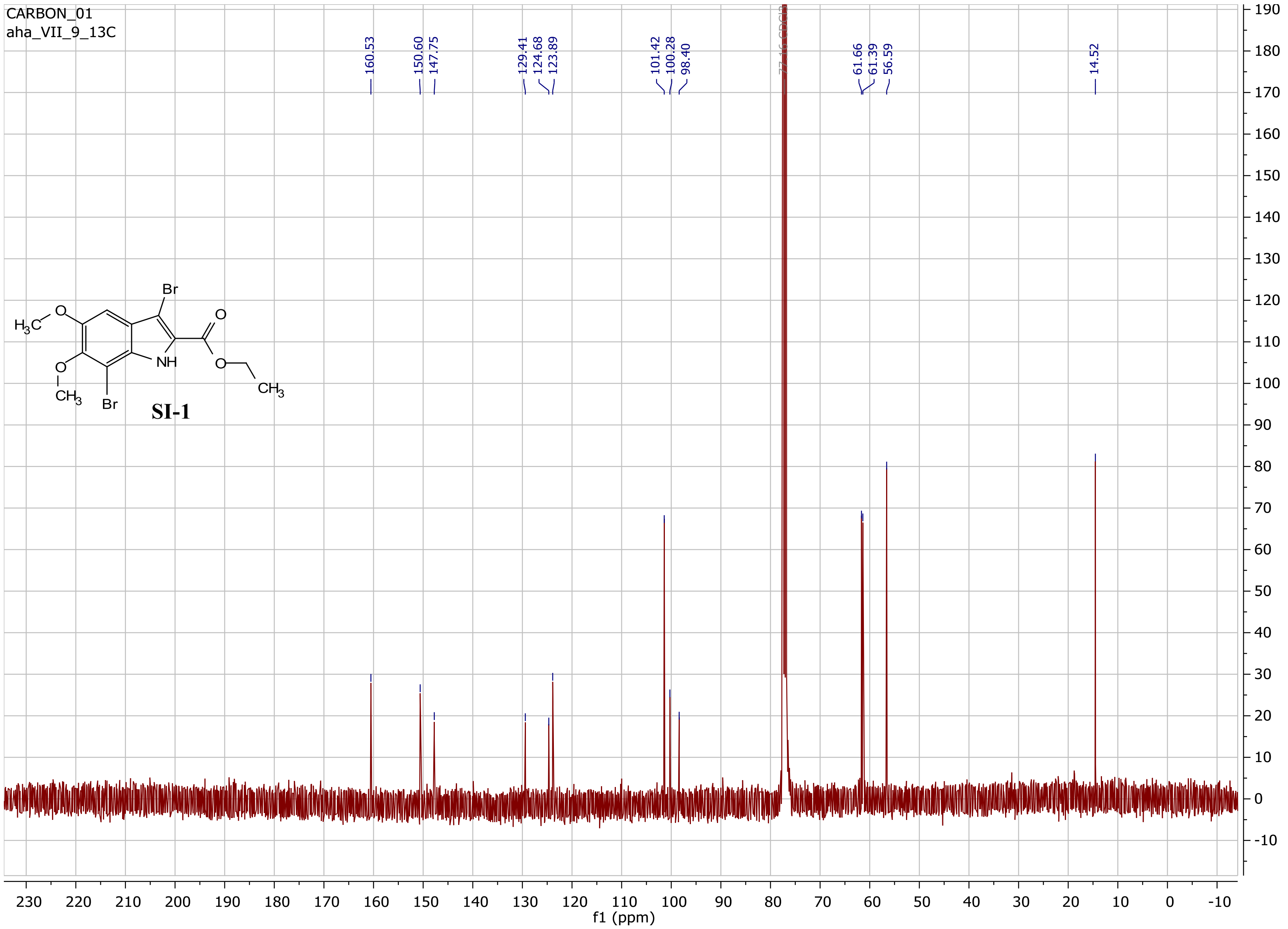
CARBON_01
aha_VII_9_13C
160.53
150.60
147.75
129.41
124.68
123.89
101.42
100.28
98.40
61.66
61.39
56.59
14.52
Br
O
NH
O
Br
SI-1
f1 (ppm)

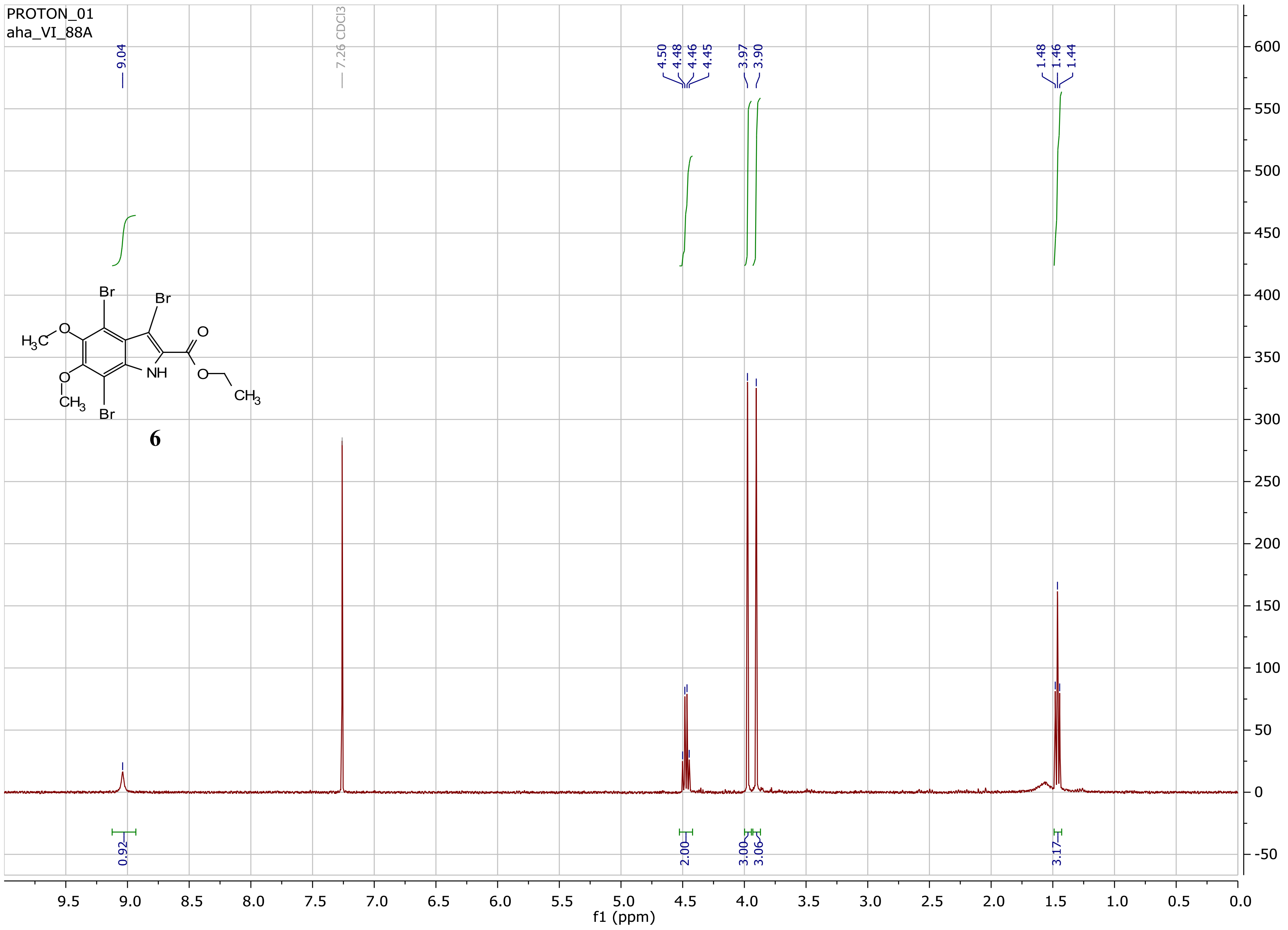
PROTON_01
aha_VI_88A
9.04
7.26 CDCl3
4.50
4.48
4.46
4.45
3.97
3.90
1.48
1.46
1.44
6
0.92
2.00
3.00
3.06
3.17
f1 (ppm)

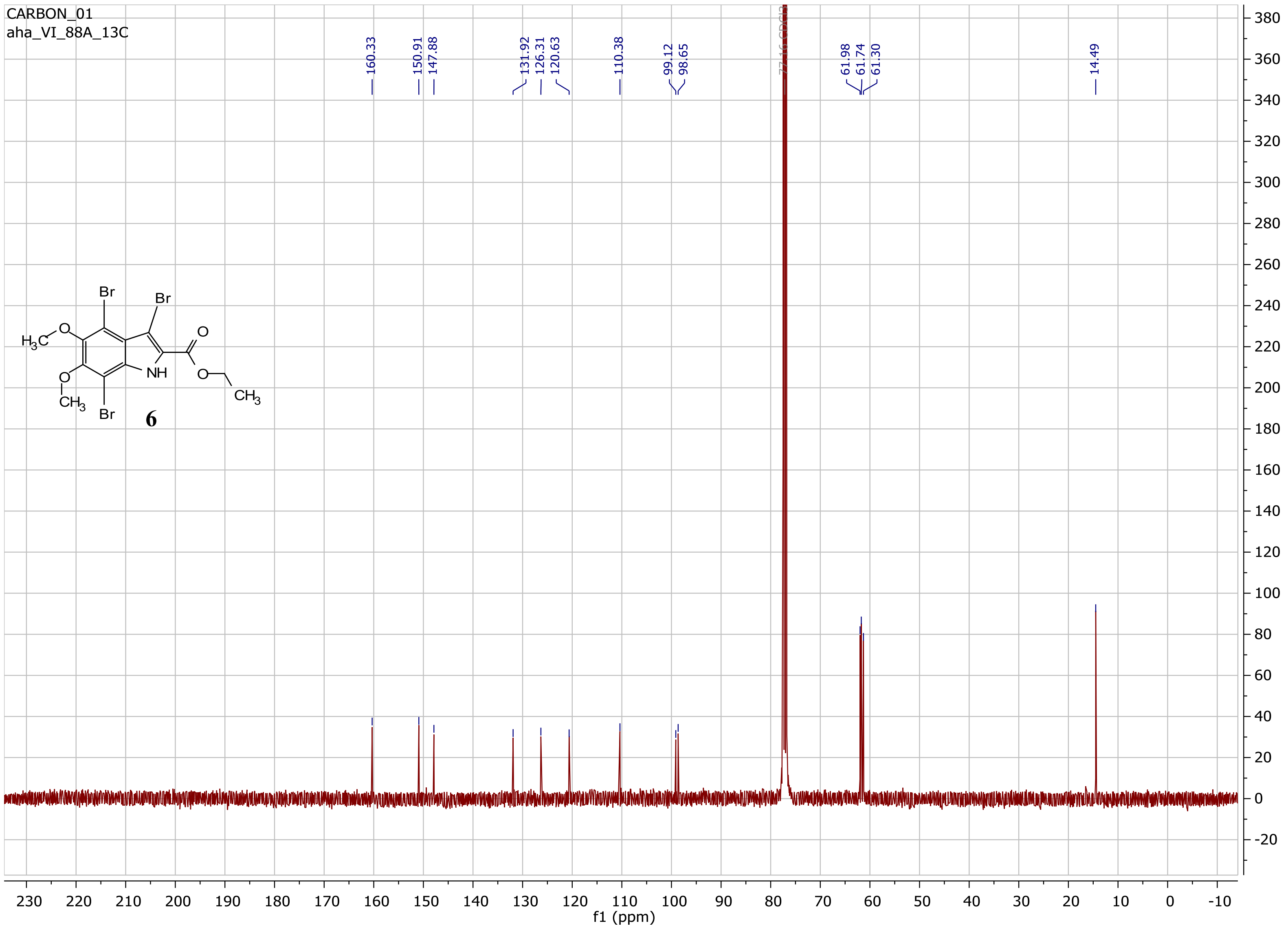

CARBON_01
aha_VI_88A_13C
160.33
150.91
147.88
131.92
126.31
120.63
110.38
99.12
98.65
61.98
61.74
61.30
14.49
Br
Br
O
H_3C
O
NH
O
O
CH_3
CH_3
Br
6
f1 (ppm)

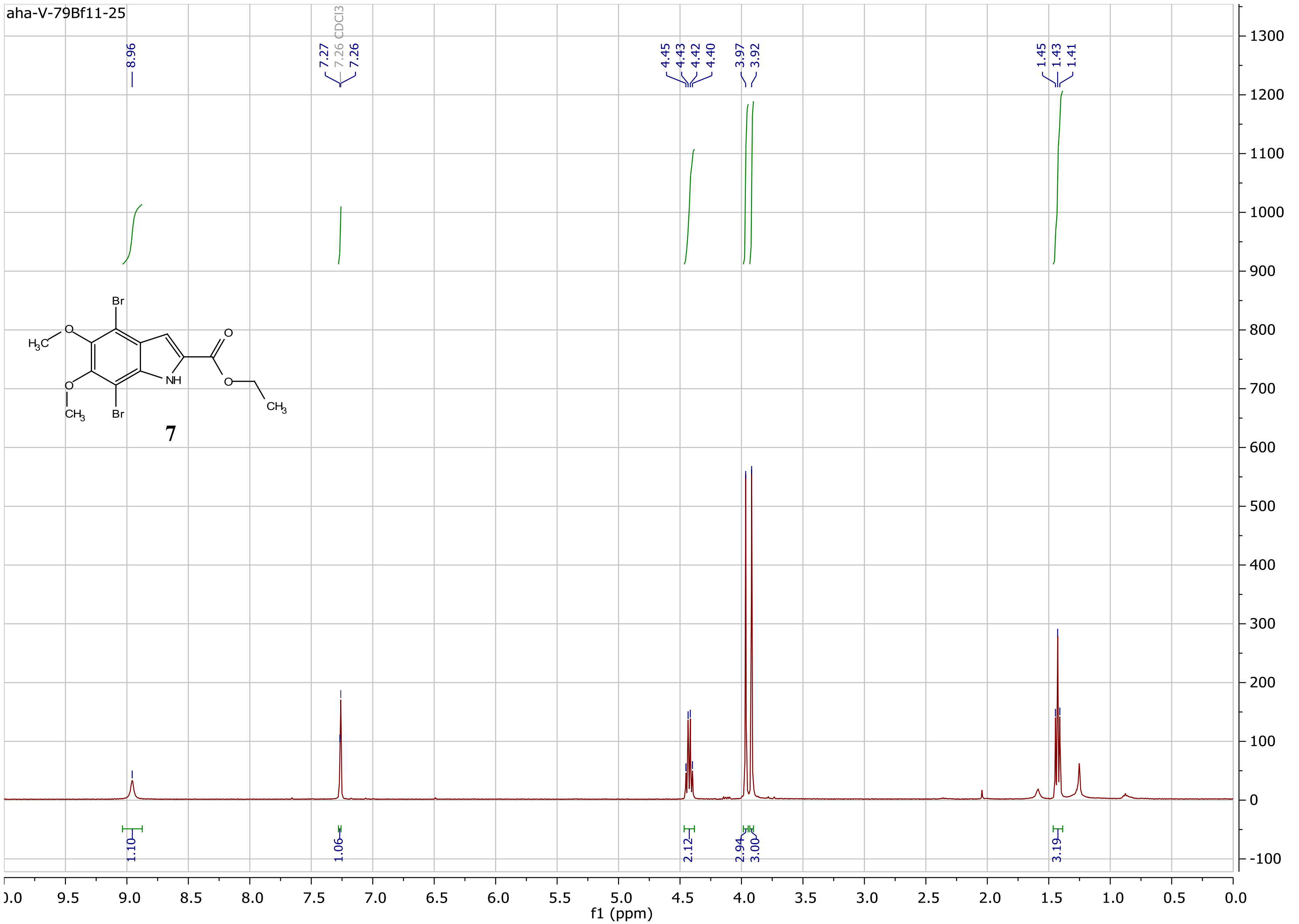
aha-V-79Bf11-25
8.96
7.27
7.26 CDCl3
7.26
4.45
4.43
4.42
4.40
3.97
3.92
1.45
1.43
1.41
Br
H_3C
O
O
NH
O
O
CH_3
Br
CH_3
7
1.10
1.06
2.12
2.94
3.00
3.19
f1 (ppm)

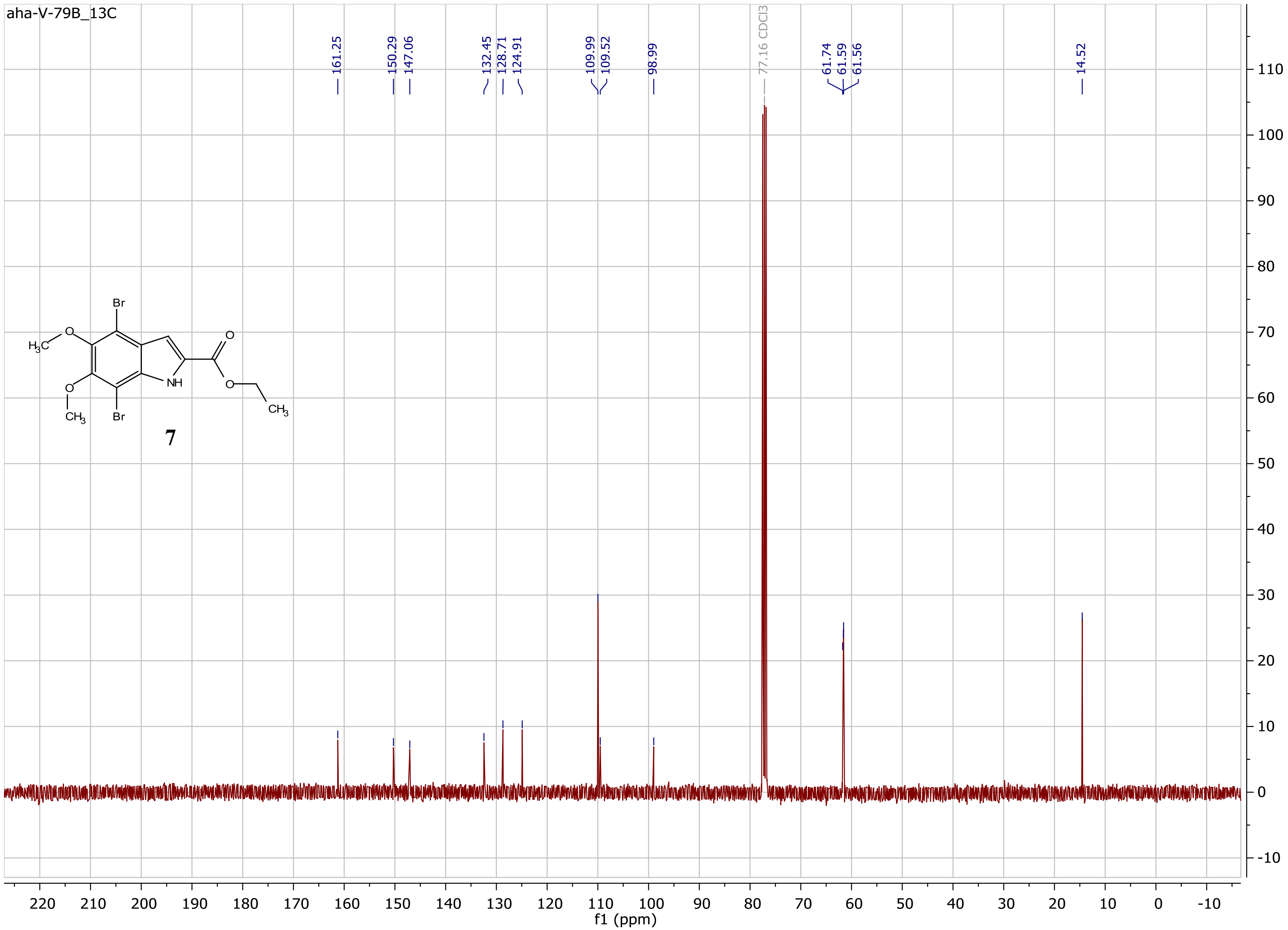

aha-V-79B_13C
161.25
150.29
147.06
132.45
128.71
124.91
109.99
109.52
98.99
77.16 CDCl3
61.74
61.59
61.56
14.52
Br
O
H3C
O
O
NH
O
CH3
CH3
Br
7
f1 (ppm)

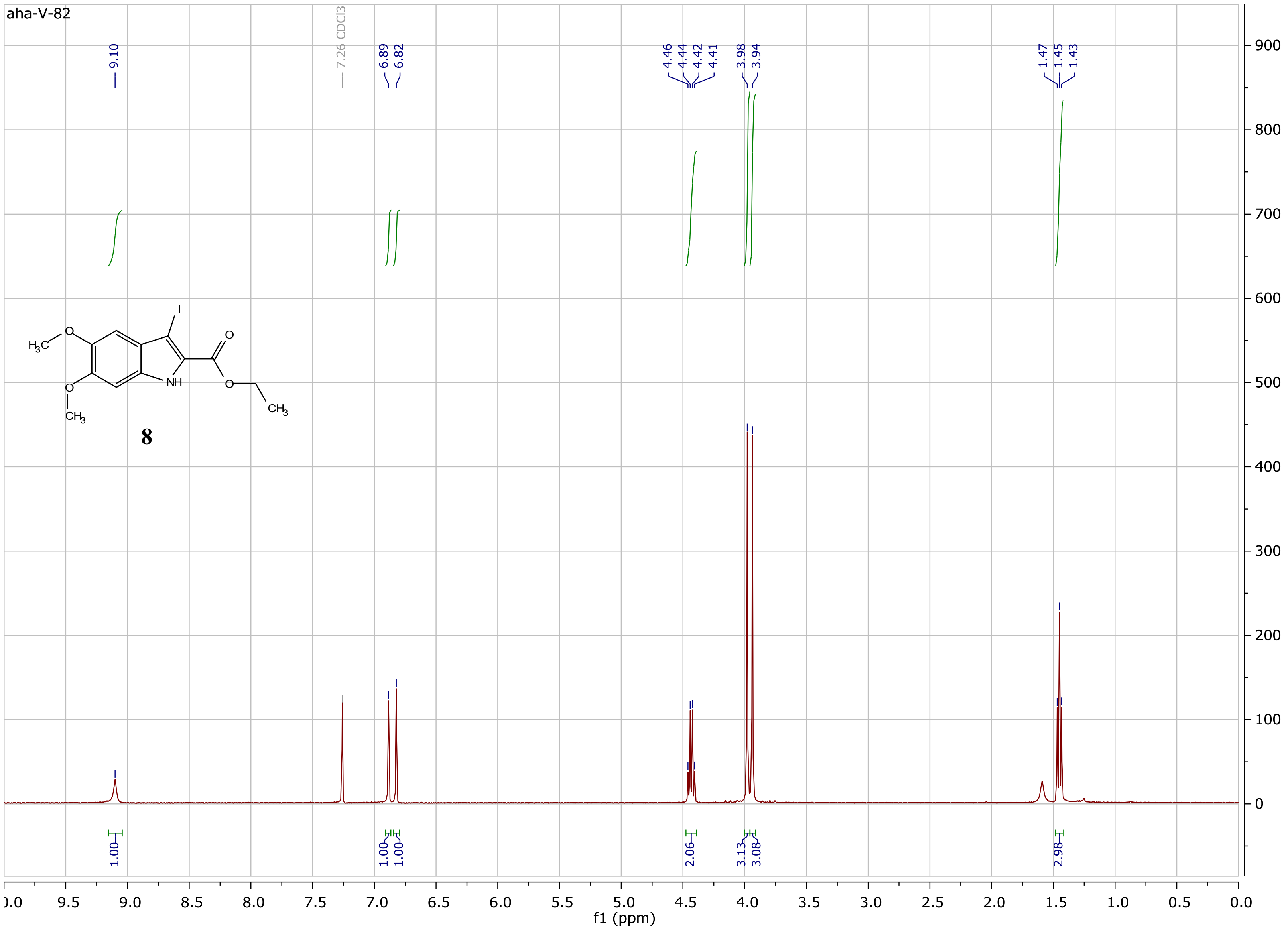
aha-V-82
9.10
7.26 CDCl3
6.89
6.82
4.46
4.44
4.42
4.41
3.98
3.94
1.47
1.45
1.43
1.00
1.00
1.00
2.06
3.13
3.08
2.98
8
f1 (ppm)

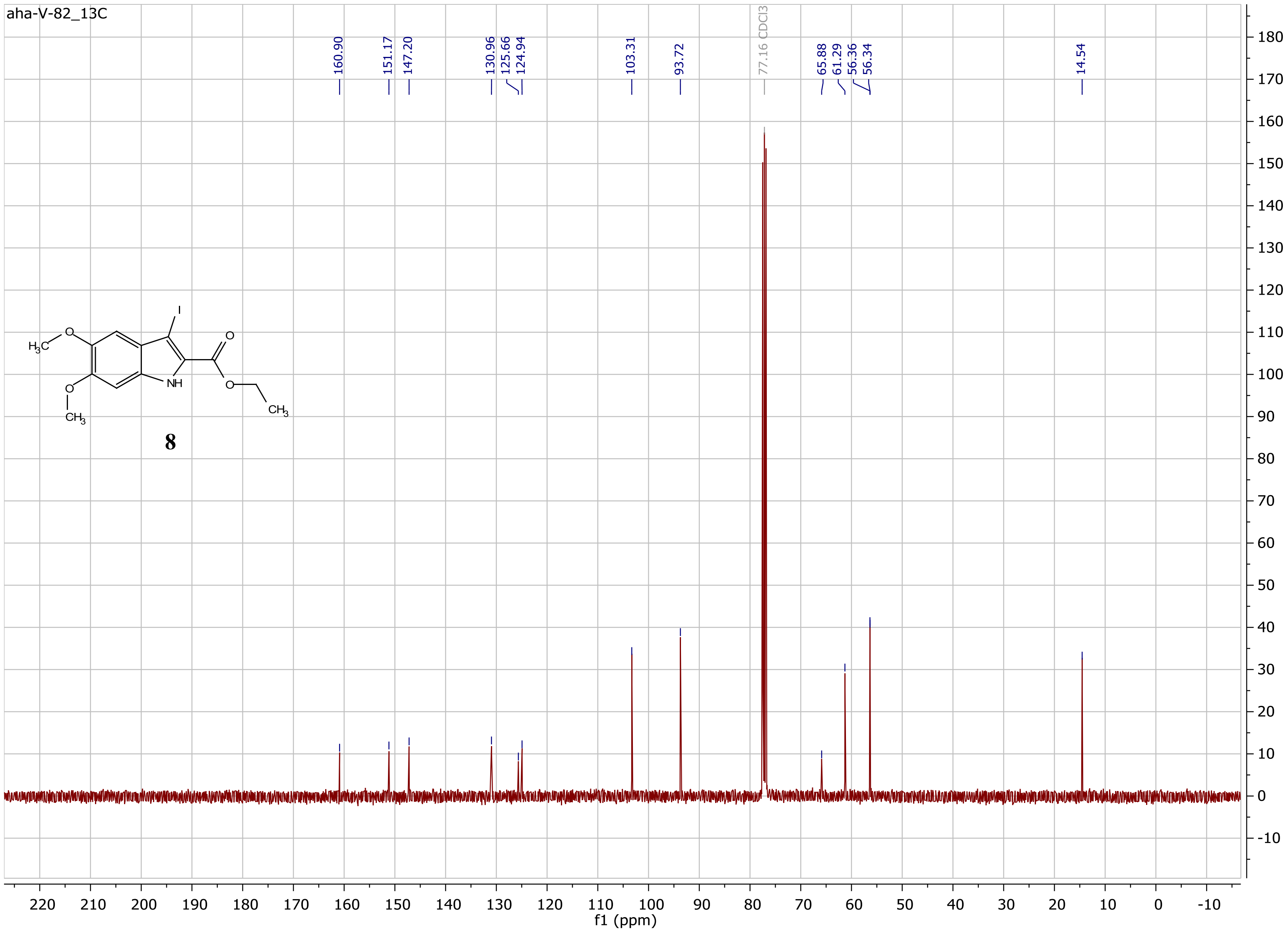

aha-V-82_13C
160.90
151.17
147.20
130.96
125.66
124.94
103.31
93.72
77.16 CDCl3
65.88
61.29
56.36
56.34
14.54
I
O
H_3C
O
O
NH
O
CH_3
CH_3
8
f1 (ppm)

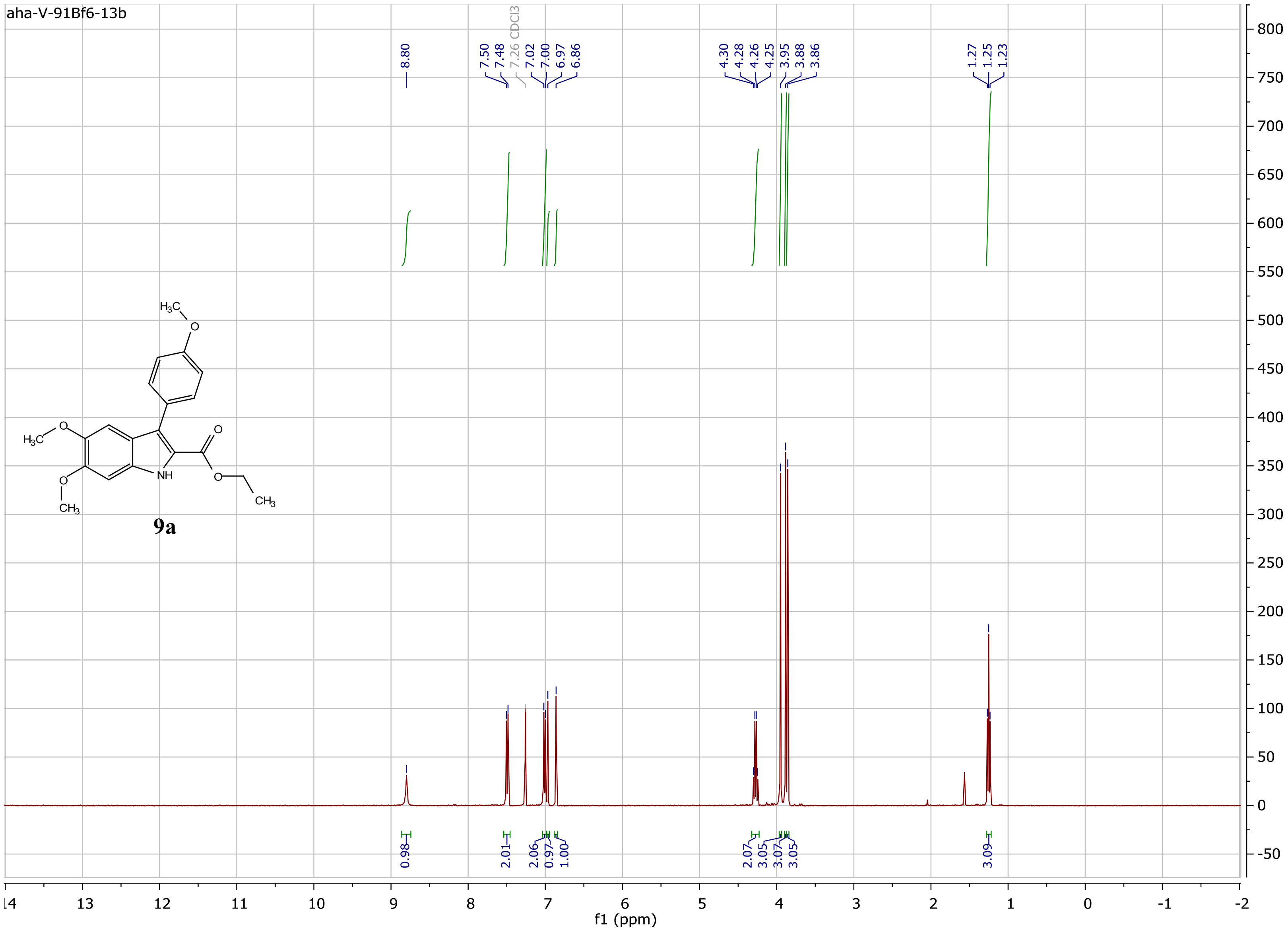
aha-V-91Bf6-13b
8.80
7.50
7.48
7.26 CDCl3
7.02
7.00
6.97
6.86
4.30
4.28
4.26
4.25
3.95
3.88
3.86
1.27
1.25
1.23
0.98
2.01
2.06
0.97
1.00
2.07
3.05
3.07
3.05
3.09
9a
f1 (ppm)

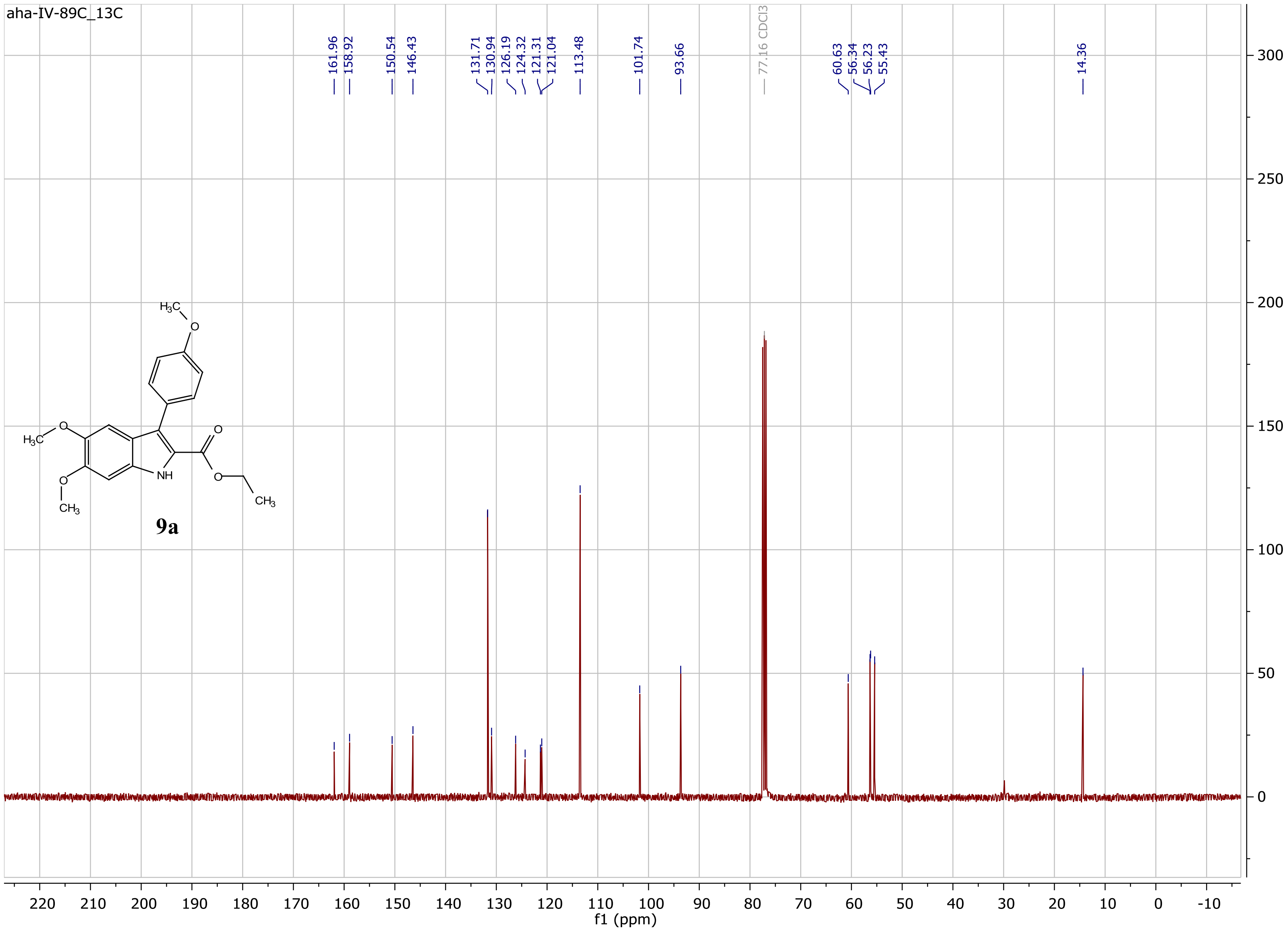
aha-IV-89C_13C
161.96
158.92
150.54
146.43
131.71
130.94
126.19
124.32
121.31
121.04
113.48
101.74
93.66
77.16 CDCl3
60.63
56.34
56.23
55.43
14.36
9a
f1 (ppm)

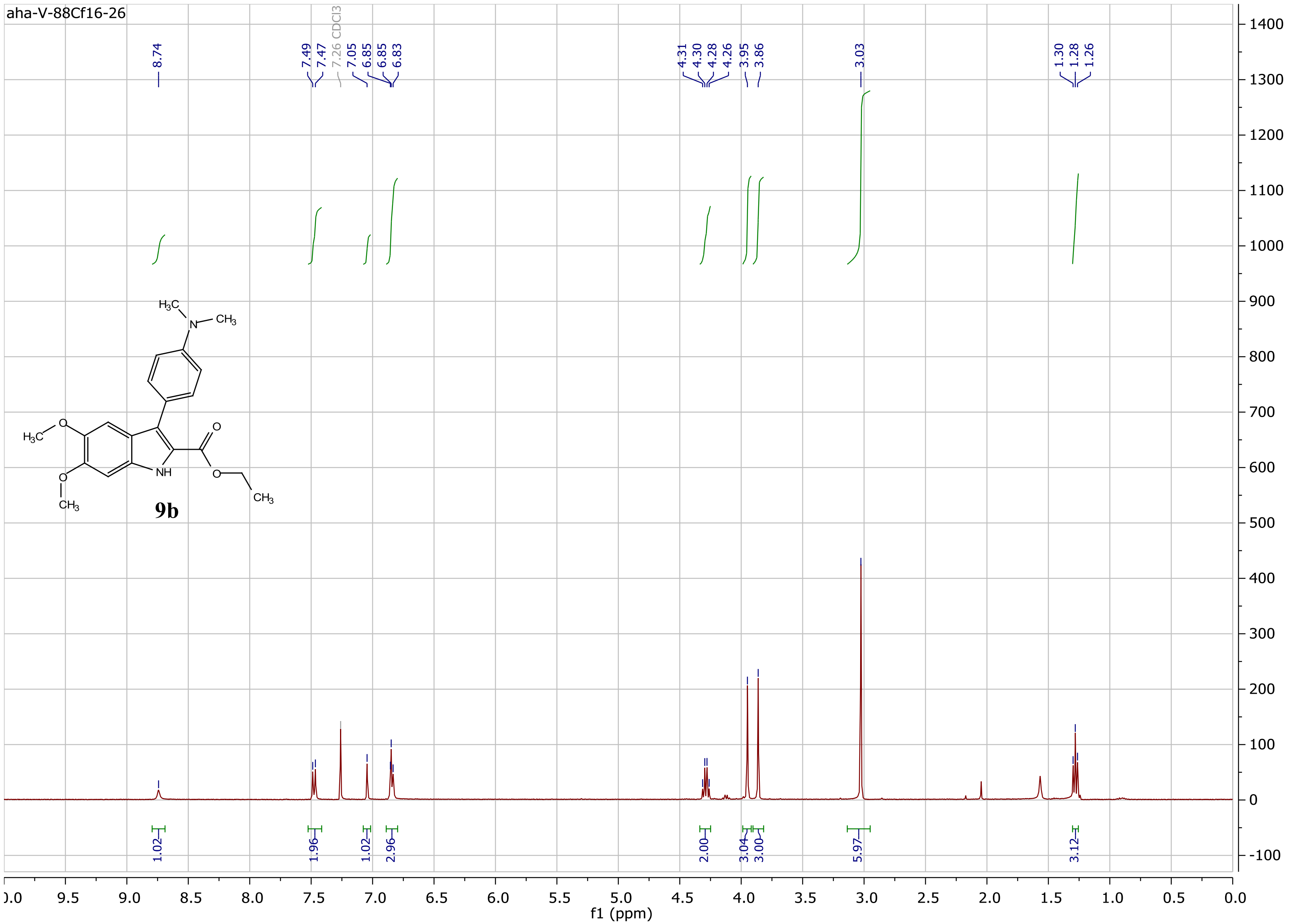
aha-V-88Cf16-26
8.74
7.49
7.47
7.26 CDCl3
7.05
6.85
6.85
6.83
4.31
4.30
4.28
4.26
3.95
3.86
3.03
1.30
1.28
1.26
1.02
1.96
1.02
2.96
2.00
3.04
3.00
5.97
3.12
9b
f1 (ppm)

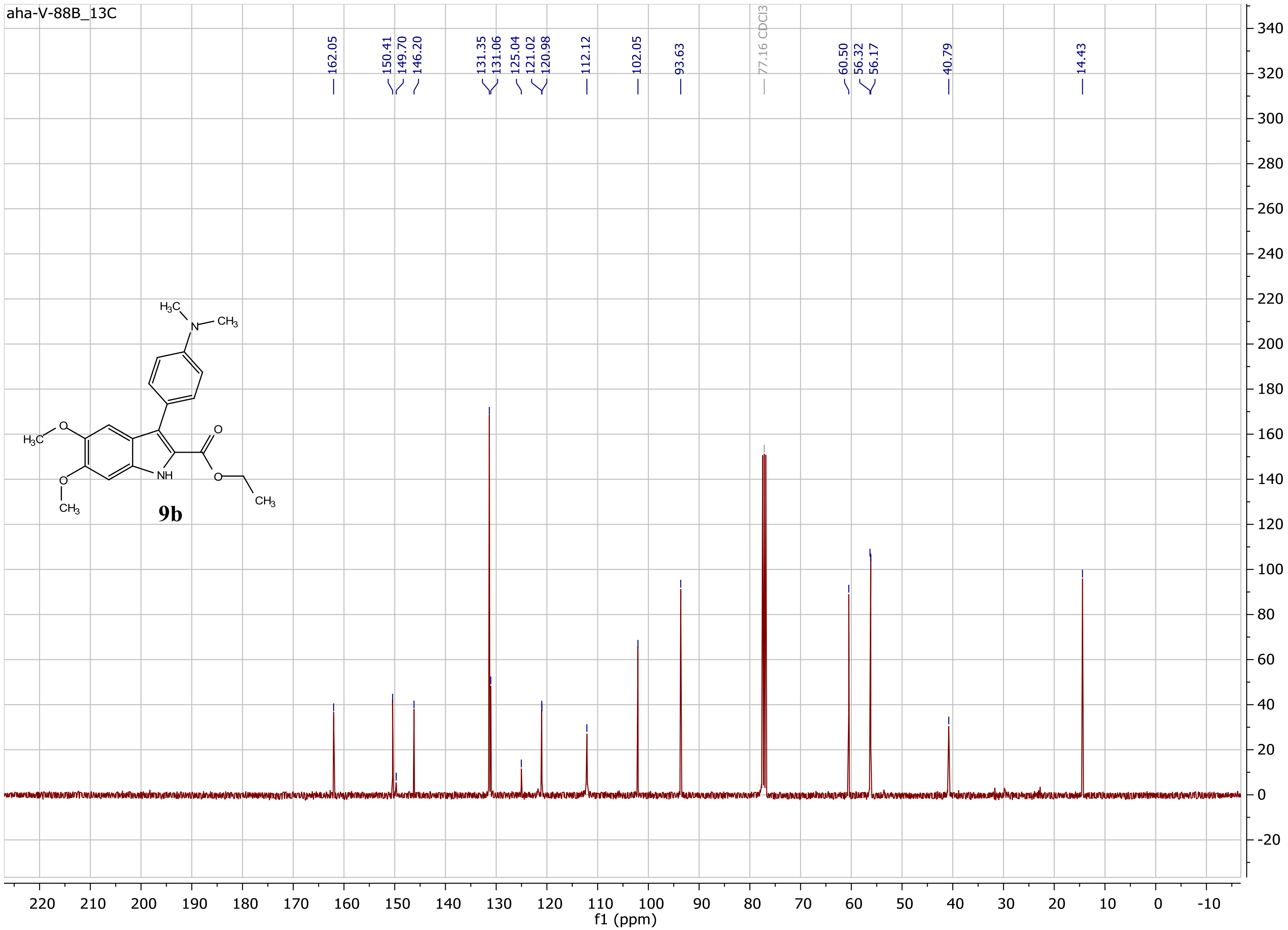
aha-V-88B_13C
162.05
150.41
149.70
146.20
131.35
131.06
125.04
121.02
120.98
112.12
102.05
93.63
77.16 CDCl3
60.50
56.32
56.17
40.79
14.43
9b
f1 (ppm)

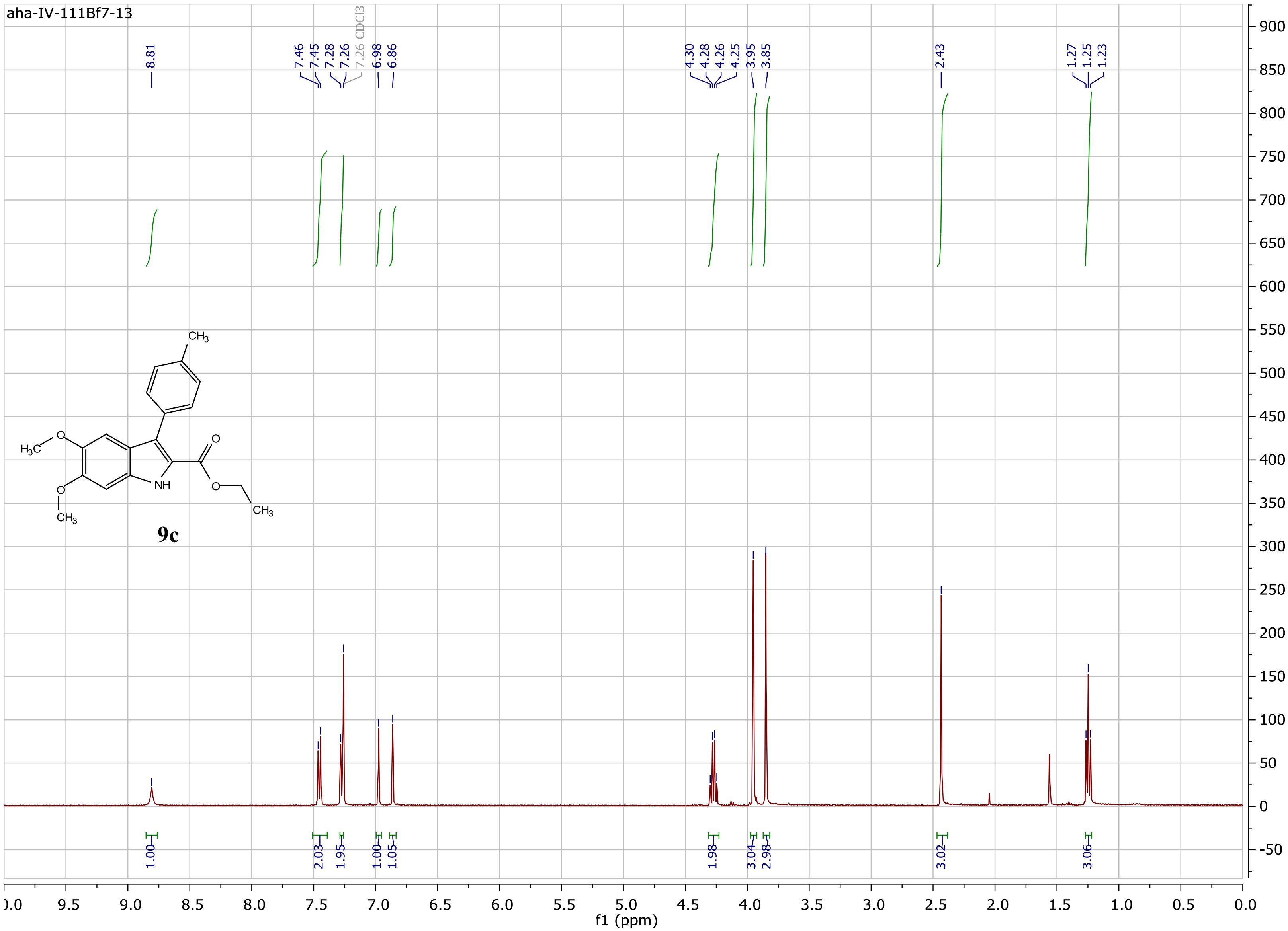
aha-IV-111Bf7-13
8.81
7.46
7.45
7.28
7.26
7.26 CDCl3
6.98
6.86
4.30
4.28
4.26
4.25
3.95
3.85
2.43
1.27
1.25
1.23
1.00
2.03
1.95
1.00
1.05
1.98
3.04
2.98
3.02
3.06
CH3
H3C
O
O
O
NH
O
CH3
CH3
9c
f1 (ppm)

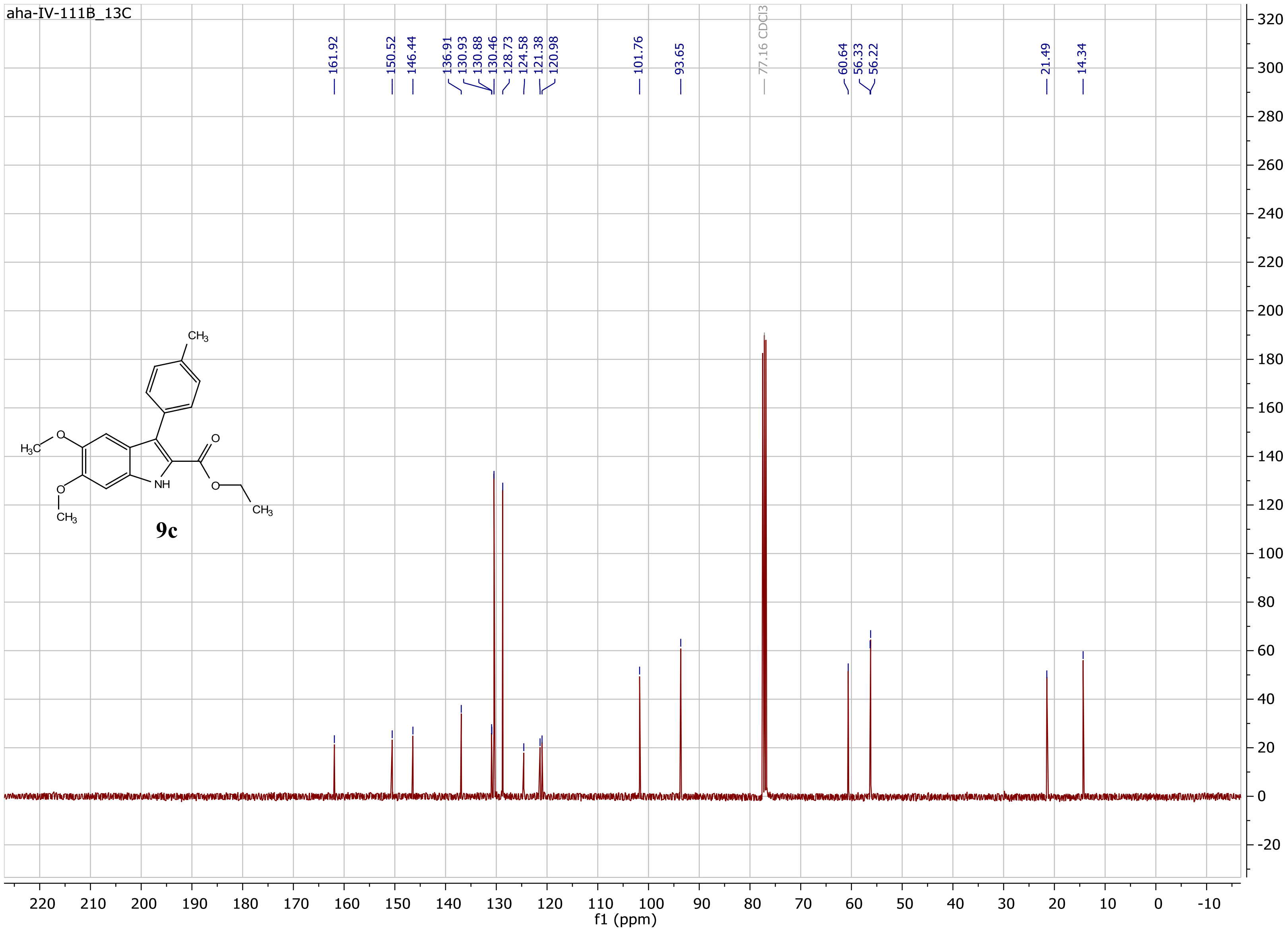

aha-IV-111B_13C
161.92
150.52
146.44
136.91
130.93
130.88
130.46
128.73
124.58
121.38
120.98
101.76
93.65
77.16 CDCl3
60.64
56.33
56.22
21.49
14.34
CH3
H3C
O
O
NH
O
O
CH3
CH3
9c
f1 (ppm)

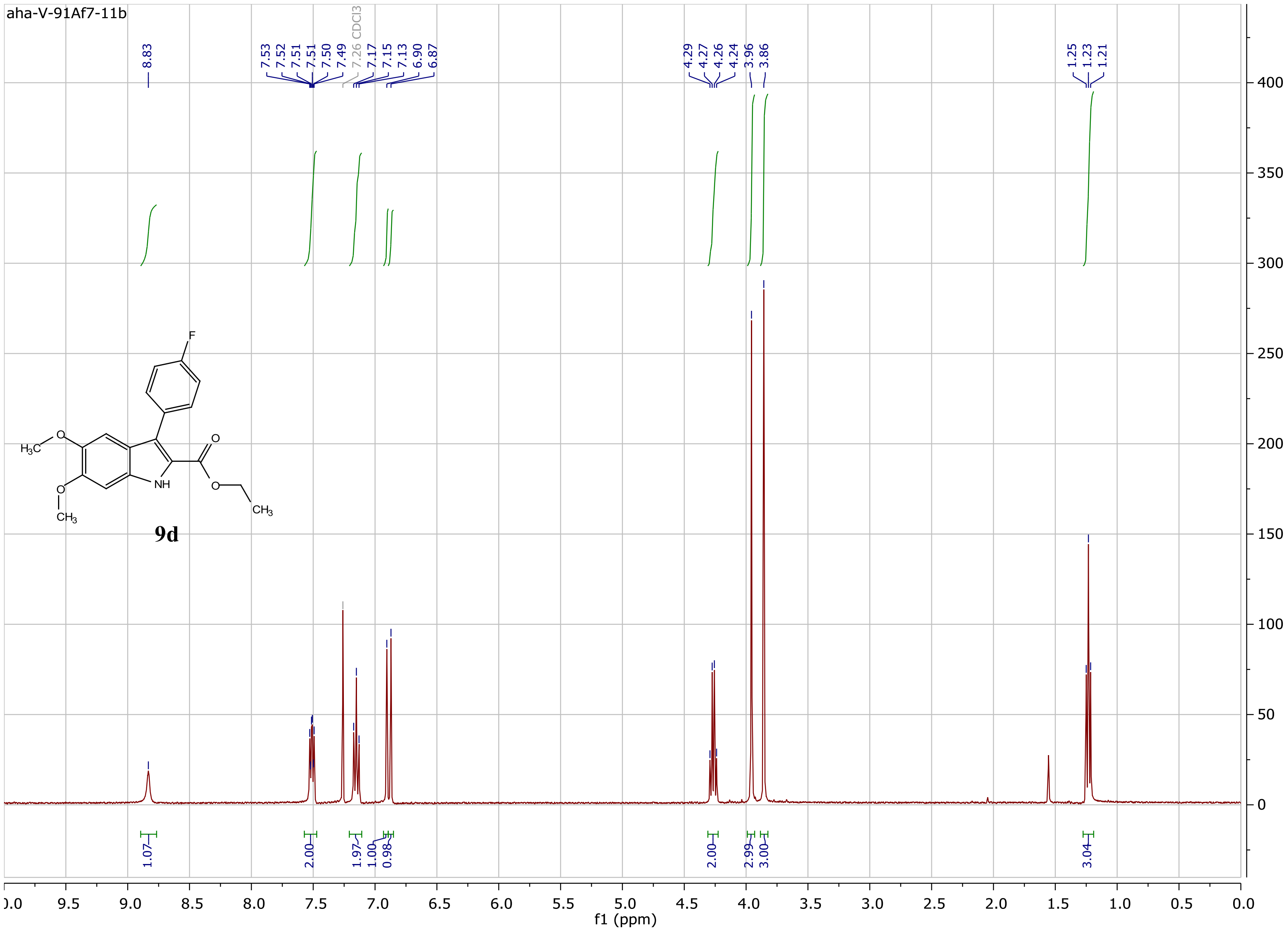
aha-V-91Af7-11b
8.83
7.53
7.52
7.51
7.51
7.50
7.49
7.26 CDCl3
7.17
7.15
7.13
6.90
6.87
4.29
4.27
4.26
4.24
3.96
3.86
1.25
1.23
1.21
1.07
2.00
1.97
1.00
0.98
2.00
2.99
3.00
3.04
f1 (ppm)
F
O
NH
CH3
9d

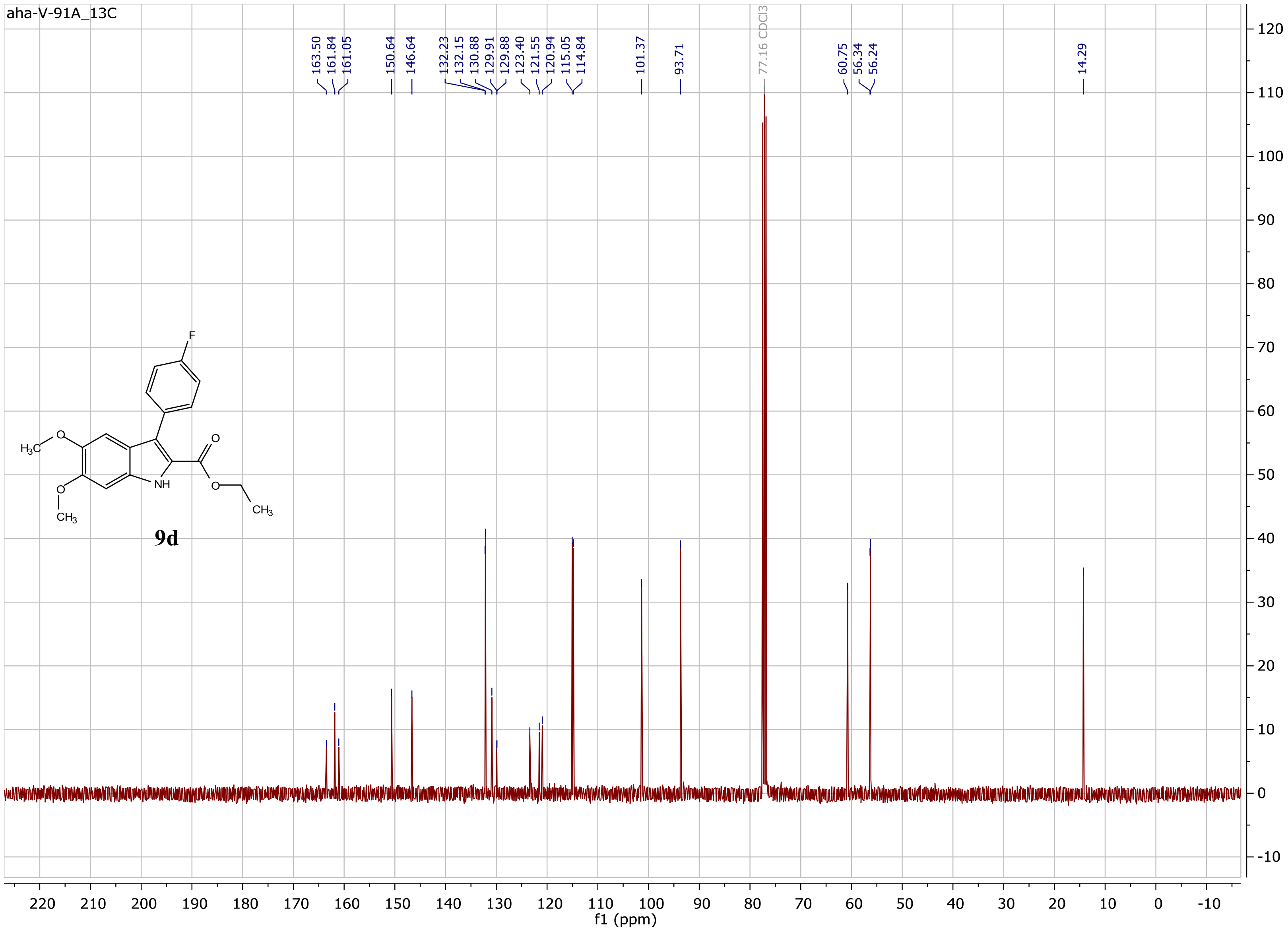
aha-V-91A_13C
163.50
161.84
161.05
150.64
146.64
132.23
132.15
130.88
129.91
129.88
123.40
121.55
120.94
115.05
114.84
101.37
93.71
77.16 CDCl3
60.75
56.34
56.24
14.29
f1 (ppm)

9d

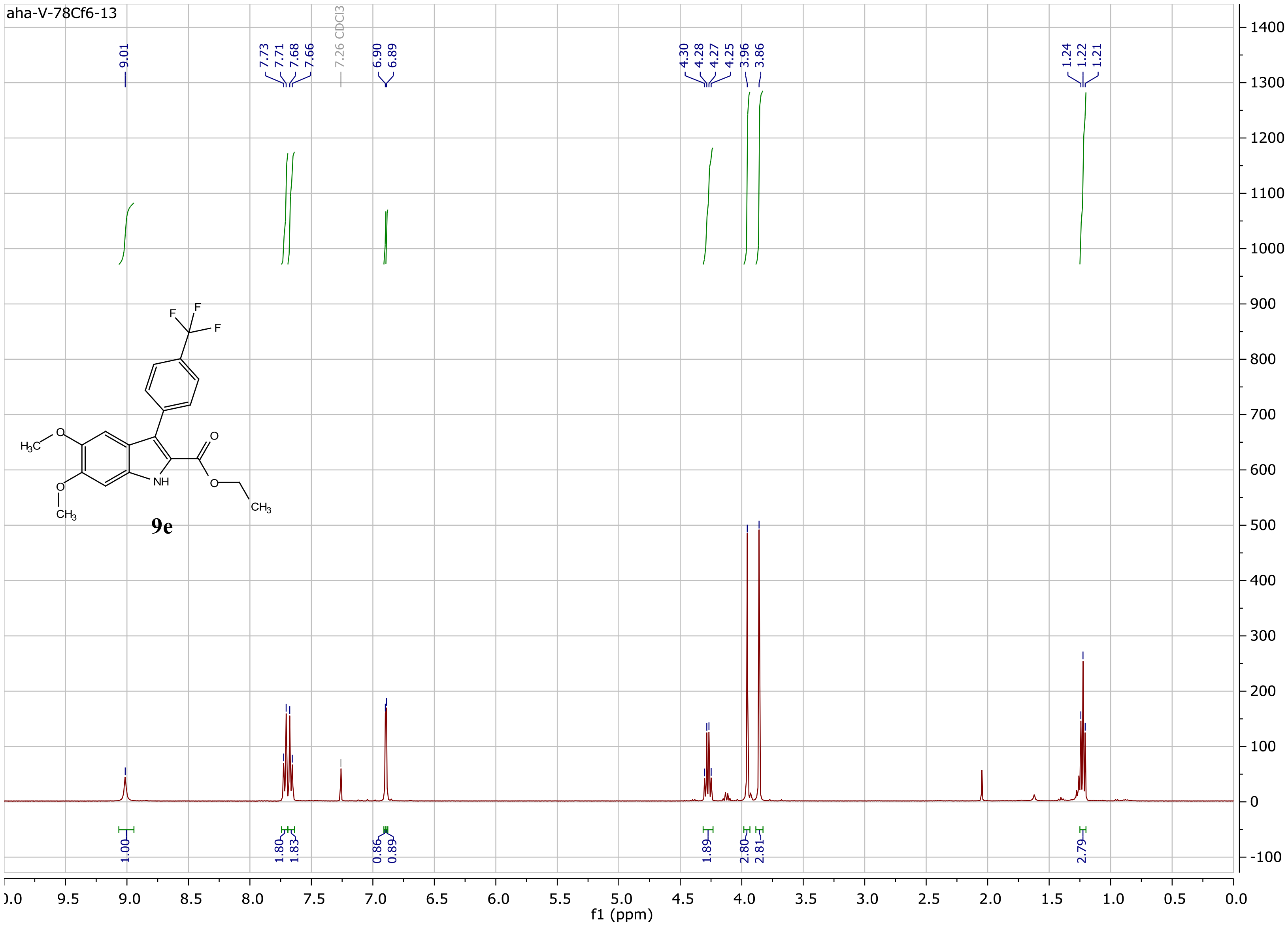

aha-V-78Cf6-13
9.01
7.73
7.71
7.68
7.66
7.26 CDCl3
6.90
6.89
4.30
4.28
4.27
4.25
3.96
3.86
1.24
1.22
1.21
1.00
1.80
1.83
0.86
0.89
1.89
2.80
2.81
2.79
f1 (ppm)
9e

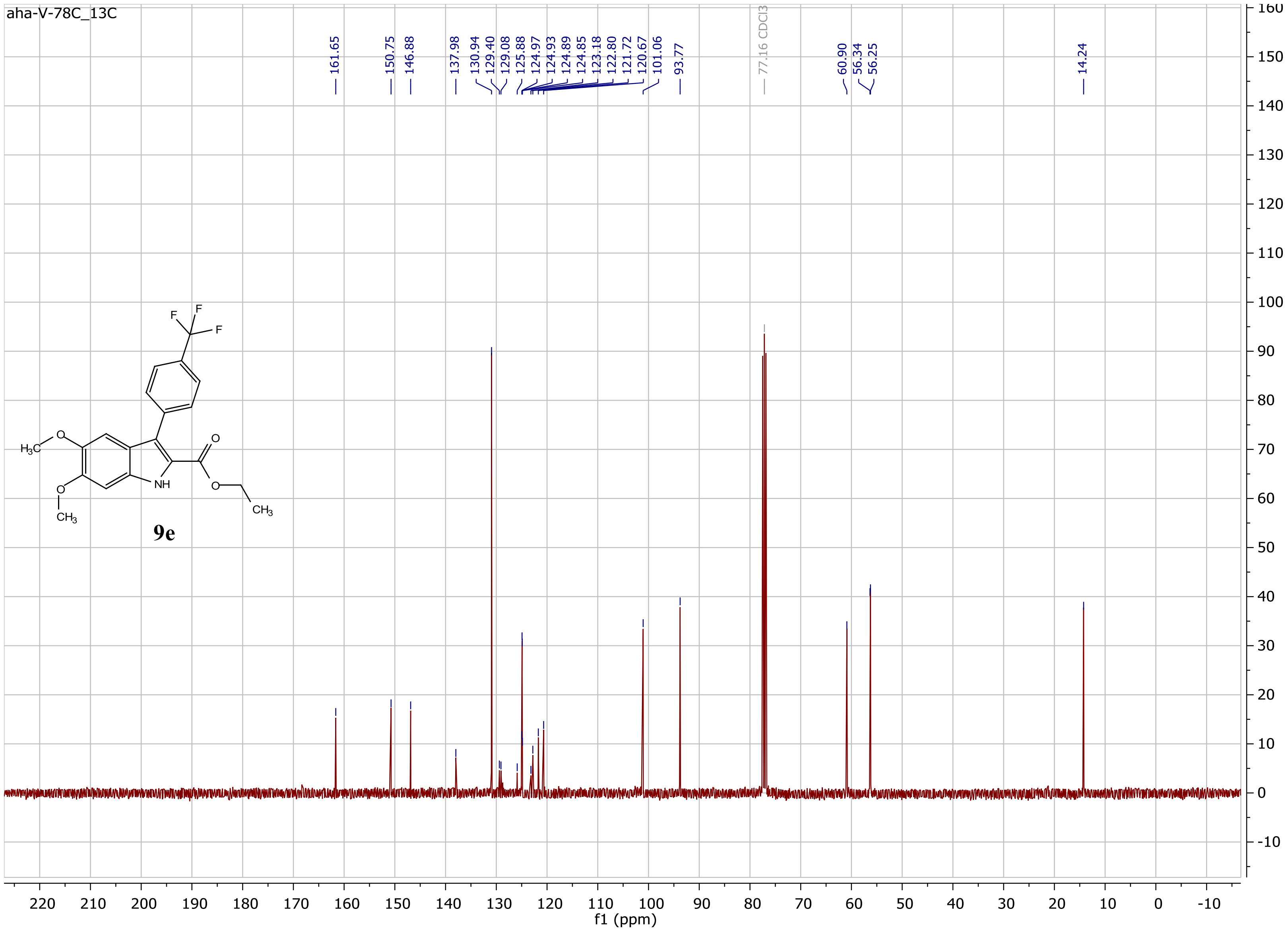

aha-V-78C_13C
161.65
150.75
146.88
137.98
130.94
129.40
129.08
125.88
124.97
124.93
124.89
124.85
123.18
122.80
121.72
120.67
101.06
93.77
77.16 CDCl3
60.90
56.34
56.25
14.24
9e
f1 (ppm)

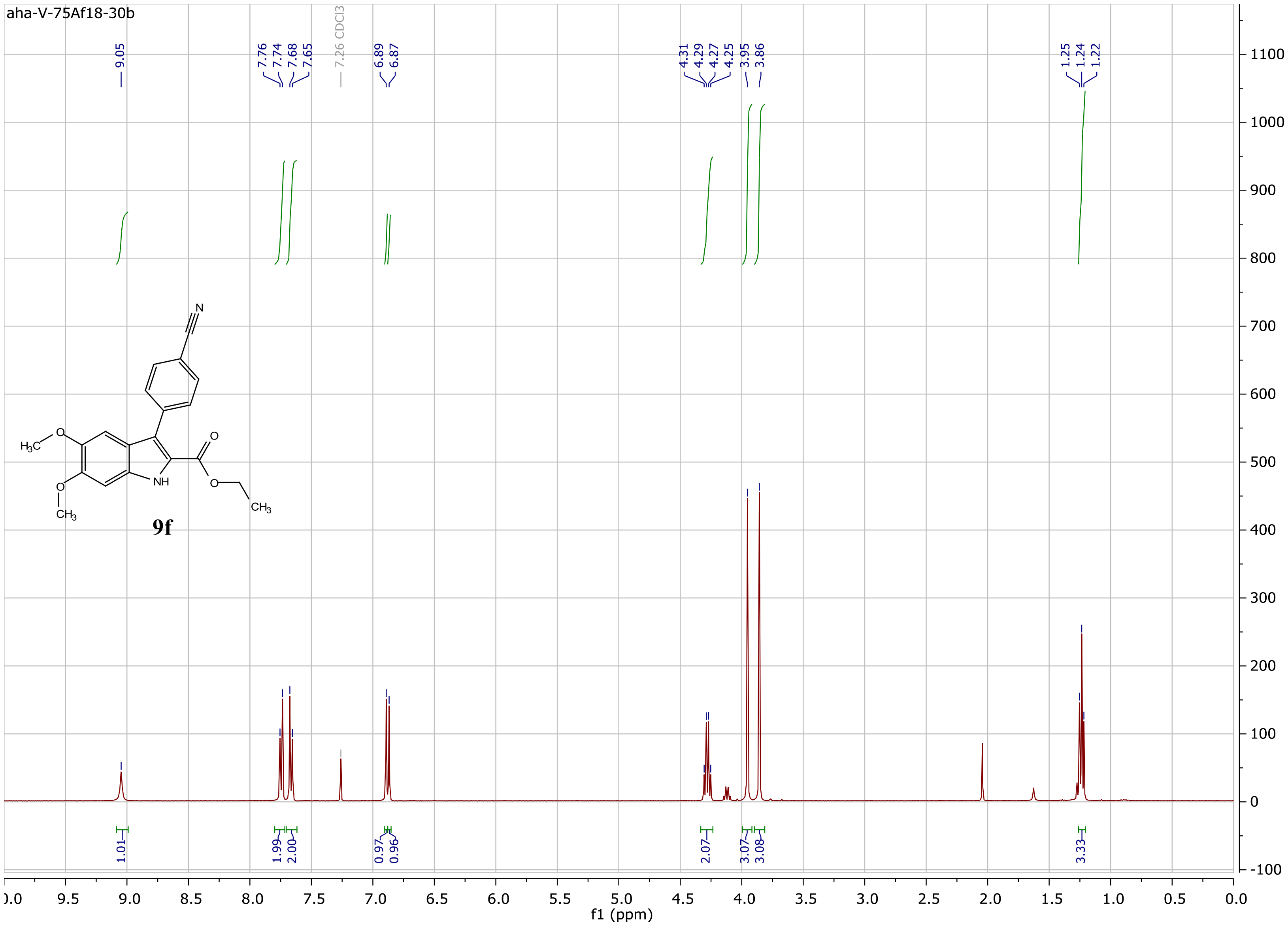
aha-V-75Af18-30b
9.05
7.76
7.74
7.68
7.65
7.26 CDCl3
6.89
6.87
4.31
4.29
4.27
4.25
3.95
3.86
1.25
1.24
1.22
1.01
1.99
2.00
0.97
0.96
2.07
3.07
3.08
3.33
f1 (ppm)
N
O
H3C
O
NH
O
O
CH3
CH3
9f

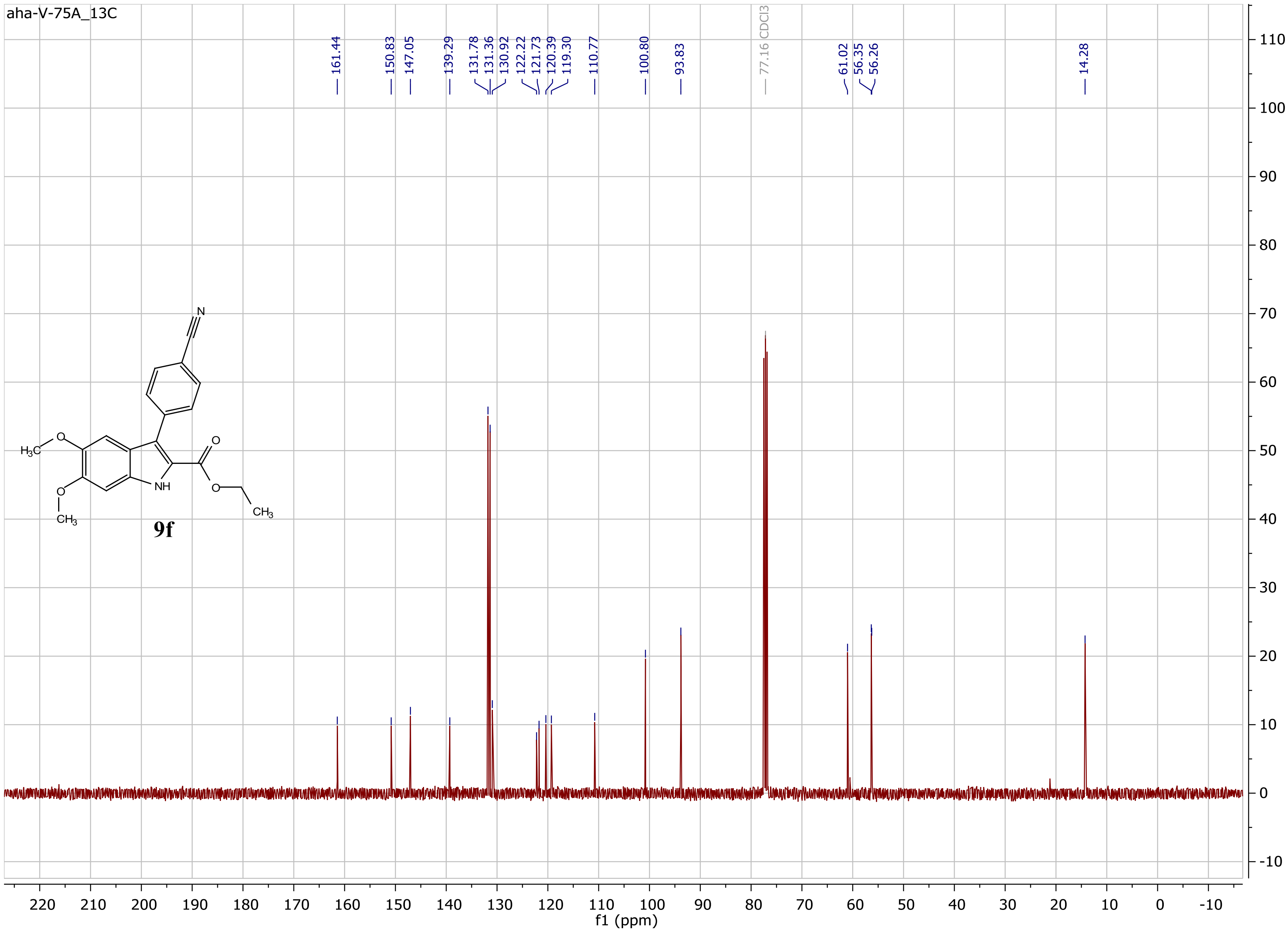
aha-V-75A_13C
161.44
150.83
147.05
139.29
131.78
131.36
130.92
122.22
121.73
120.39
119.30
110.77
100.80
93.83
77.16 CDCl3
61.02
56.35
56.26
14.28
N
O
H_3C
O
NH
O
CH_3
O
CH_3
9f
f1 (ppm)

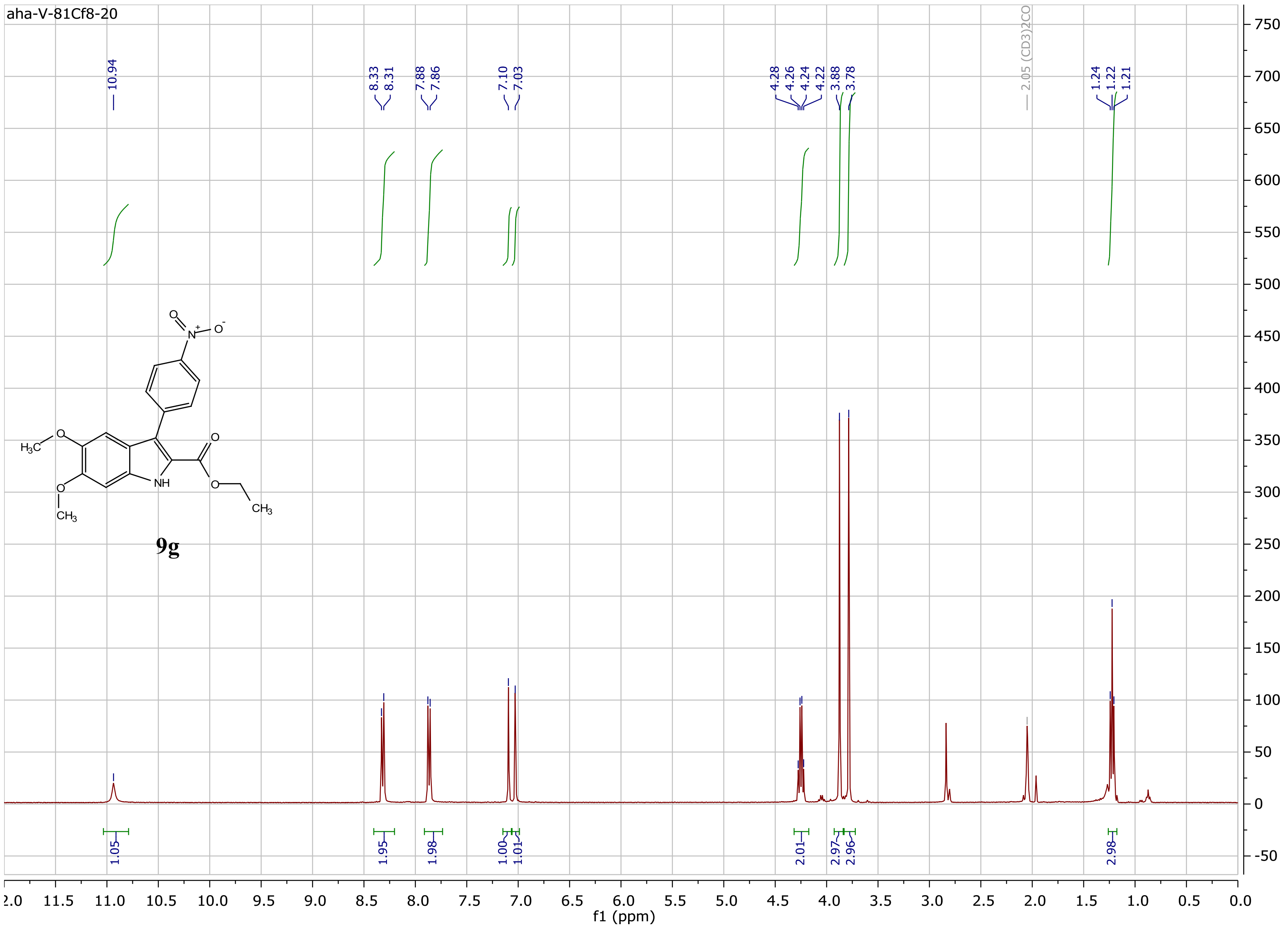

aha-V-81Cf8-20
10.94
8.33
8.31
7.88
7.86
7.10
7.03
4.28
4.26
4.24
4.22
3.88
3.78
2.05 (CD3)2CO
1.24
1.22
1.21
1.05
1.95
1.98
1.00
1.01
2.01
2.97
2.96
2.98
f1 (ppm)

9g

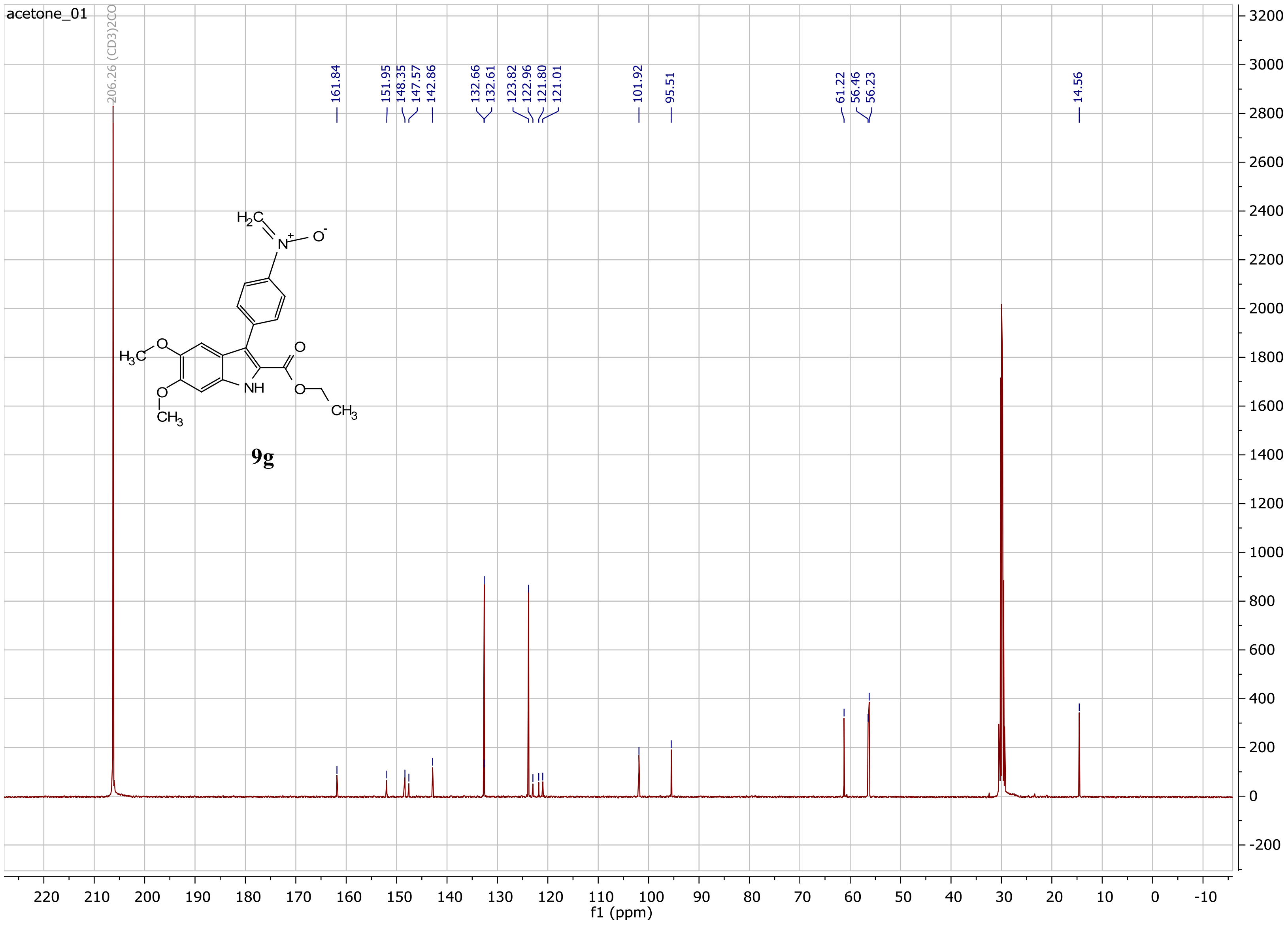

acetone_01
206.26 (CD3)2CO
161.84
151.95
148.35
147.57
142.86
132.66
132.61
123.82
122.96
121.80
121.01
101.92
95.51
61.22
56.46
56.23
14.56
f1 (ppm)
9g

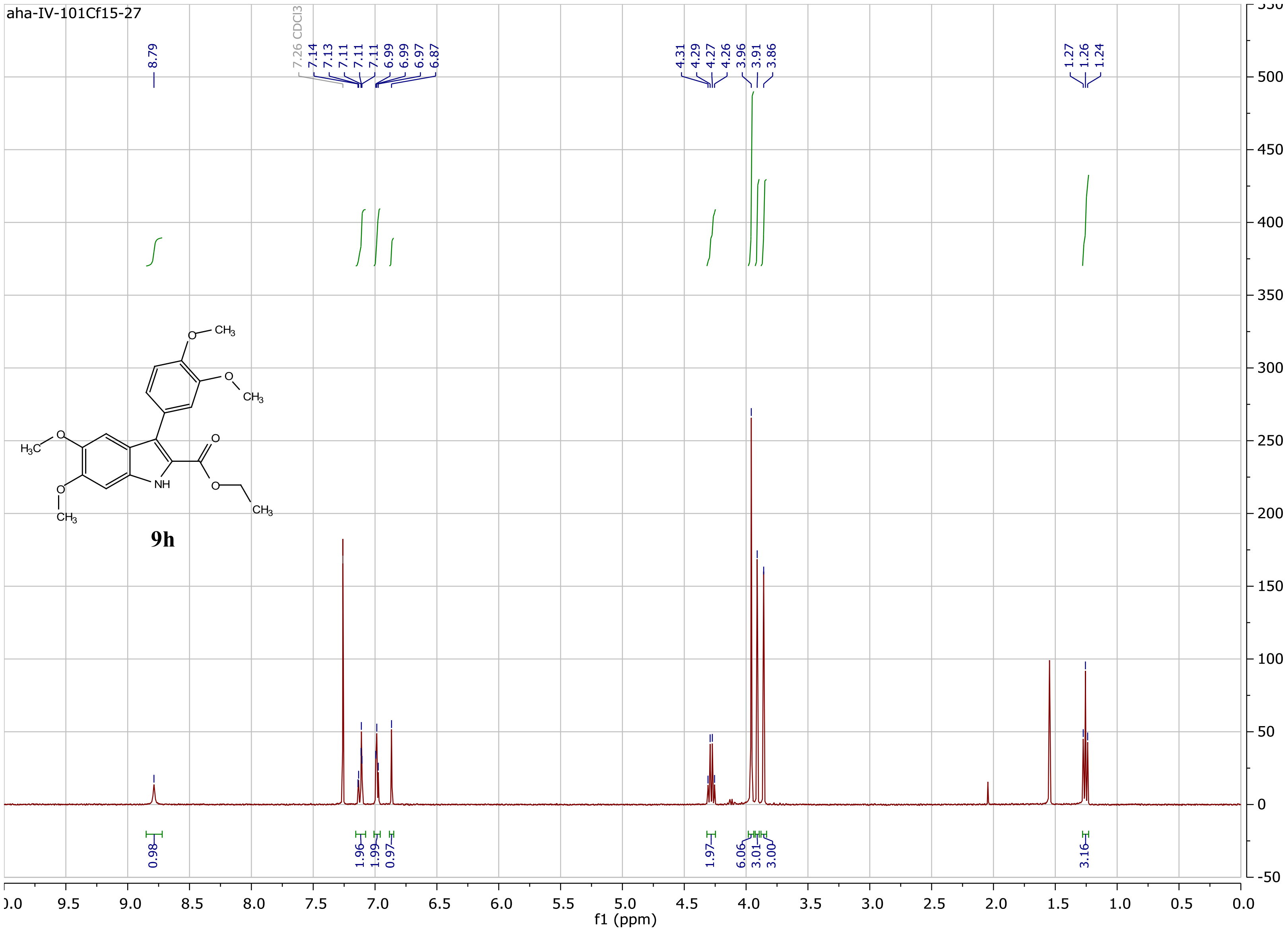
aha-IV-101Cf15-27
7.26 CDCl3
8.79
7.14
7.13
7.11
7.11
7.11
6.99
6.99
6.97
6.87
4.31
4.29
4.27
4.26
3.96
3.91
3.86
1.27
1.26
1.24
0.98
1.96
1.99
0.97
1.97
6.06
3.01
3.00
3.16
f1 (ppm)
9h

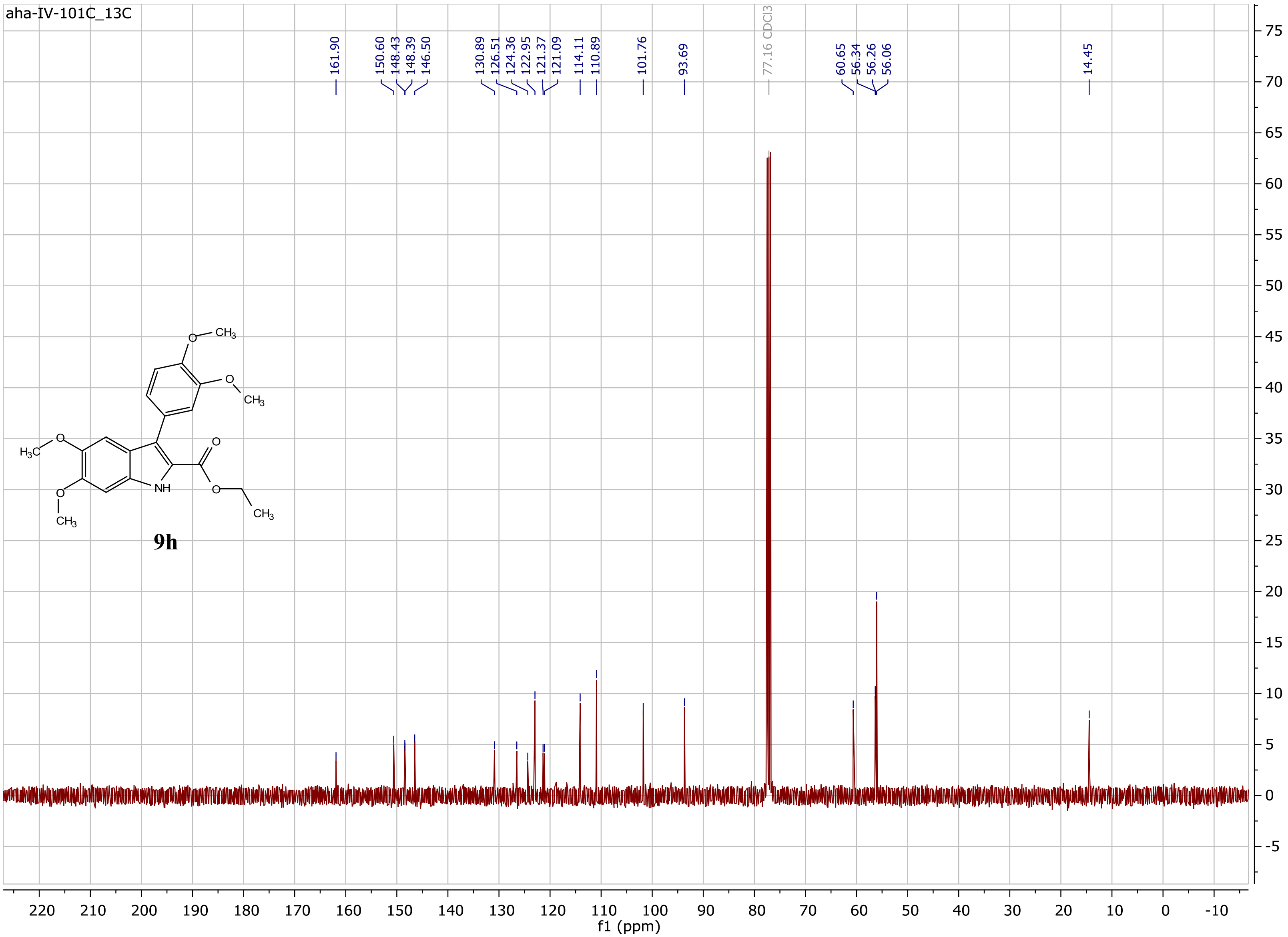

aha-IV-101C_13C
161.90
150.60
148.43
148.39
146.50
130.89
126.51
124.36
122.95
121.37
121.09
114.11
110.89
101.76
93.69
77.16 CDCl3
60.65
56.34
56.26
56.06
14.45
9h
f1 (ppm)

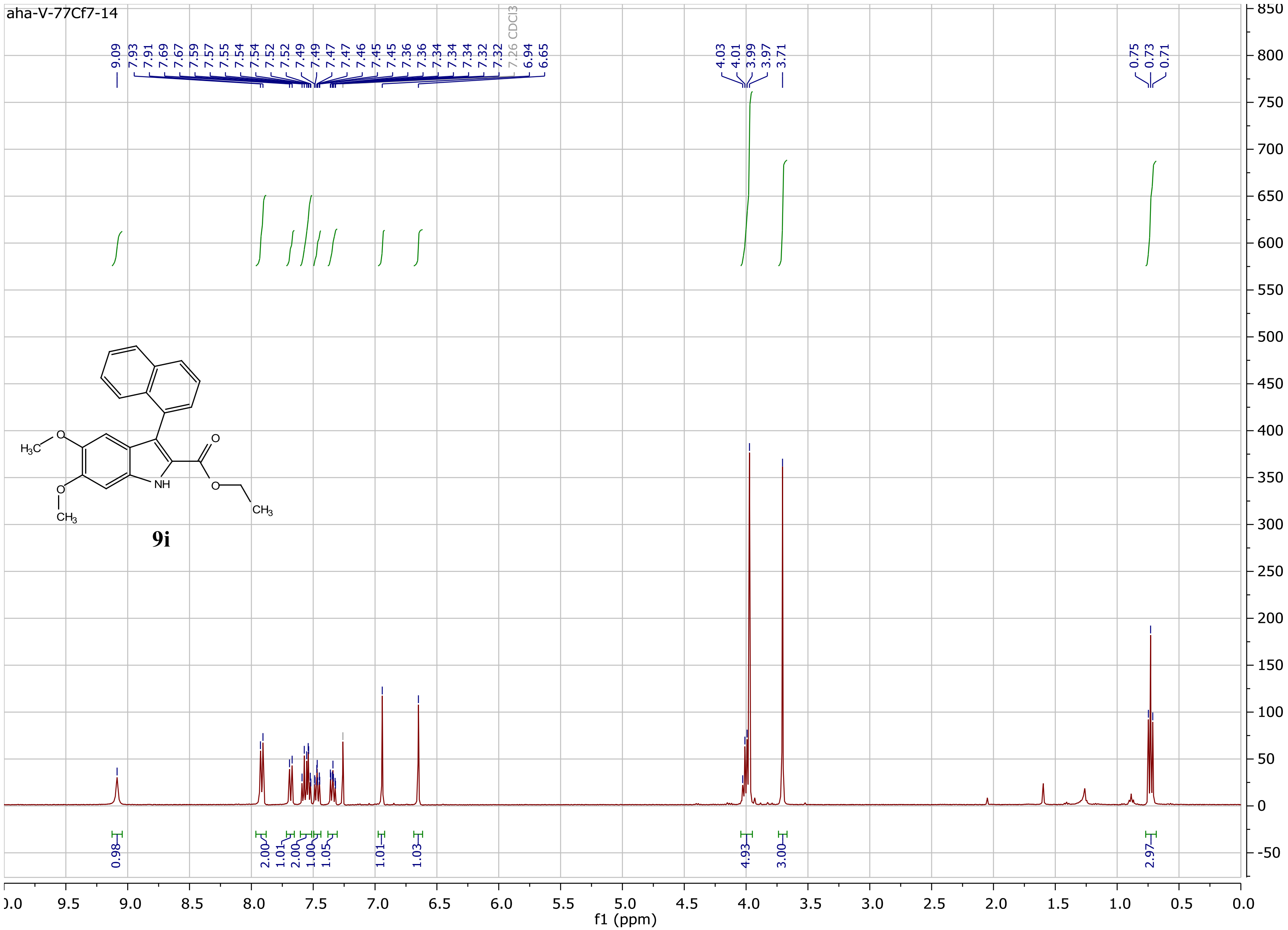
aha-V-77Cf7-14
9.09
7.93
7.91
7.69
7.67
7.59
7.57
7.55
7.54
7.54
7.52
7.52
7.49
7.47
7.47
7.46
7.45
7.45
7.36
7.36
7.34
7.34
7.34
7.32
7.32
7.26 CDCl3
6.94
6.65
4.03
4.01
3.99
3.97
3.71
0.75
0.73
0.71
9i
0.98
2.00
1.01
2.00
1.00
1.05
1.01
1.03
4.93
3.00
2.97
f1 (ppm)

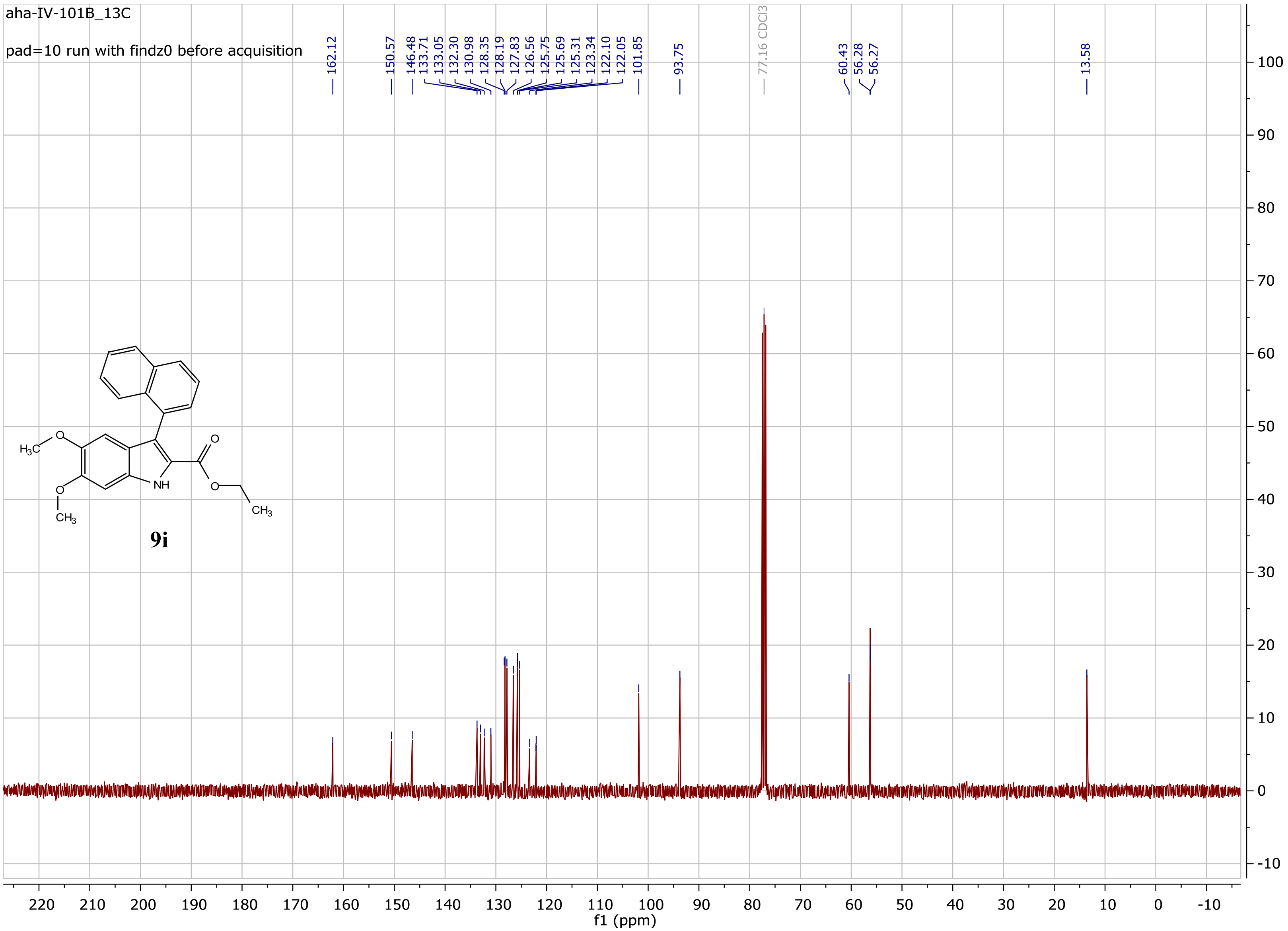

aha-IV-101B_13C
pad=10 run with findz0 before acquisition
162.12
150.57
146.48
133.71
133.05
132.30
130.98
128.35
128.19
127.83
126.56
125.75
125.69
125.31
123.34
122.10
122.05
101.85
93.75
77.16 CDCl3
60.43
56.28
56.27
13.58
9i
f1 (ppm)

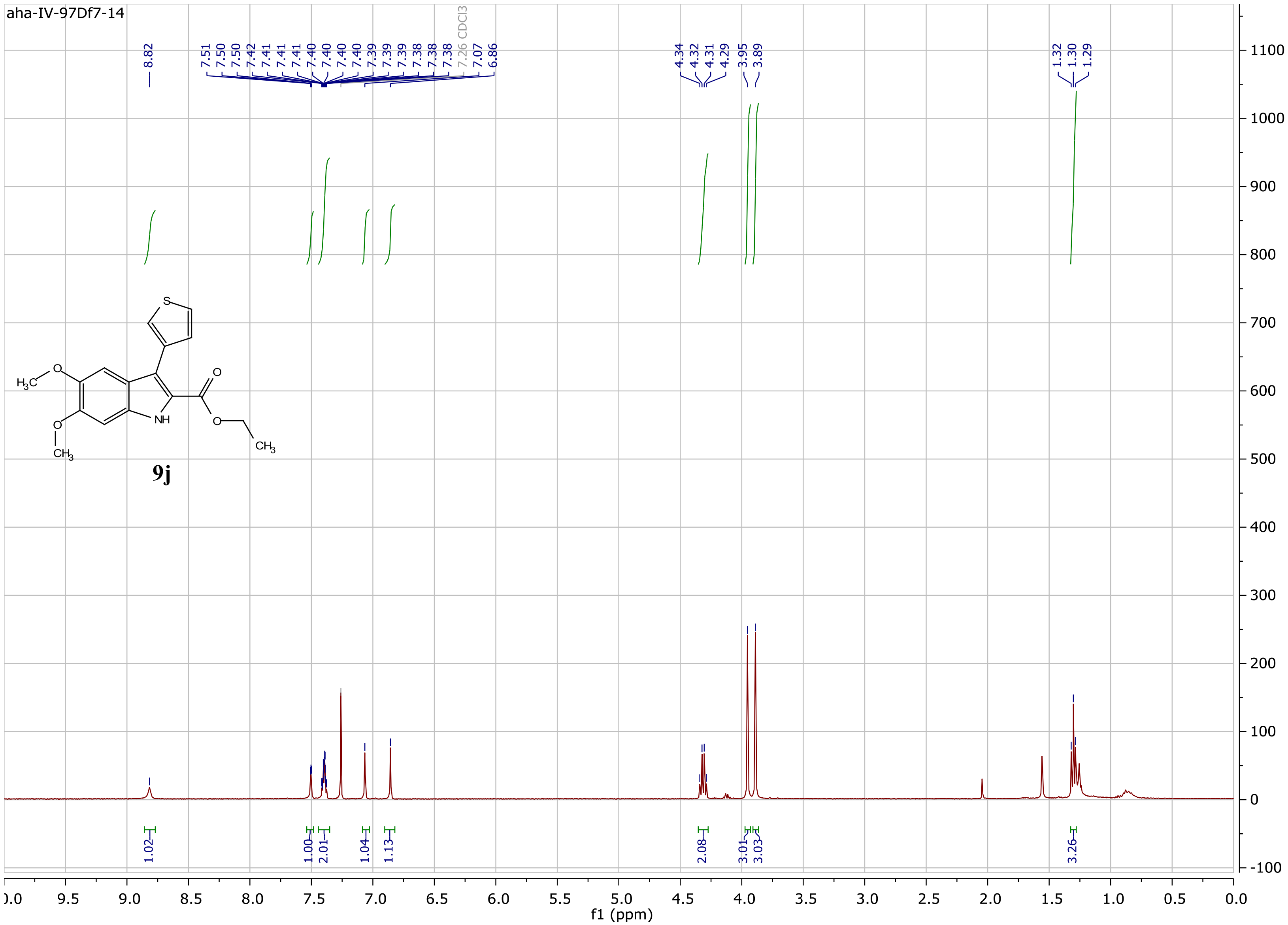
aha-IV-97Df7-14
8.82
7.51 7.50 7.50 7.42 7.41 7.41 7.41 7.40 7.40 7.40 7.39 7.39 7.39 7.38 7.38 7.38
7.26 CDCl3
7.07 6.86
4.34 4.32 4.31 4.29
3.95 3.89
1.32 1.30 1.29
1.02 1.00 2.01 1.04 1.13 2.08 3.01 3.03 3.26
f1 (ppm)
9j

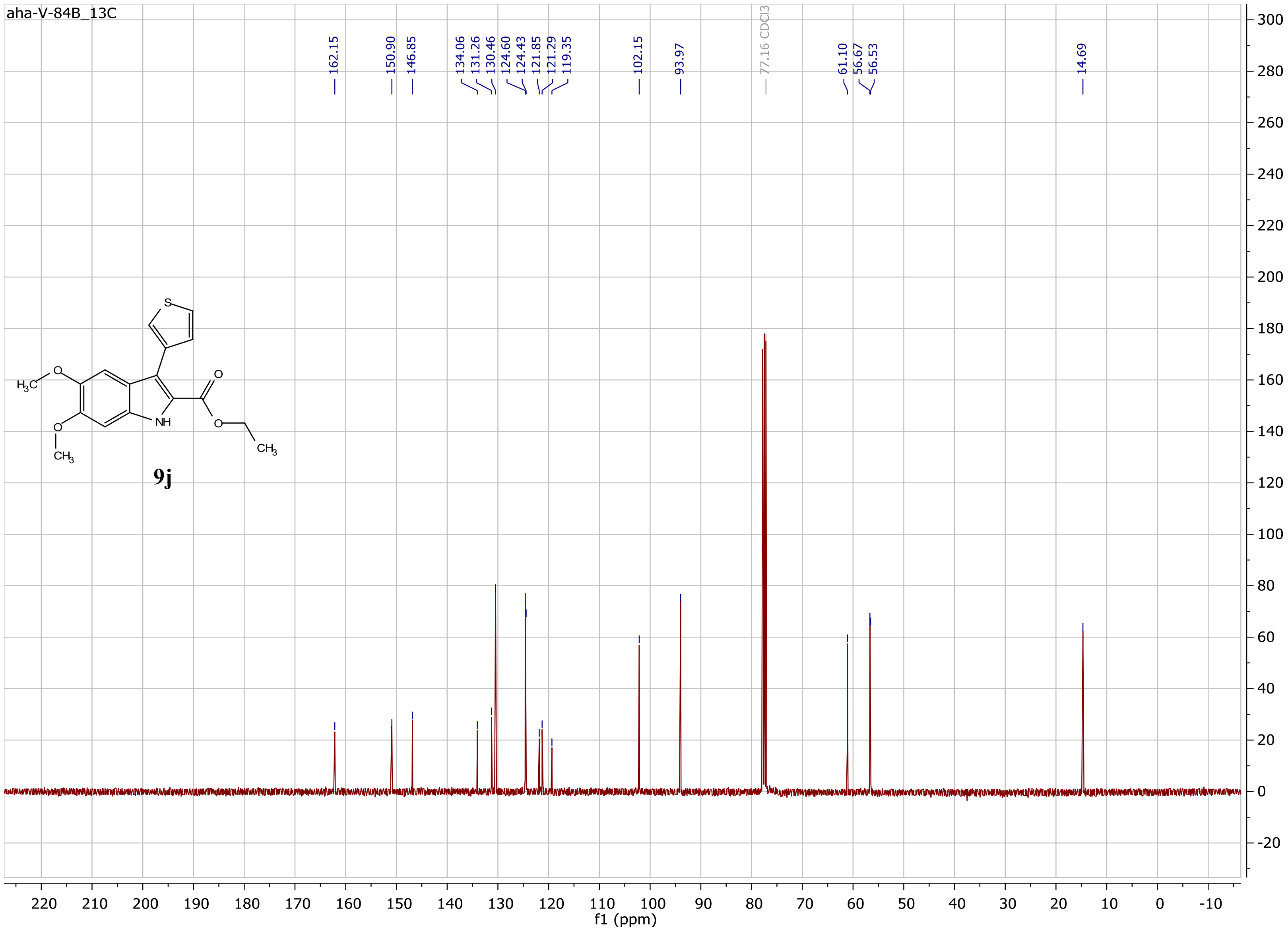
aha-V-84B_13C
162.15
150.90
146.85
134.06
131.26
130.46
124.60
124.43
121.85
121.29
119.35
102.15
93.97
77.16 CDCl3
61.10
56.67
56.53
14.69
9j
f1 (ppm)

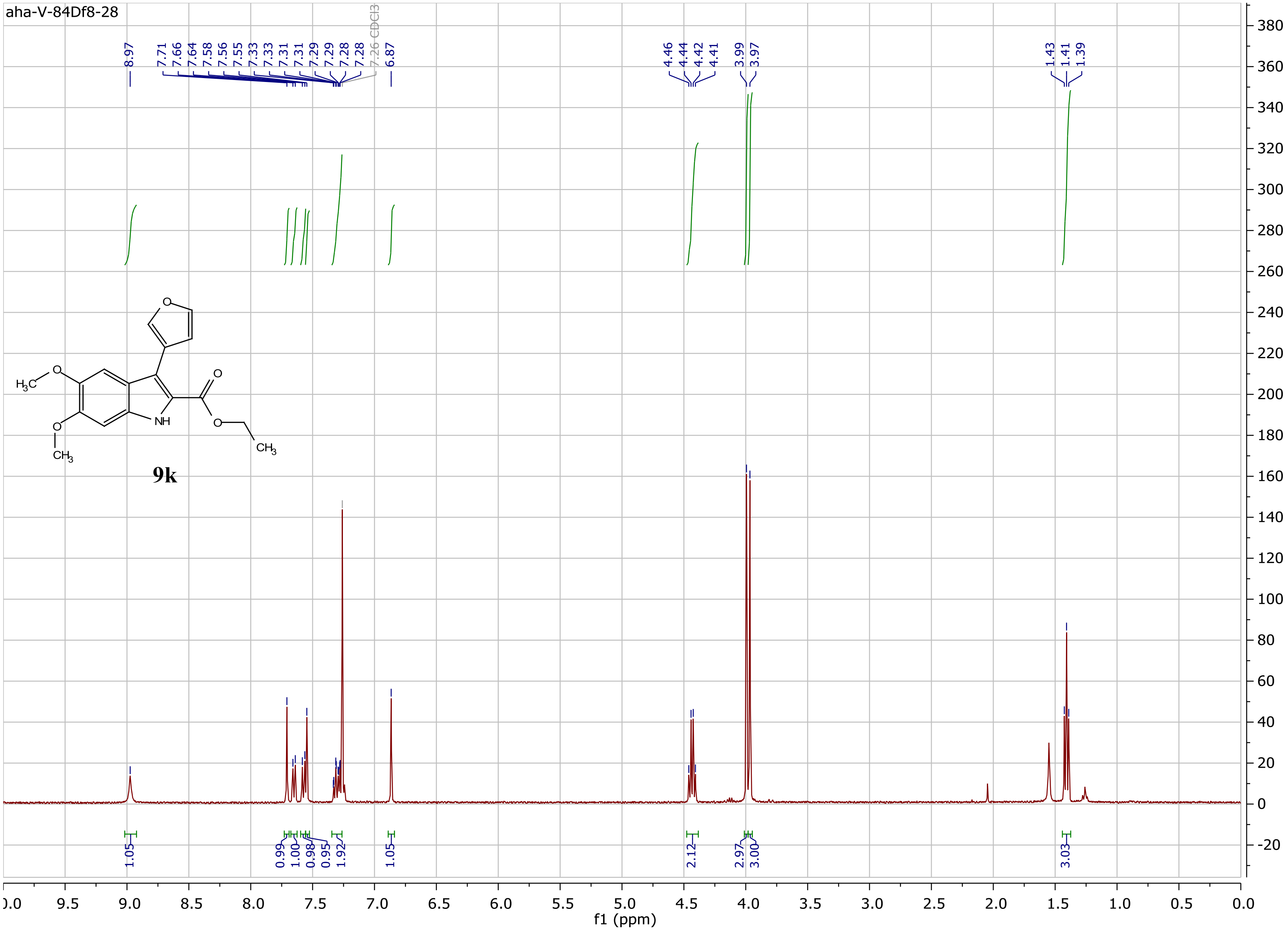

9k

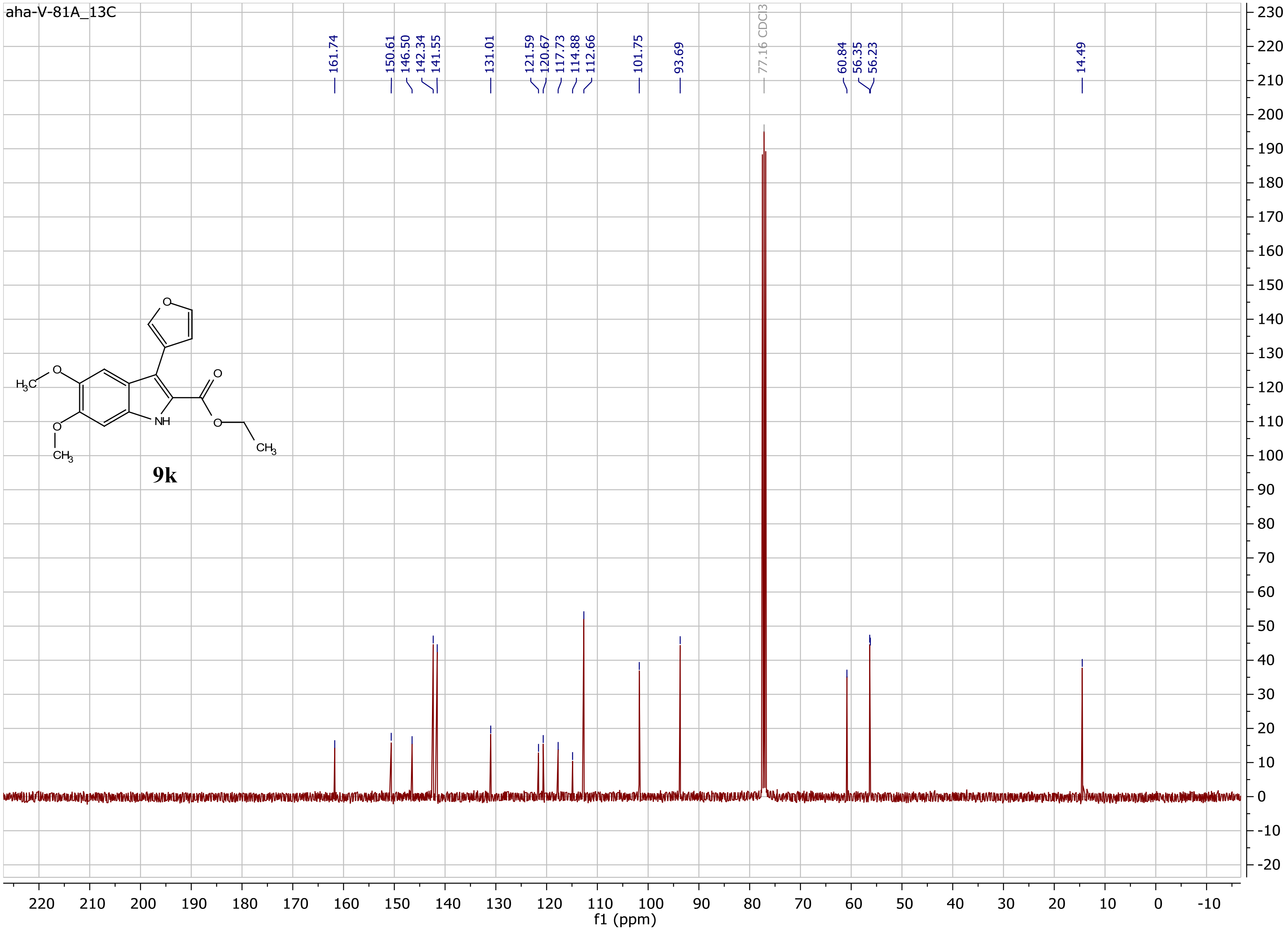
aha-V-81A_13C
161.74
150.61
146.50
142.34
141.55
131.01
121.59
120.67
117.73
114.88
112.66
101.75
93.69
77.16 CDCl3
60.84
56.35
56.23
14.49
f1 (ppm)
9k

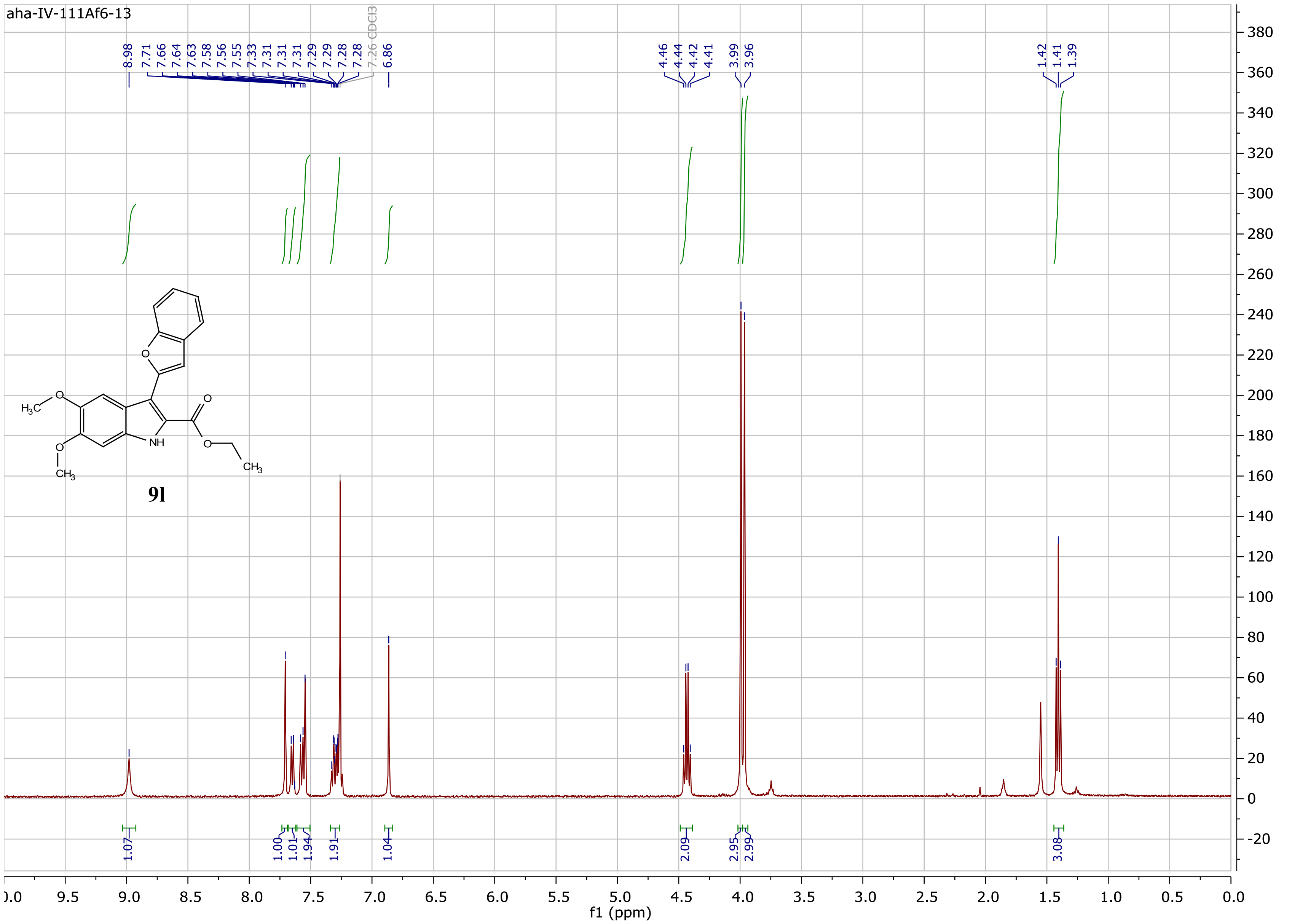
aha-IV-111Af6-13
8.98
7.71
7.66
7.64
7.63
7.58
7.56
7.55
7.33
7.31
7.31
7.31
7.29
7.29
7.28
7.28
7.26 CDCl3
6.86
4.46
4.44
4.42
4.41
3.99
3.96
1.42
1.41
1.39
O
O
H3C
O
NH
O
O
CH3
CH3
9l
1.07
1.00
1.01
1.94
1.91
1.04
2.09
2.95
2.99
3.08
f1 (ppm)

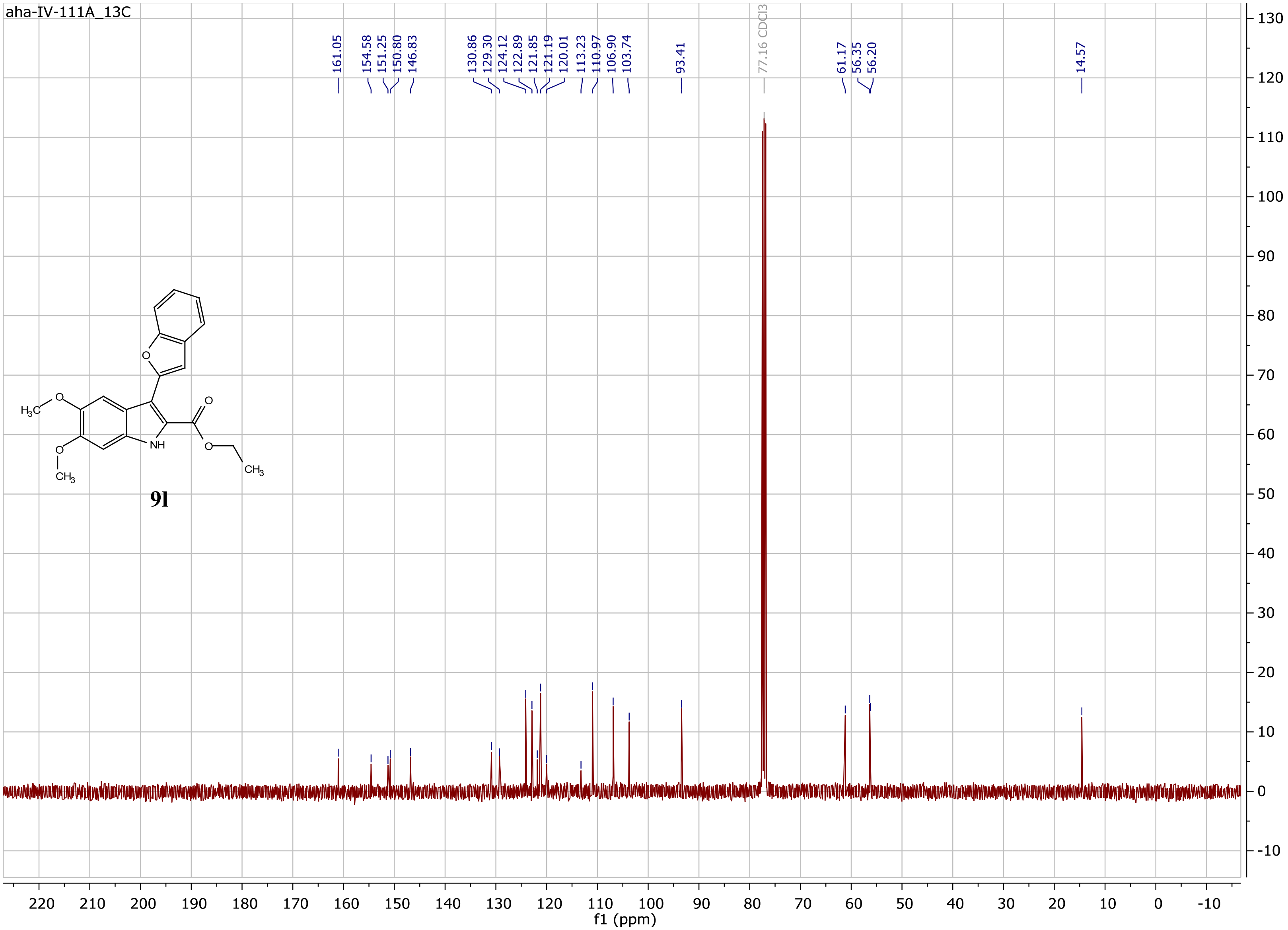

aha-IV-111A_13C
161.05
154.58
151.25
150.80
146.83
130.86
129.30
124.12
122.89
121.85
121.19
120.01
113.23
110.97
106.90
103.74
93.41
77.16 CDCl3
61.17
56.35
56.20
14.57
9l
f1 (ppm)

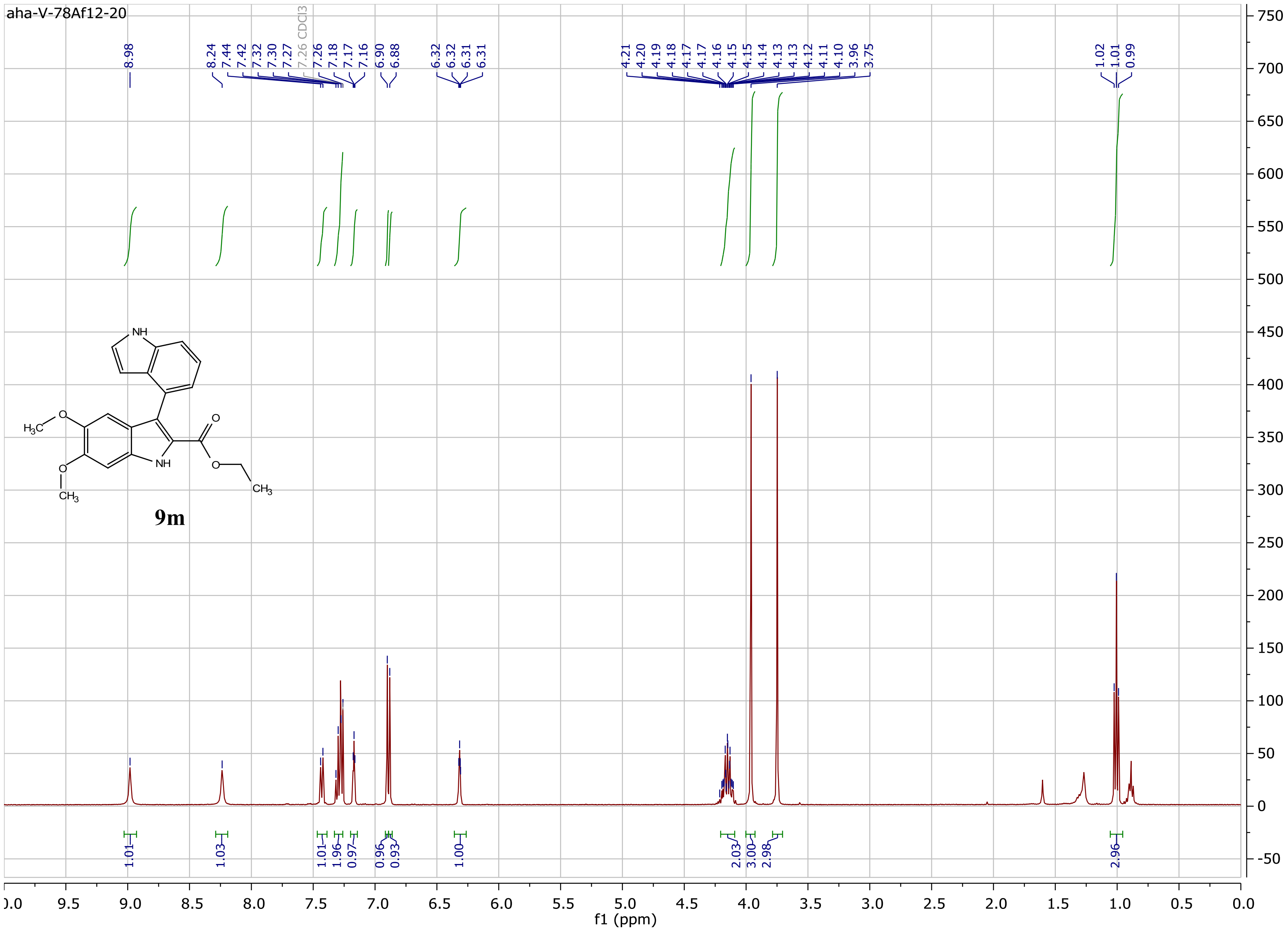
aha-V-78Af12-20
9m
f1 (ppm)

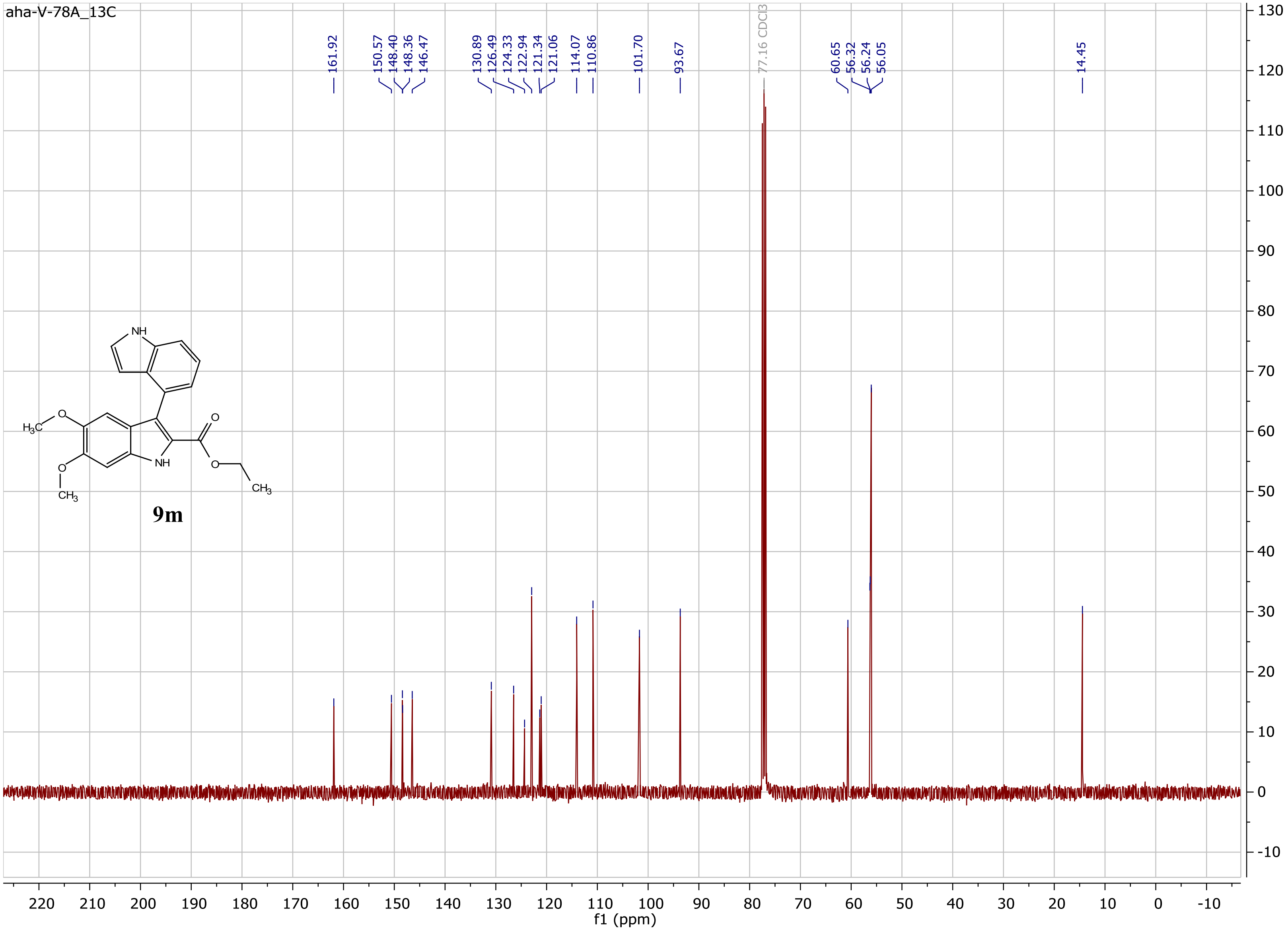
aha-V-78A_13C
161.92
150.57
148.40
148.36
146.47
130.89
126.49
124.33
122.94
121.34
121.06
114.07
110.86
101.70
93.67
77.16 CDCl3
60.65
56.32
56.24
56.05
14.45
f1 (ppm)

9m

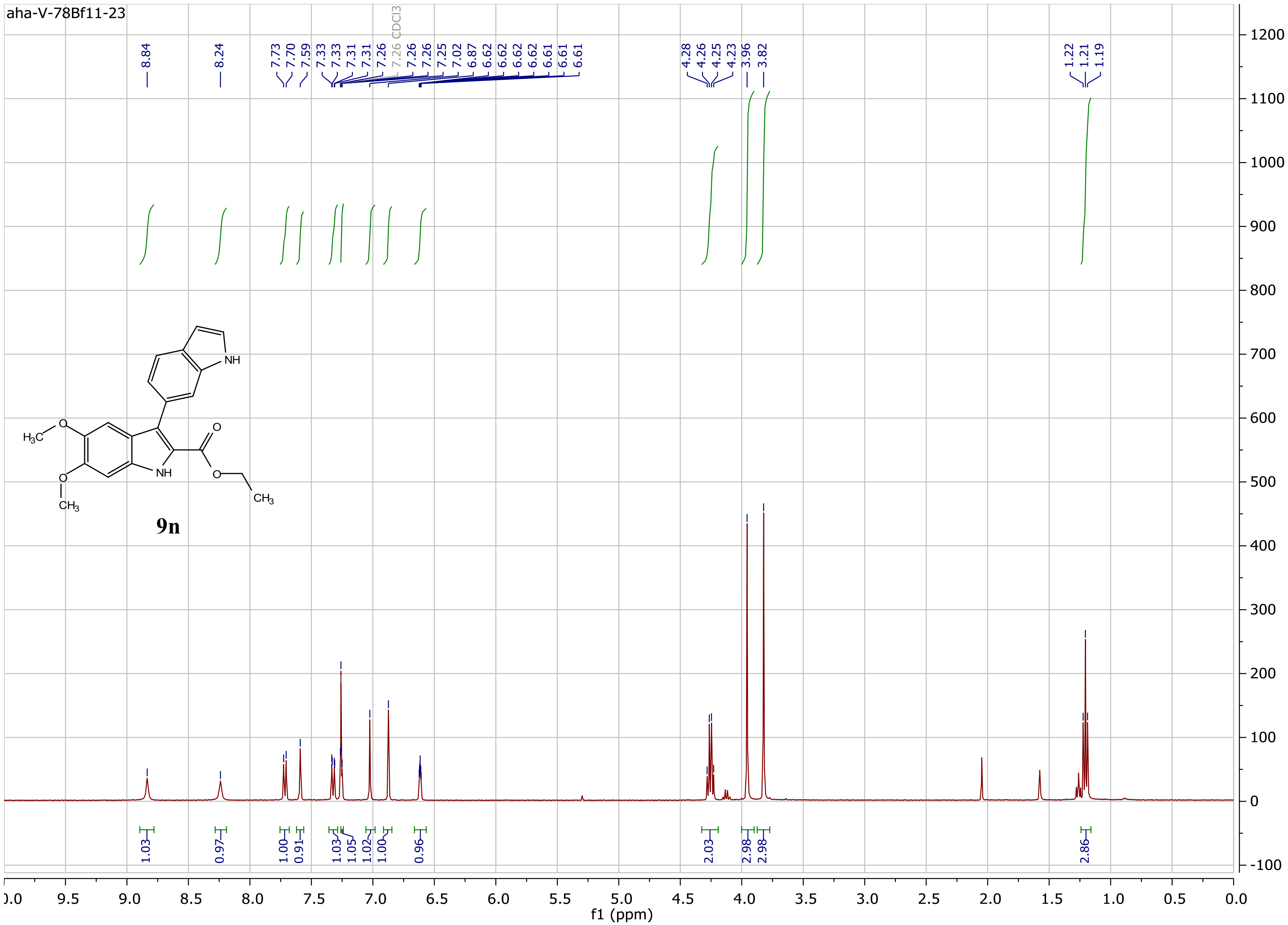
aha-V-78Bf11-23
8.84
8.24
7.73 7.70 7.59 7.33 7.33 7.31 7.31 7.26 7.26 CDCl3 7.26 7.26 7.25 7.02 6.87 6.62 6.62 6.62 6.62 6.61 6.61 6.61
4.28 4.26 4.25 4.23 3.96 3.82
1.22 1.21 1.19
NH
H_3C
O
O
O
NH
O
CH_3
CH_3
9n
1.03 0.97 1.00 0.91 1.03 1.05 1.02 1.00 0.96 2.03 2.98 2.98 2.86
f1 (ppm)

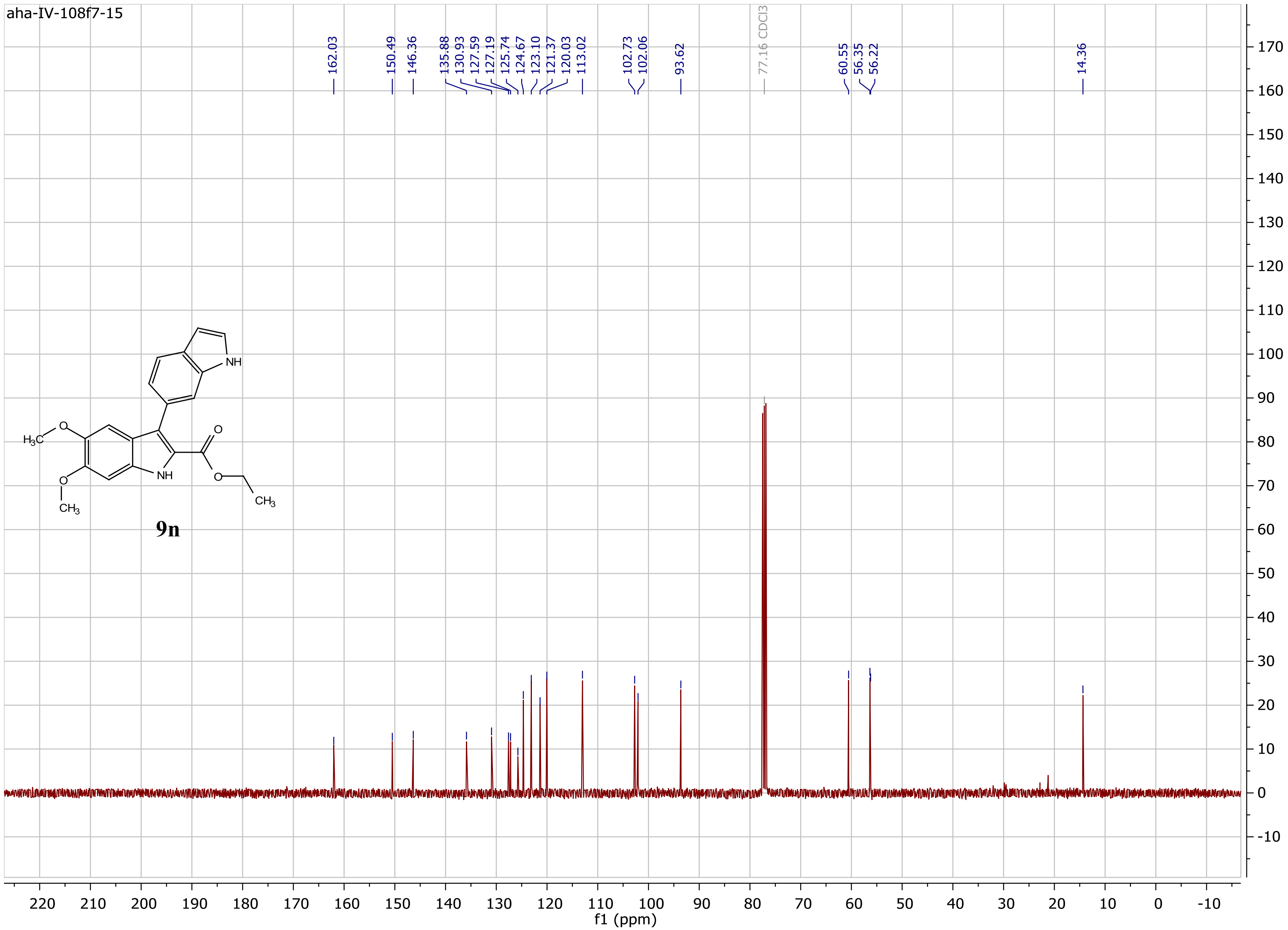
aha-IV-108f7-15
162.03
150.49
146.36
135.88
130.93
127.59
127.19
125.74
124.67
123.10
121.37
120.03
113.02
102.73
102.06
93.62
77.16 CDCl3
60.55
56.35
56.22
14.36
9n
f1 (ppm)

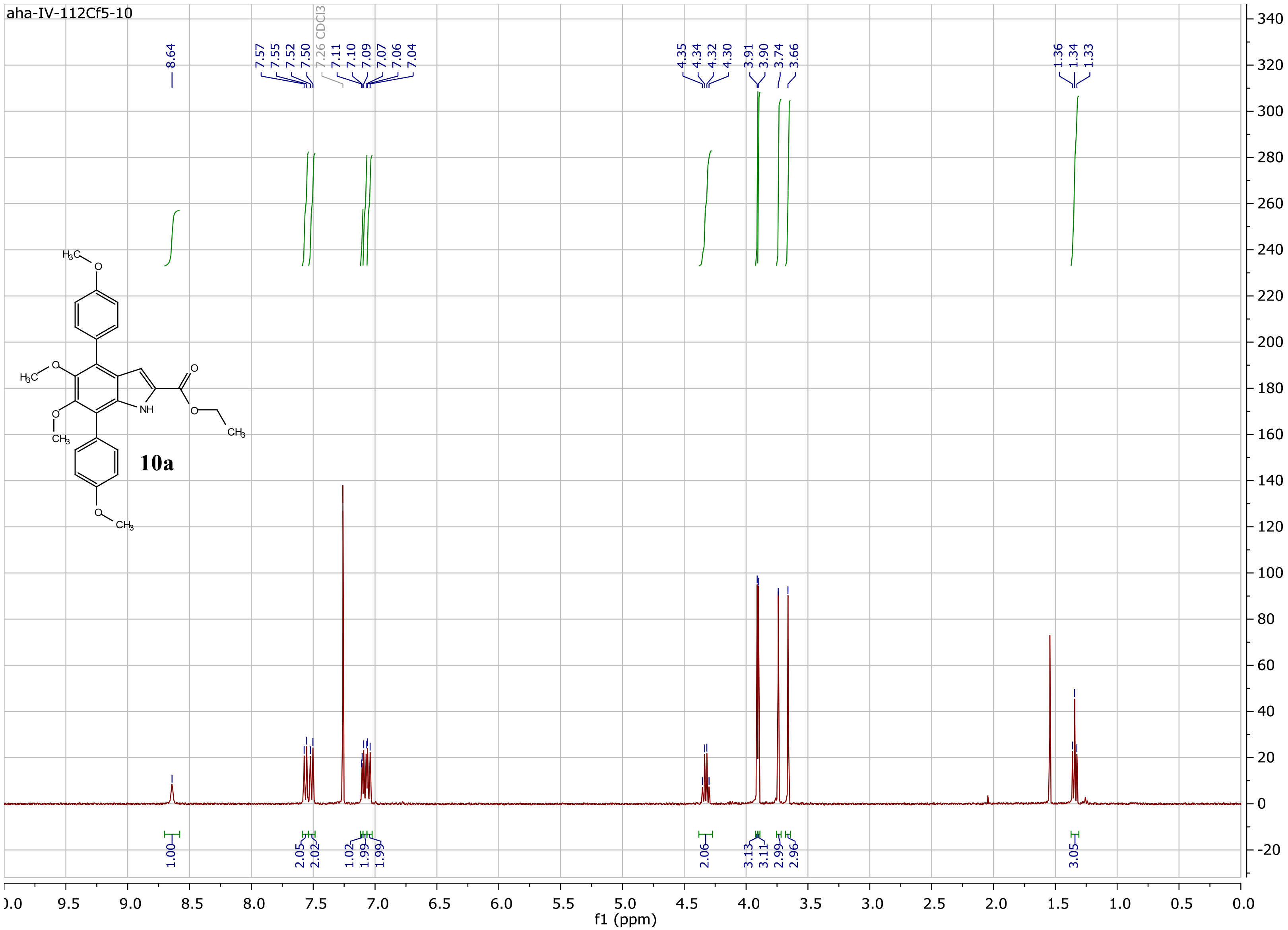

aha-IV-112Cf5-10
8.64
7.57
7.55
7.52
7.50
7.26 CDCl3
7.11
7.10
7.09
7.07
7.06
7.04
4.35
4.34
4.32
4.30
3.91
3.90
3.74
3.66
1.36
1.34
1.33
10a
1.00
2.05
2.02
1.02
1.99
1.99
2.06
3.13
3.11
2.99
2.96
3.05
f1 (ppm)

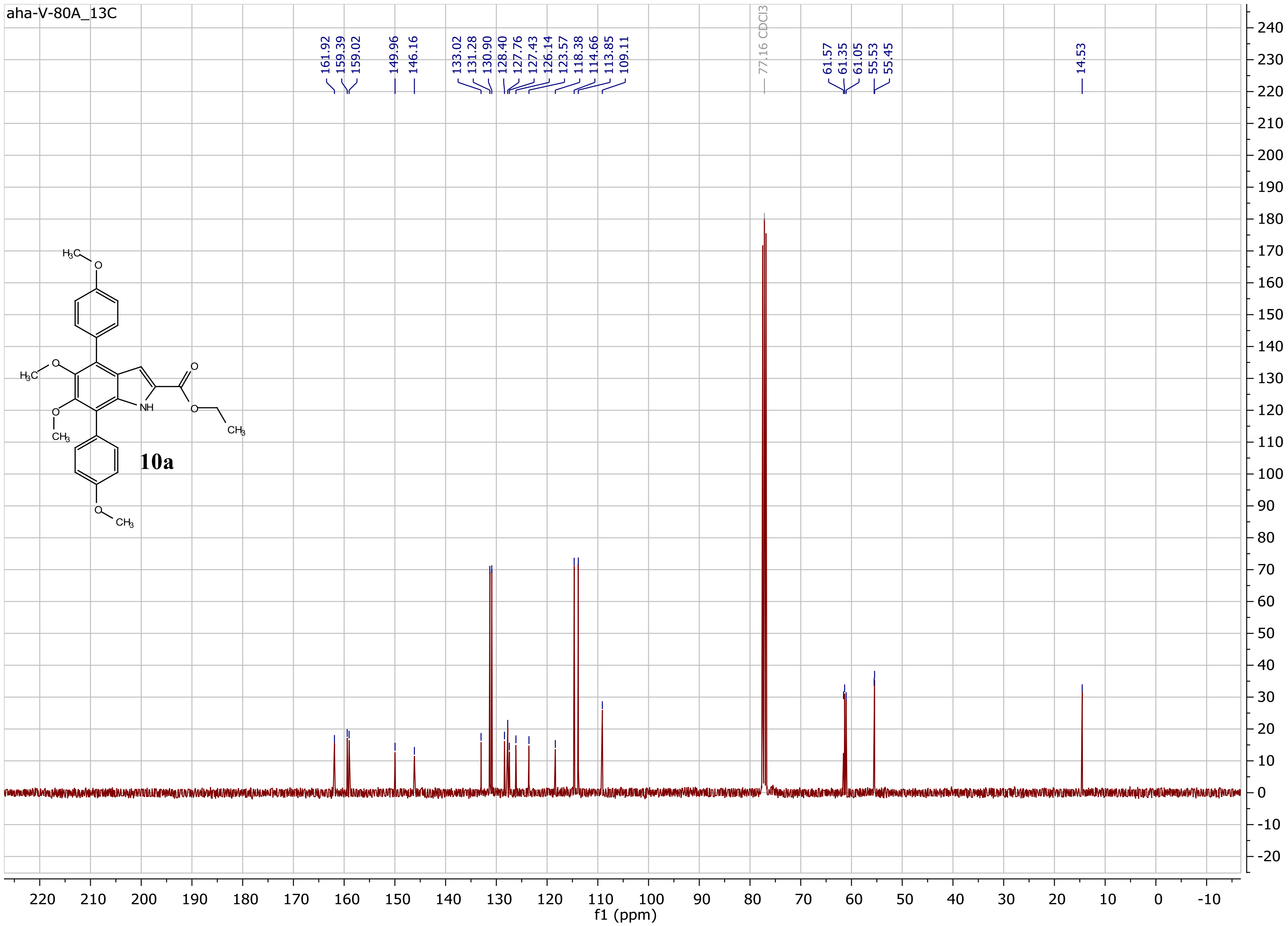
aha-V-80A_13C
161.92
159.39
159.02
149.96
146.16
133.02
131.28
130.90
128.40
127.76
127.43
126.14
123.57
118.38
114.66
113.85
109.11
77.16 CDCl3
61.57
61.35
61.05
55.53
55.45
14.53
10a
f1 (ppm)

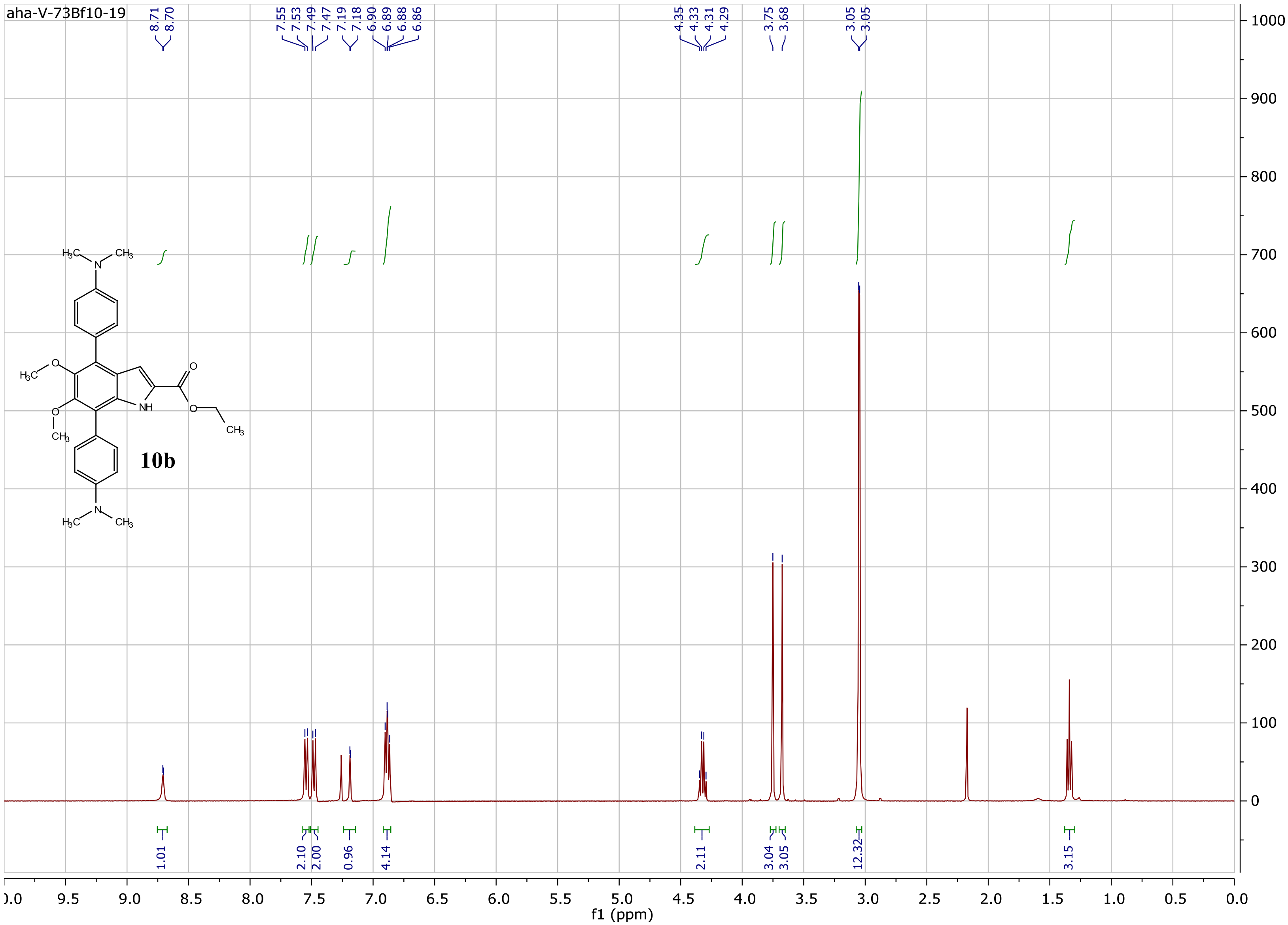
aha-V-73Bf10-19
8.71
8.70
7.55
7.53
7.49
7.47
7.19
7.18
6.90
6.89
6.88
6.86
4.35
4.33
4.31
4.29
3.75
3.68
3.05
3.05
1.01
2.10
2.00
0.96
4.14
2.11
3.04
3.05
12.32
3.15
10b
f1 (ppm)

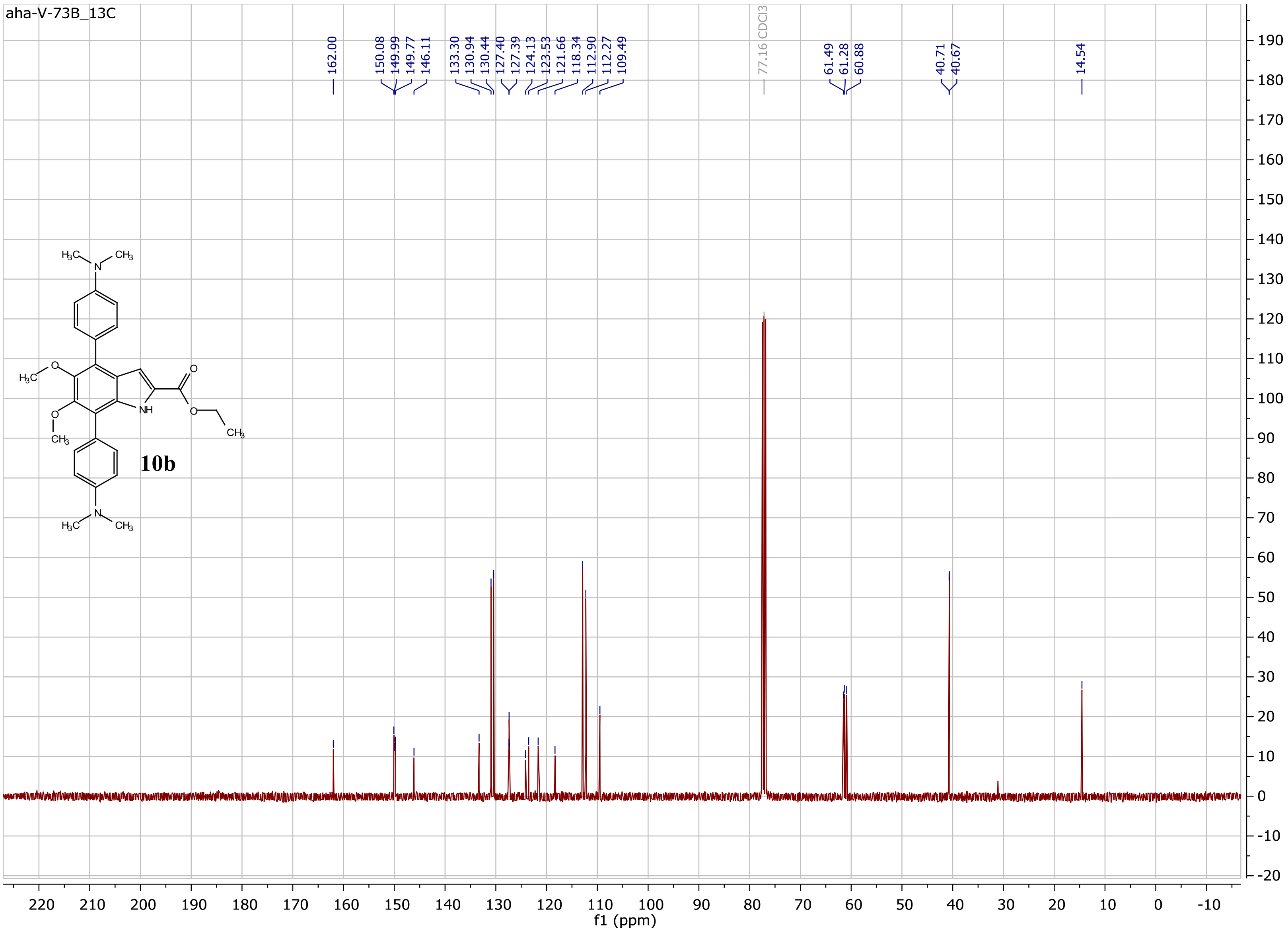

aha-V-73B_13C
162.00
150.08
149.99
149.77
146.11
133.30
130.94
130.44
127.40
127.39
124.13
123.53
121.66
118.34
112.90
112.27
109.49
77.16 CDCl3
61.49
61.28
60.88
40.71
40.67
14.54
10b
f1 (ppm)

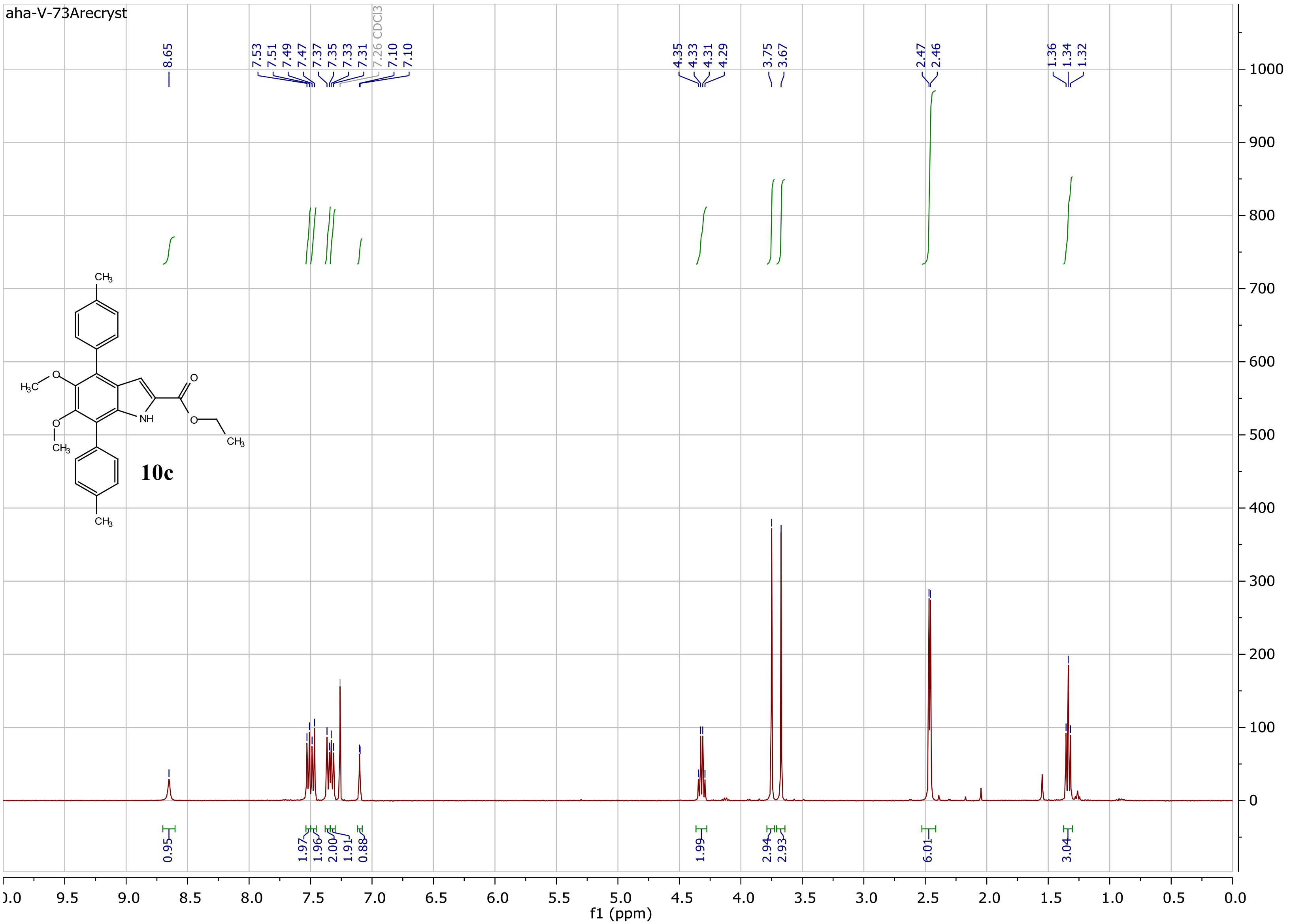
aha-V-73Arecryst
8.65
7.53 7.51 7.49 7.47 7.37 7.35 7.33 7.31 7.26 CDCl3 7.10 7.10
4.35 4.33 4.31 4.29
3.75 3.67
2.47 2.46
1.36 1.34 1.32
0.95 1.97 1.96 2.00 1.91 0.88 1.99 2.94 2.93 6.01 3.04
CH3
H3C
O
O
NH
O
O
CH3
CH3
CH3
10c
f1 (ppm)

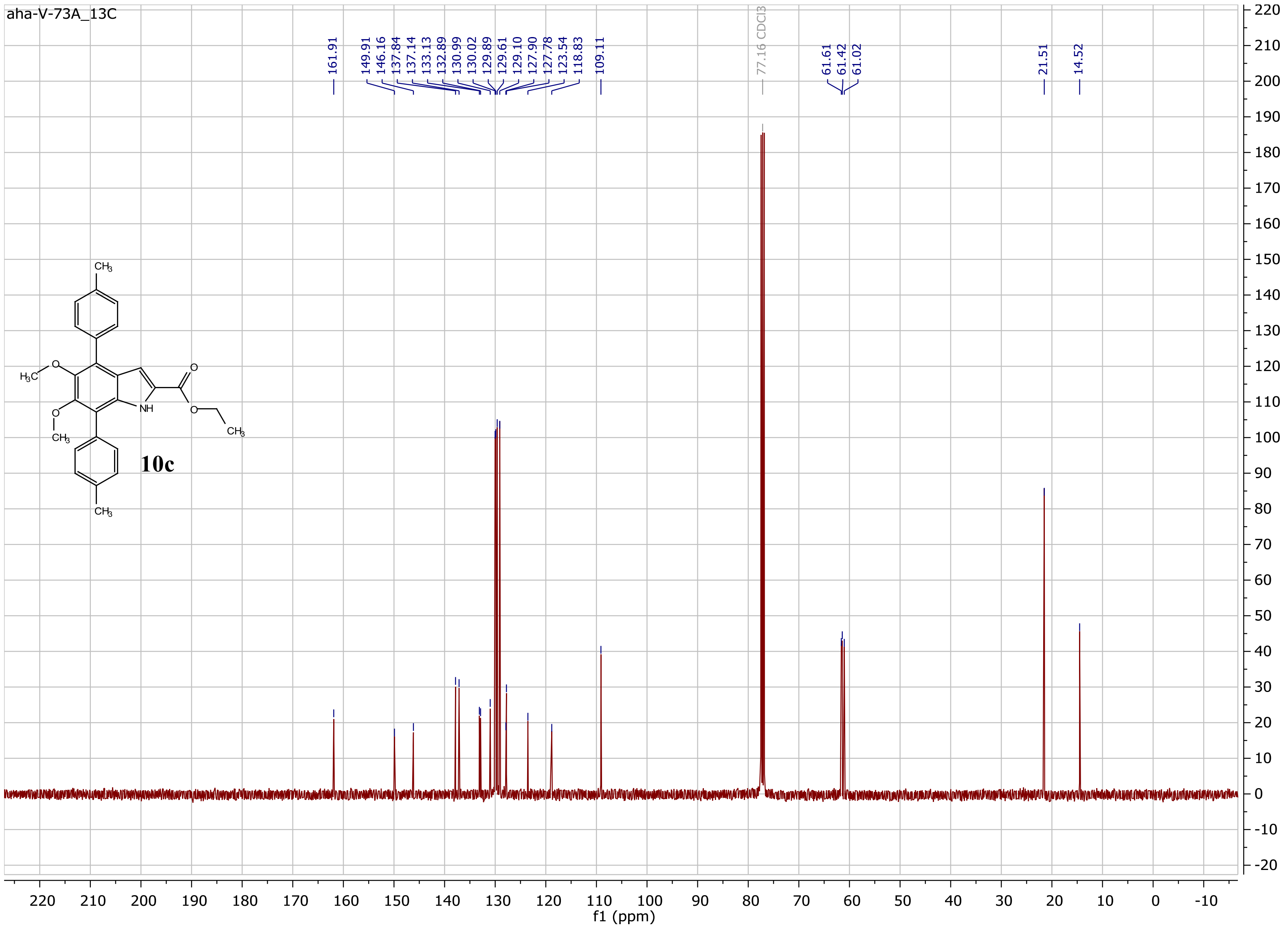
aha-V-73A_13C
161.91
149.91
146.16
137.84
137.14
133.13
132.89
130.99
130.02
129.89
129.61
129.10
127.90
127.78
123.54
118.83
109.11
77.16 CDCl3
61.61
61.42
61.02
21.51
14.52
CH3
H3C
O
O
NH
CH3
CH3
CH3
10c
f1 (ppm)

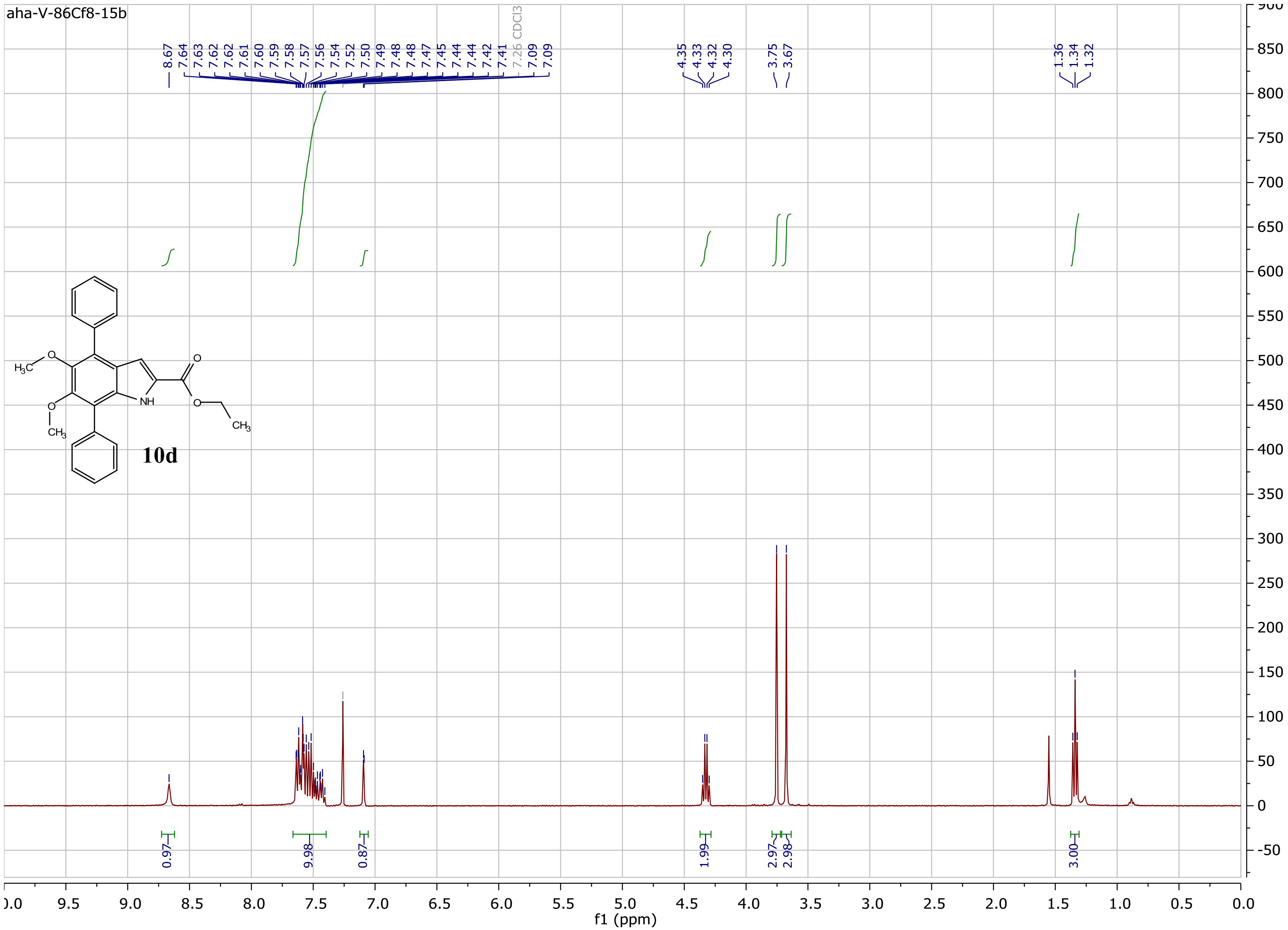

aha-V-86Cf8-15b
8.67
7.64
7.63
7.62
7.61
7.60
7.59
7.58
7.57
7.56
7.54
7.52
7.50
7.49
7.48
7.48
7.47
7.45
7.44
7.44
7.42
7.41
7.26 CDCl3
7.09
7.09
4.35
4.33
4.32
4.30
3.75
3.67
1.36
1.34
1.32
0.97
9.98
0.87
1.99
2.97
2.98
3.00
NH
O
H3C
CH3
f1 (ppm)

10d

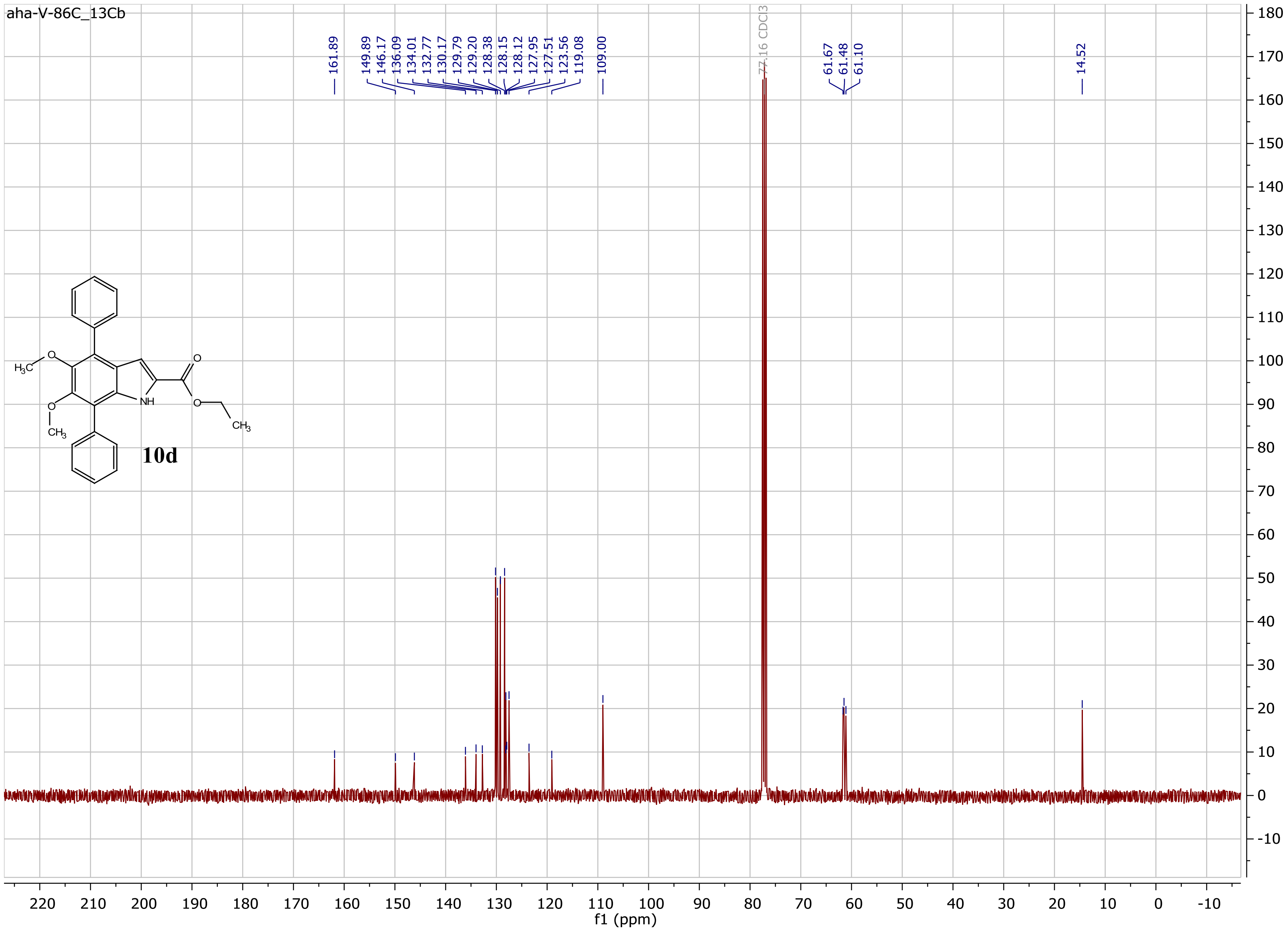

aha-V-86C_13Cb
161.89
149.89
146.17
136.09
134.01
132.77
130.17
129.79
129.20
128.38
128.15
128.12
127.95
127.51
123.56
119.08
109.00
77.16 CDCl3
61.67
61.48
61.10
14.52
10d
f1 (ppm)

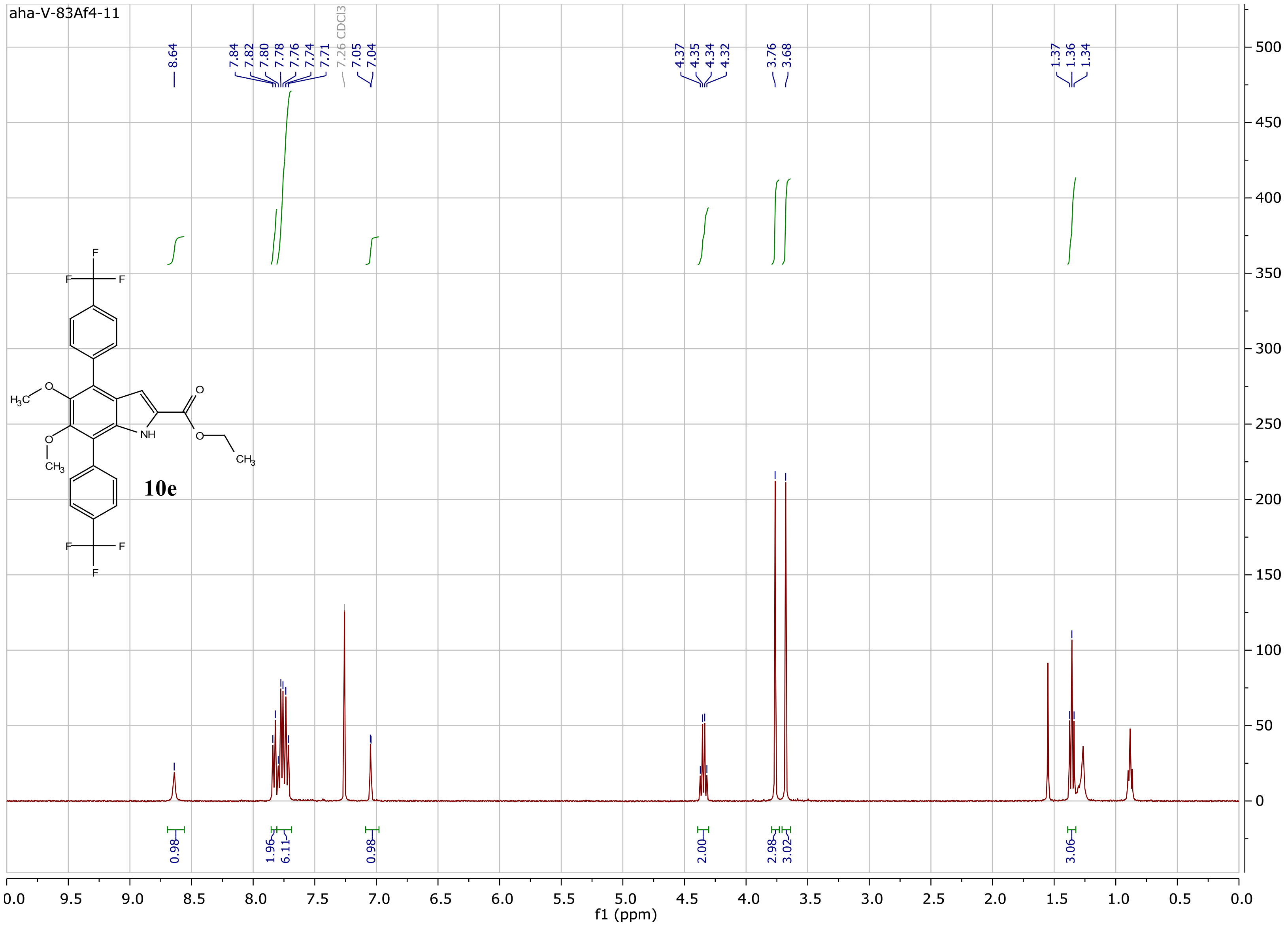

aha-V-83Af4-11
8.64
7.84
7.82
7.80
7.78
7.76
7.74
7.71
7.26 CDCl3
7.05
7.04
4.37
4.35
4.34
4.32
3.76
3.68
1.37
1.36
1.34
0.98
1.96
6.11
0.98
2.00
2.98
3.02
3.06
10e
f1 (ppm)

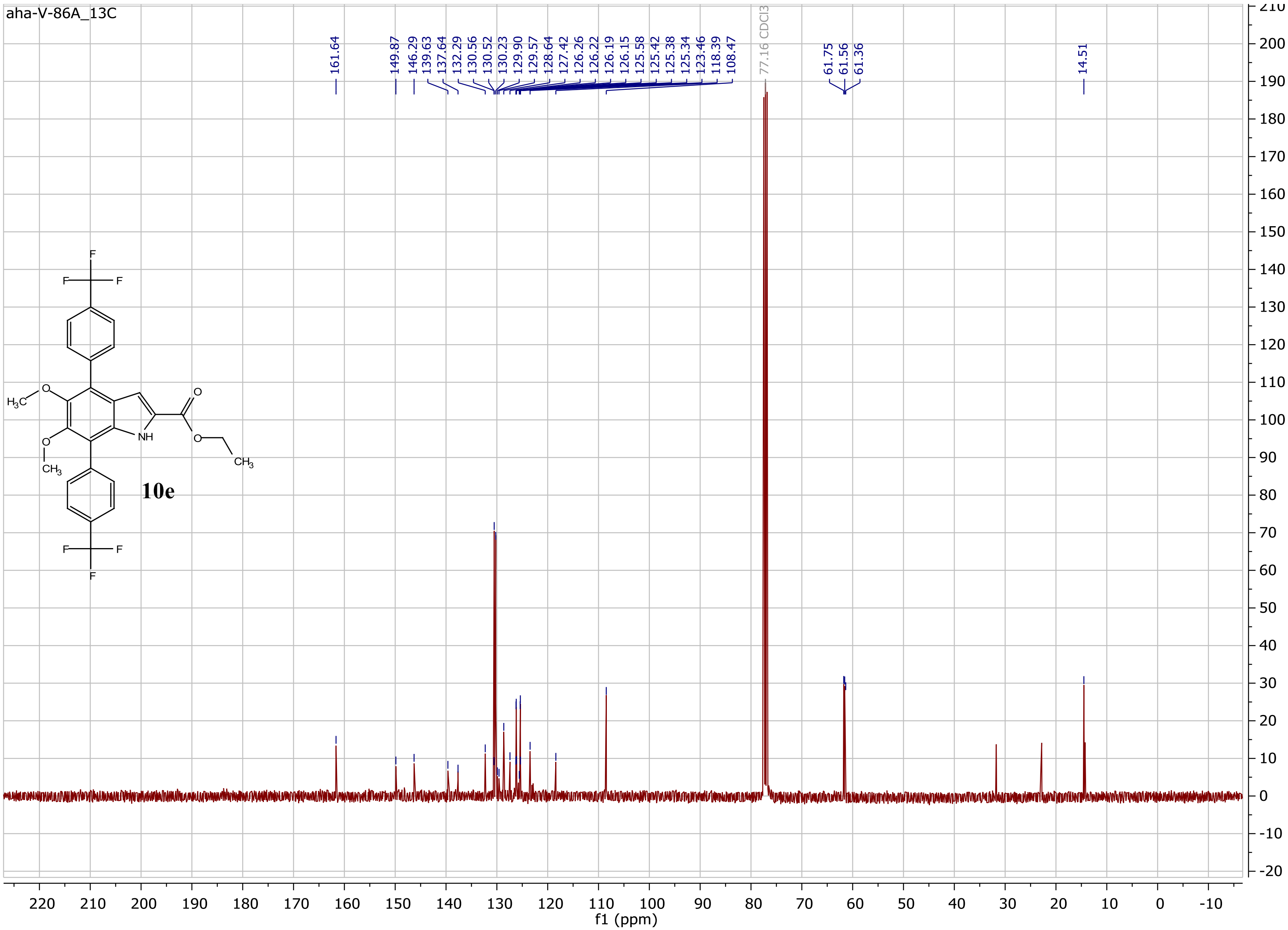

aha-V-86A_13C
161.64
149.87
146.29
139.63
137.64
132.29
130.56
130.52
130.23
129.90
129.57
128.64
127.42
126.26
126.22
126.19
126.15
125.58
125.42
125.38
125.34
123.46
118.39
108.47
77.16 CDCl3
61.75
61.56
61.36
14.51
10e
f1 (ppm)

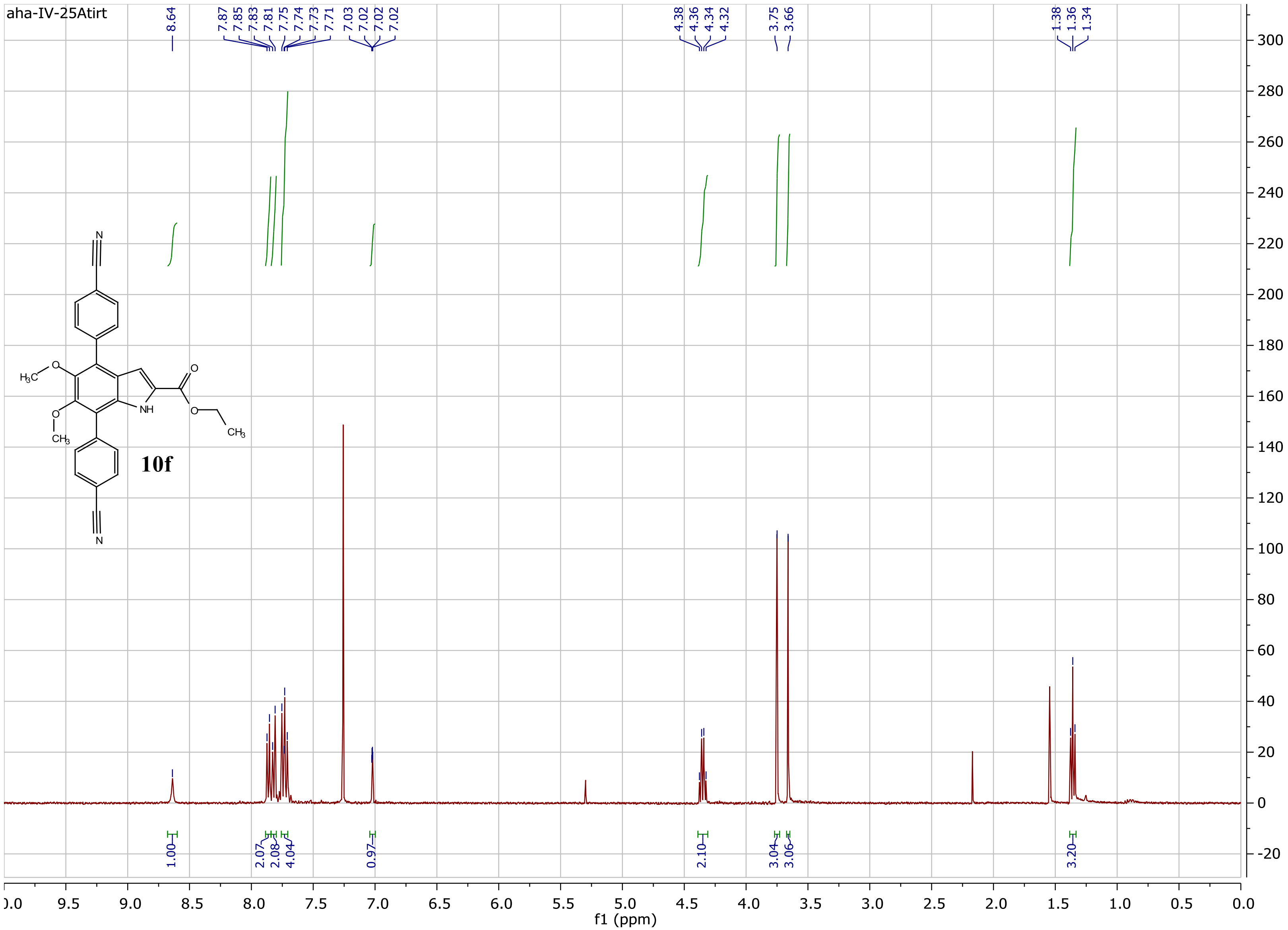
aha-IV-25Atirt
8.64
7.87 7.85 7.83 7.81 7.75 7.74 7.73 7.71
7.03 7.02 7.02 7.02
4.38 4.36 4.34 4.32
3.75 3.66
1.38 1.36 1.34
H3C O O CH3 N N NH O O CH3
10f
1.00 2.07 2.08 4.04 0.97 2.10 3.04 3.06 3.20
f1 (ppm)

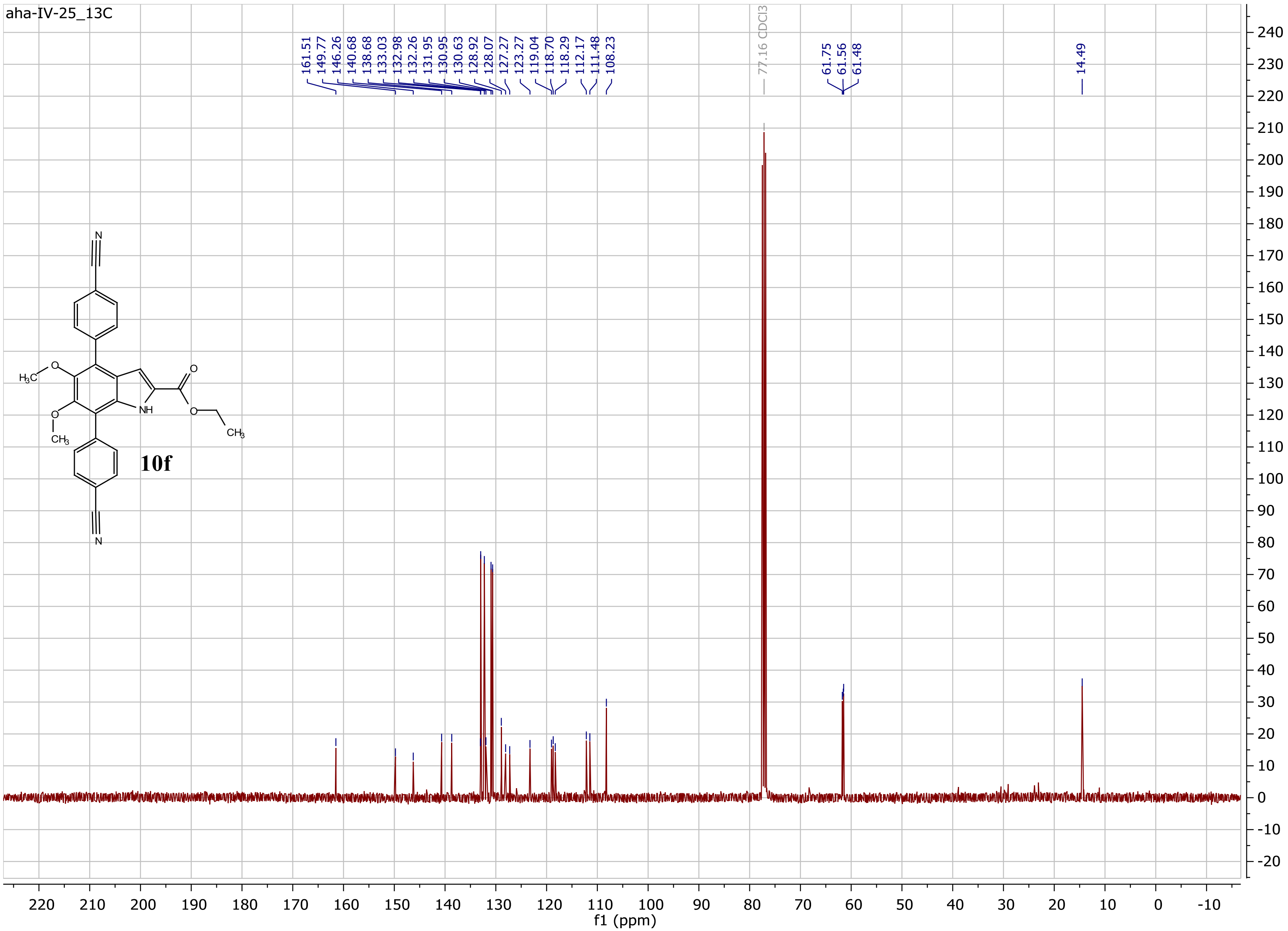

aha-IV-25_13C
161.51
149.77
146.26
140.68
138.68
133.03
132.98
132.26
131.95
130.95
130.63
128.92
128.07
127.27
123.27
119.04
118.70
118.29
112.17
111.48
108.23
77.16 CDCl3
61.75
61.56
61.48
14.49
N
O
O
H_3C
O
O
NH
CH_3
CH_3
N
10f
f1 (ppm)

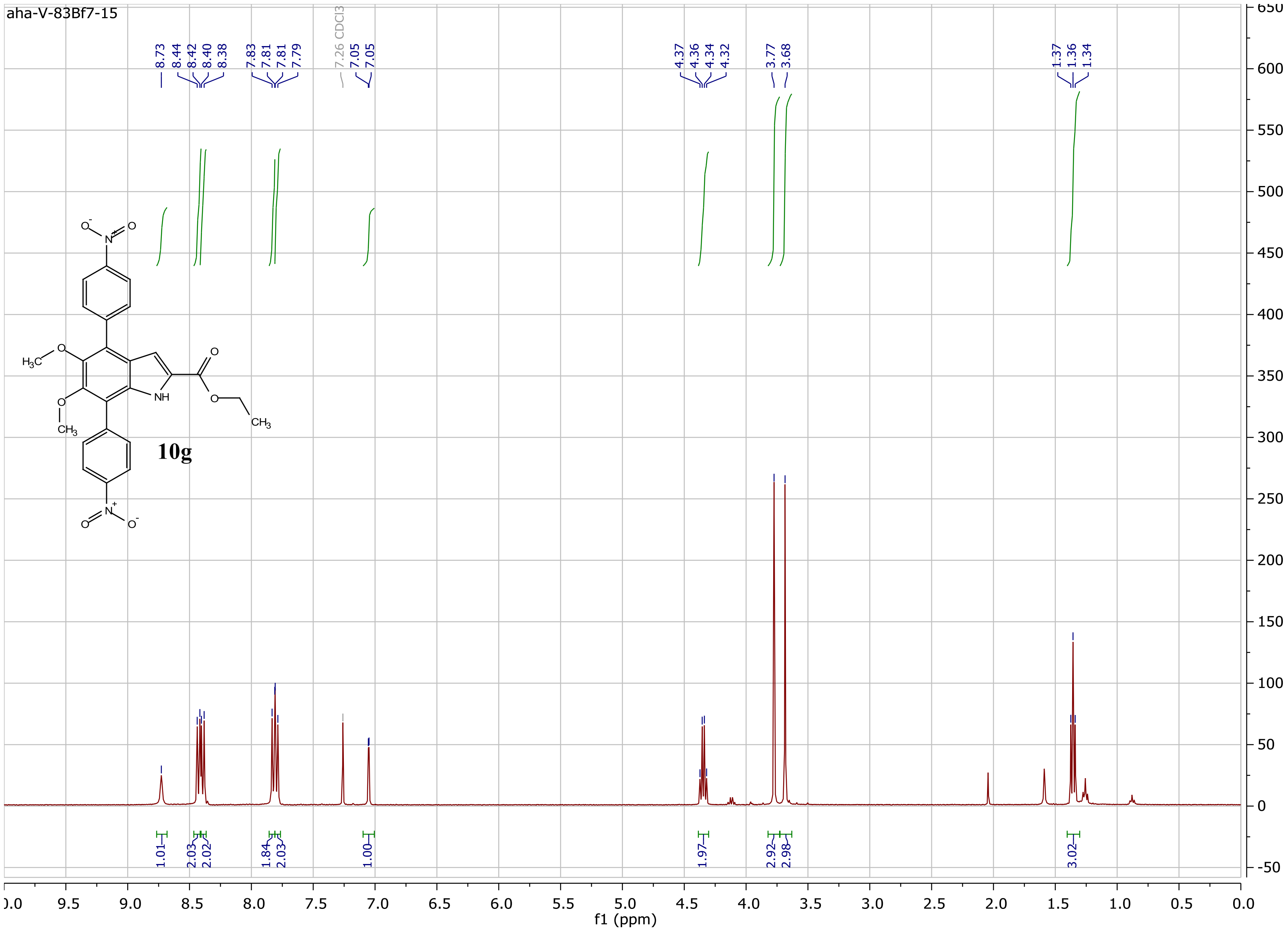
aha-V-83Bf7-15
8.73
8.44
8.42
8.40
8.38
7.83
7.81
7.81
7.79
7.26 CDCl3
7.05
7.05
4.37
4.36
4.34
4.32
3.77
3.68
1.37
1.36
1.34
10g
1.01
2.03
2.02
1.84
2.03
1.00
1.97
2.92
2.98
3.02
f1 (ppm)

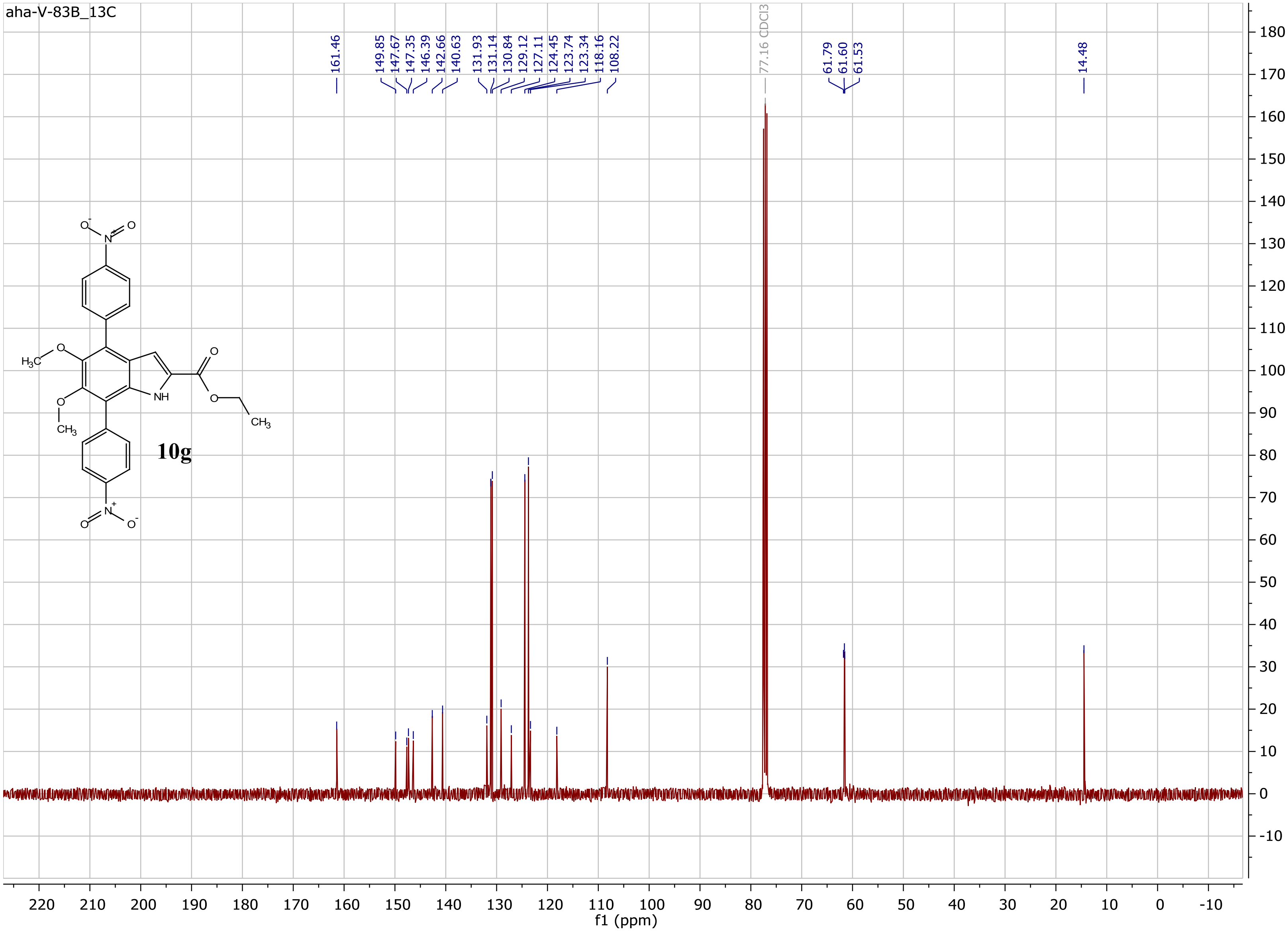

aha-V-83B_13C
161.46
149.85
147.67
147.35
146.39
142.66
140.63
131.93
131.14
130.84
129.12
127.11
124.45
123.74
123.34
118.16
108.22
77.16 CDCl3
61.79
61.60
61.53
14.48
10g
f1 (ppm)

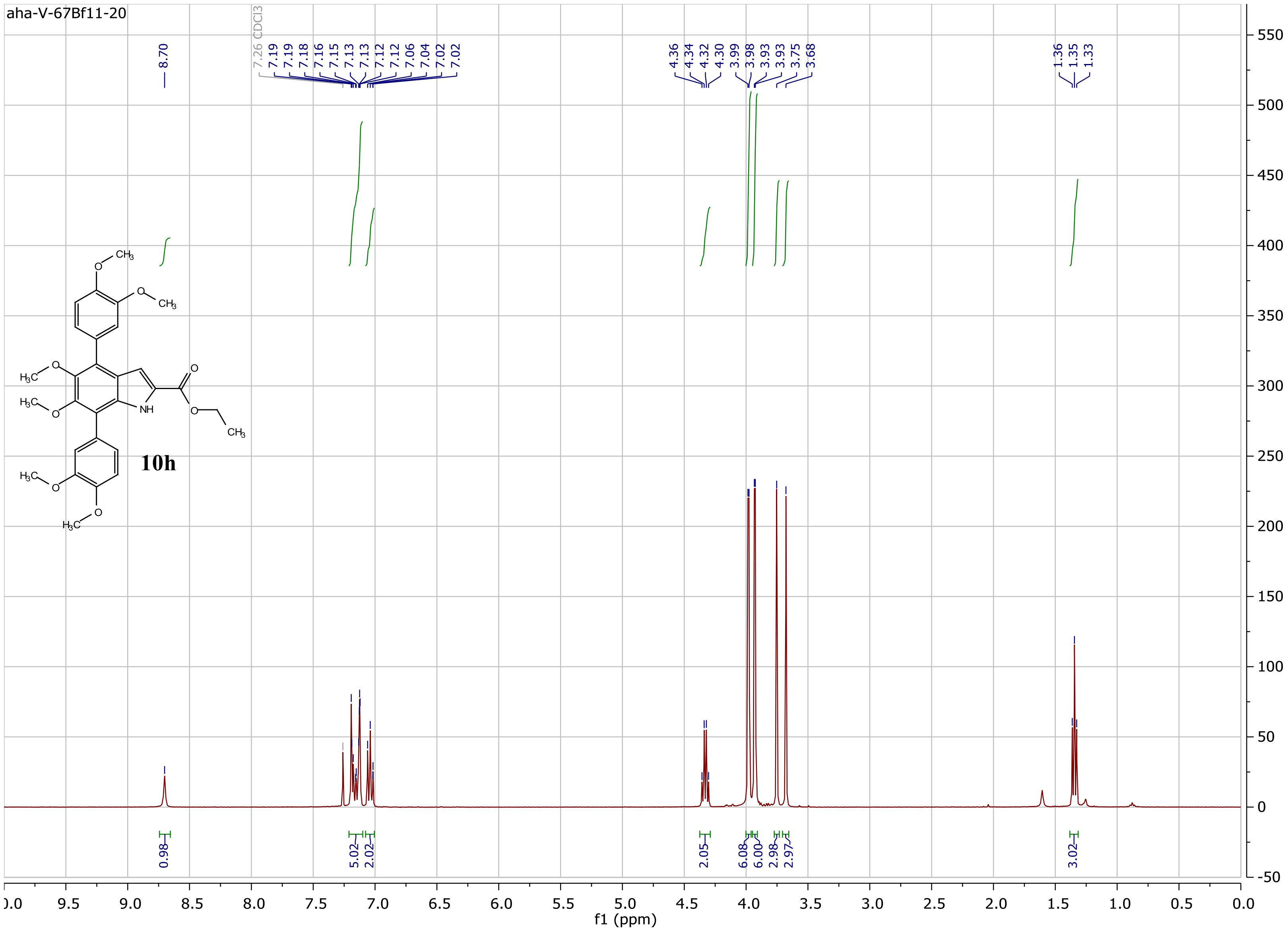
aha-V-67Bf11-20
10h
f1 (ppm)

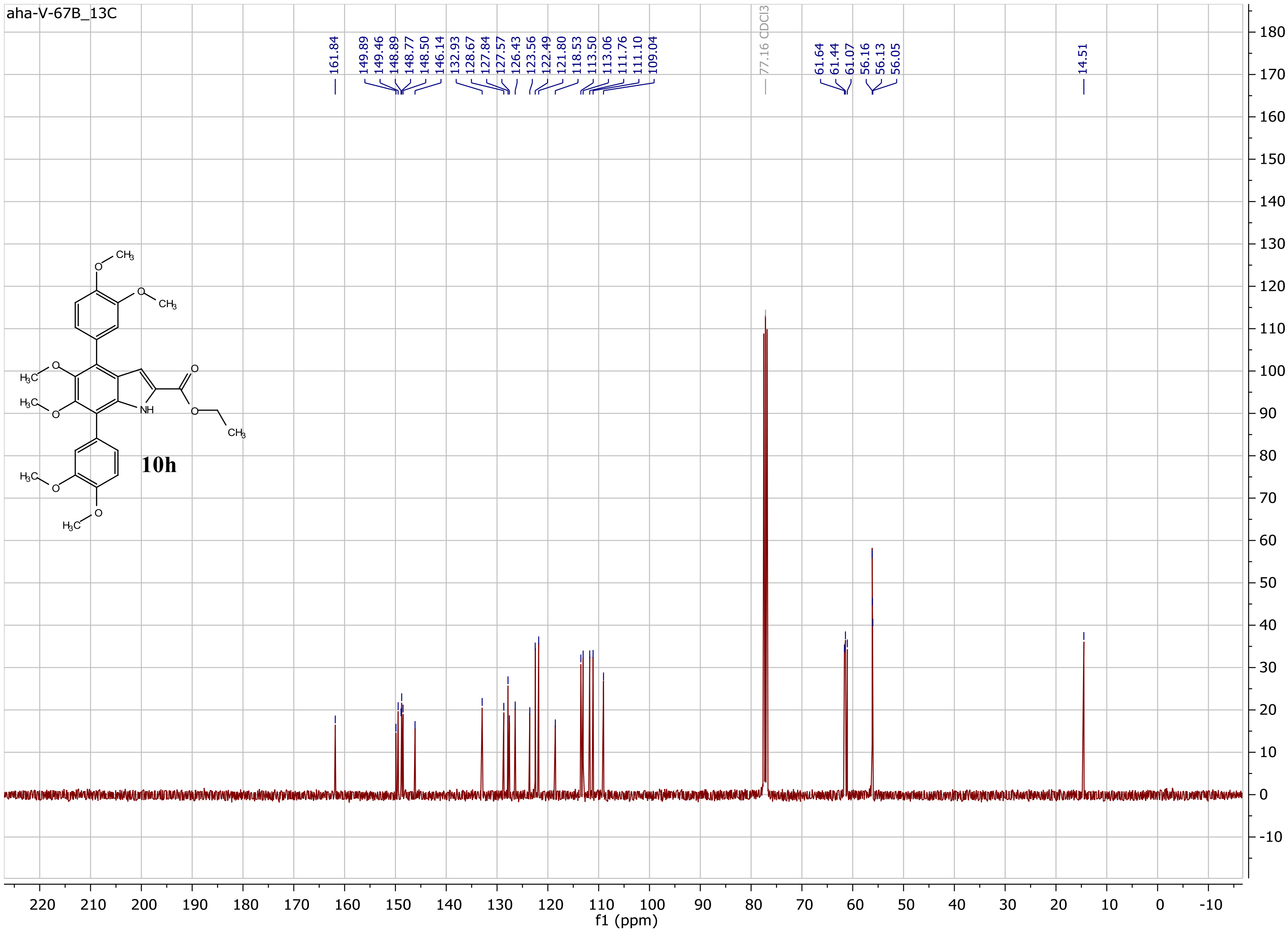
aha-V-67B_13C
161.84
149.89
149.46
148.89
148.77
148.50
146.14
132.93
128.67
127.84
127.57
126.43
123.56
122.49
121.80
118.53
113.50
113.06
111.76
111.10
109.04
77.16 CDCl3
61.64
61.44
61.07
56.16
56.13
56.05
14.51
10h
f1 (ppm)

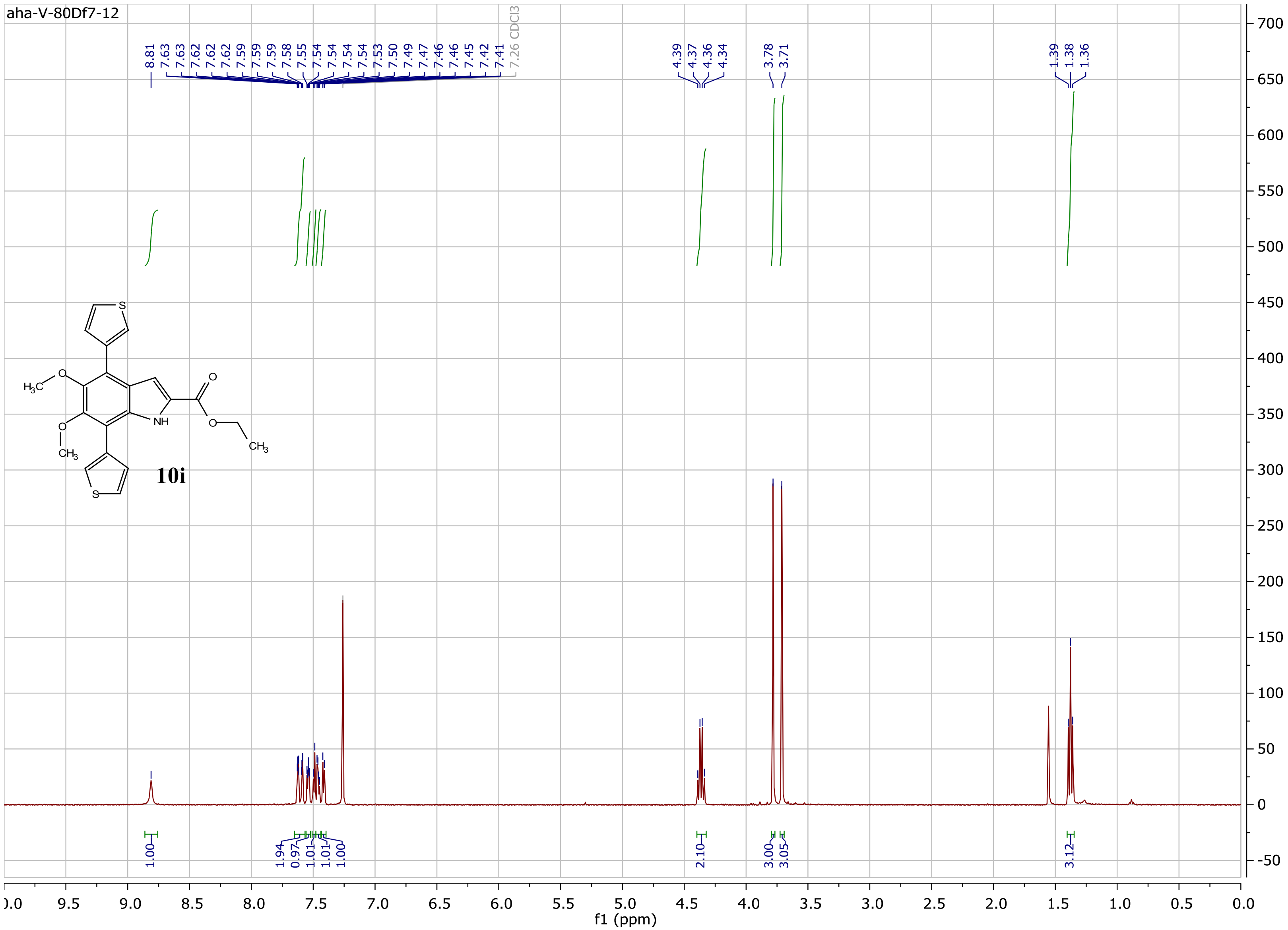
aha-V-80Df7-12
8.81
7.63
7.63
7.62
7.62
7.62
7.59
7.59
7.59
7.58
7.55
7.54
7.54
7.54
7.54
7.53
7.50
7.49
7.47
7.46
7.46
7.45
7.42
7.41
7.26 CDCl3
4.39
4.37
4.36
4.34
3.78
3.71
1.39
1.38
1.36
1.00
1.94
0.97
1.01
1.01
1.00
2.10
3.00
3.05
3.12
10i
f1 (ppm)

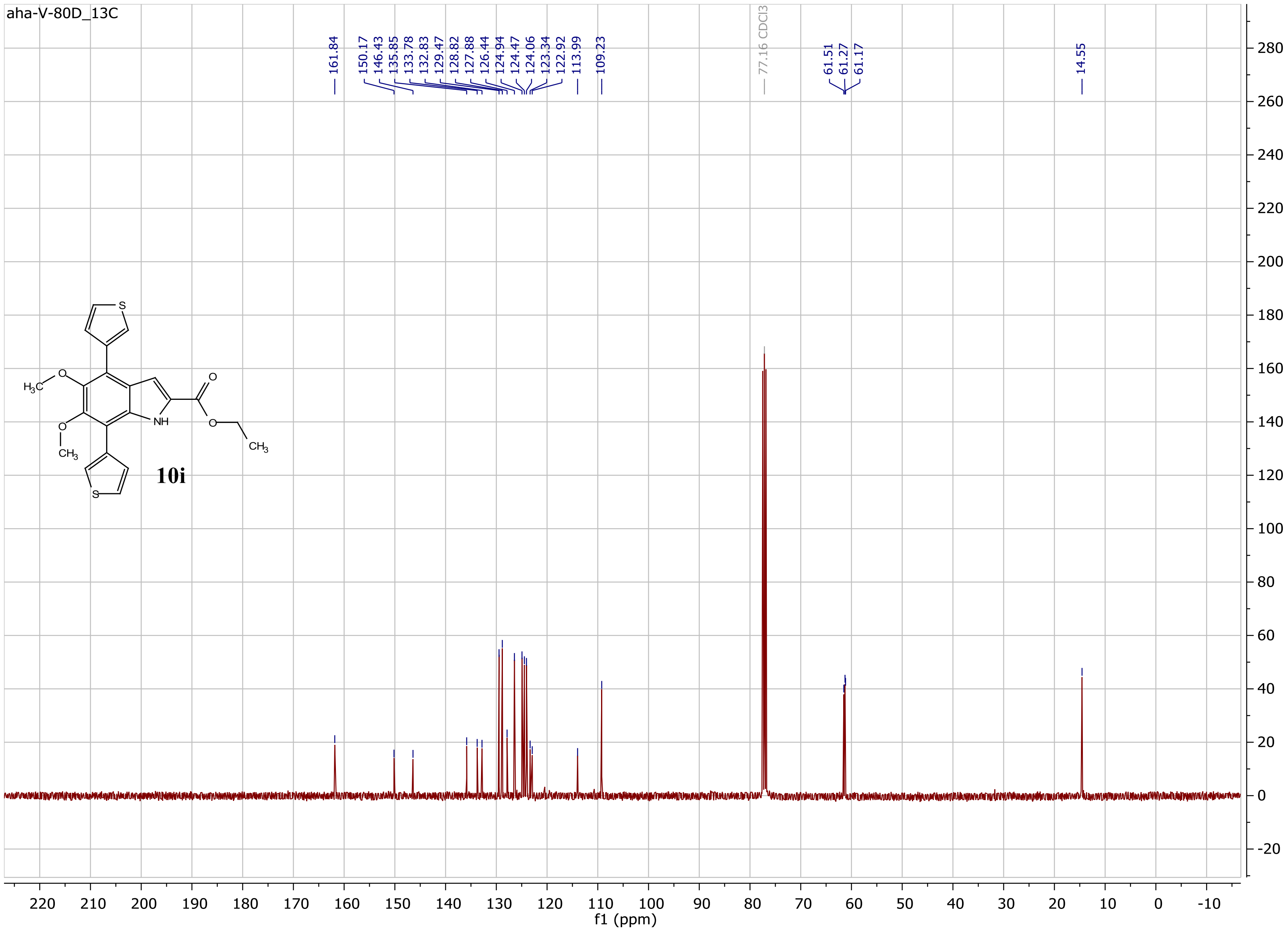
aha-V-80D_13C
161.84
150.17
146.43
135.85
133.78
132.83
129.47
128.82
127.88
126.44
124.94
124.47
124.06
123.34
122.92
113.99
109.23
77.16 CDCl3
61.51
61.27
61.17
14.55
10i
f1 (ppm)

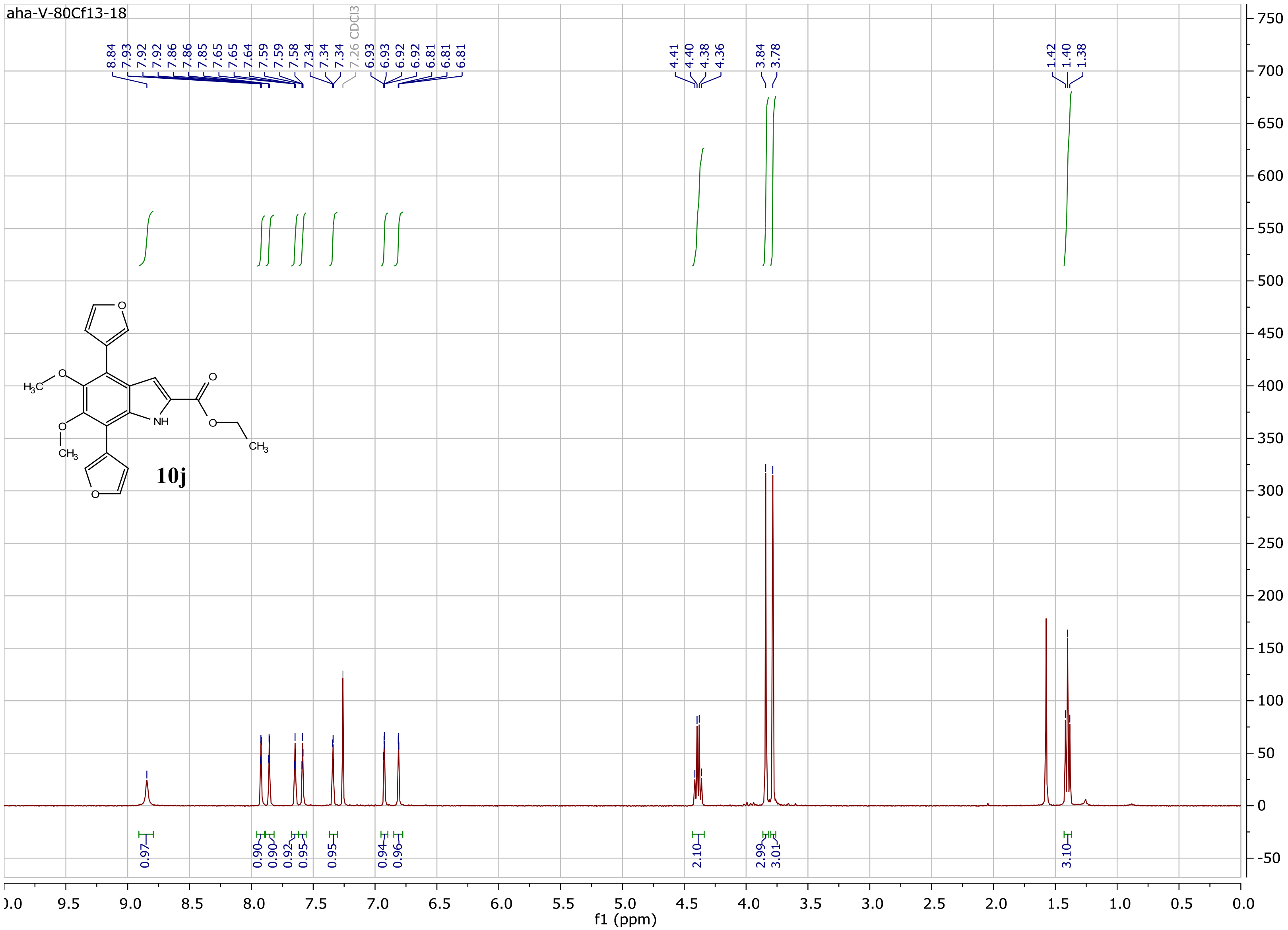

aha-V-80Cf13-18
8.84
7.93
7.92
7.92
7.86
7.86
7.85
7.65
7.65
7.64
7.59
7.59
7.58
7.34
7.34
7.34
7.26 CDCl3
6.93
6.93
6.92
6.92
6.81
6.81
4.41
4.40
4.38
4.36
3.84
3.78
1.42
1.40
1.38
10j
0.97
0.90
0.90
0.92
0.95
0.95
0.94
0.96
2.10
2.99
3.01
3.10
f1 (ppm)

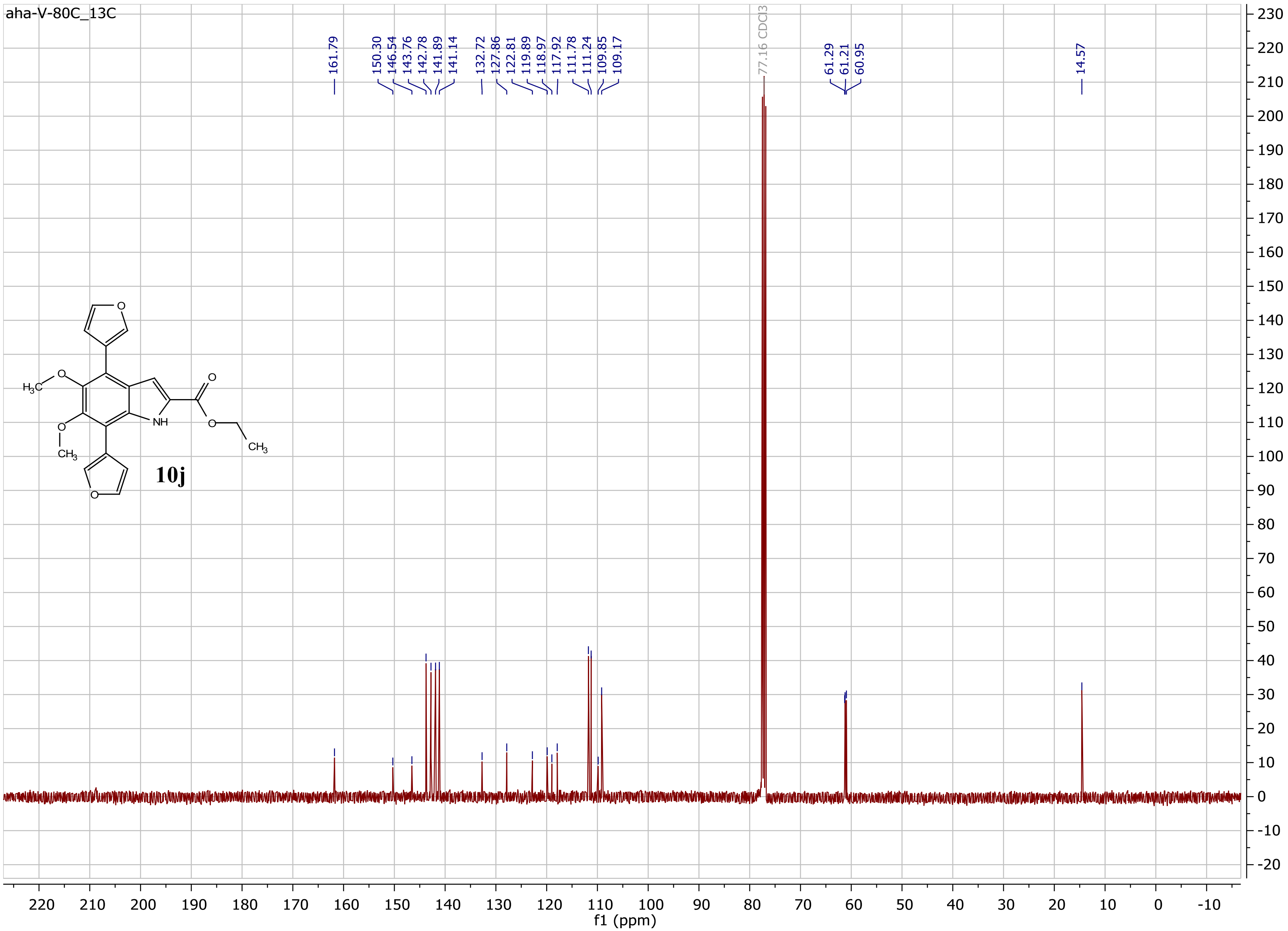
aha-V-80C_13C
161.79
150.30
146.54
143.76
142.78
141.89
141.14
132.72
127.86
122.81
119.89
118.97
117.92
111.78
111.24
109.85
109.17
77.16 CDCl3
61.29
61.21
60.95
14.57
10j
f1 (ppm)

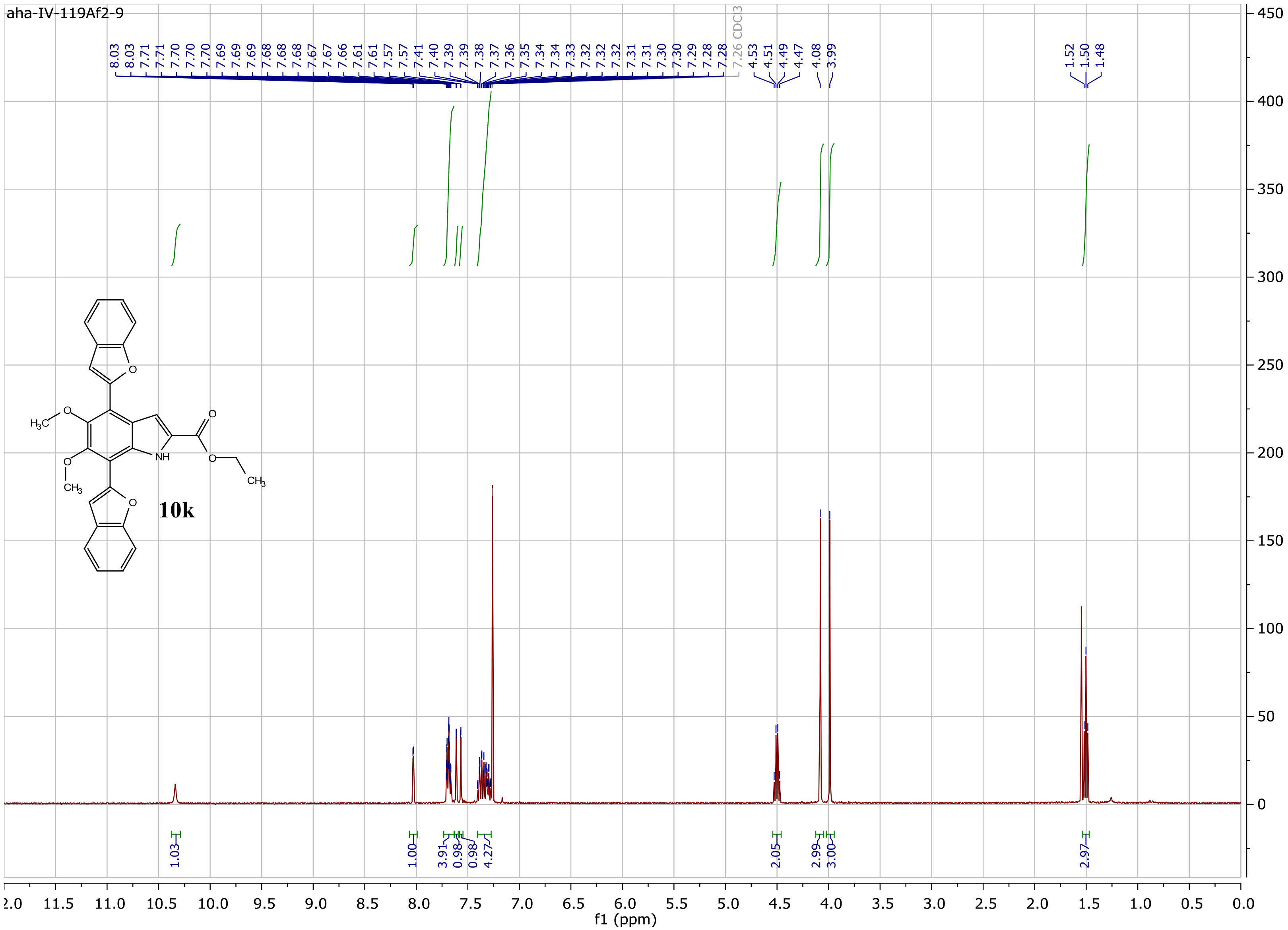
aha-IV-119Af2-9
8.03 8.03 7.71 7.71 7.70 7.70 7.70 7.69 7.69 7.69 7.68 7.68 7.68 7.67 7.67 7.66 7.61 7.61 7.61 7.57 7.57 7.41 7.40 7.39 7.39 7.38 7.37 7.36 7.35 7.34 7.34 7.33 7.32 7.32 7.32 7.31 7.31 7.30 7.30 7.29 7.28 7.28
7.26 CDC3
4.53 4.51 4.49 4.47
4.08 3.99
1.52 1.50 1.48
10k
1.03 1.00 3.91 0.98 0.98 4.27 2.05 2.99 3.00 2.97
f1 (ppm)

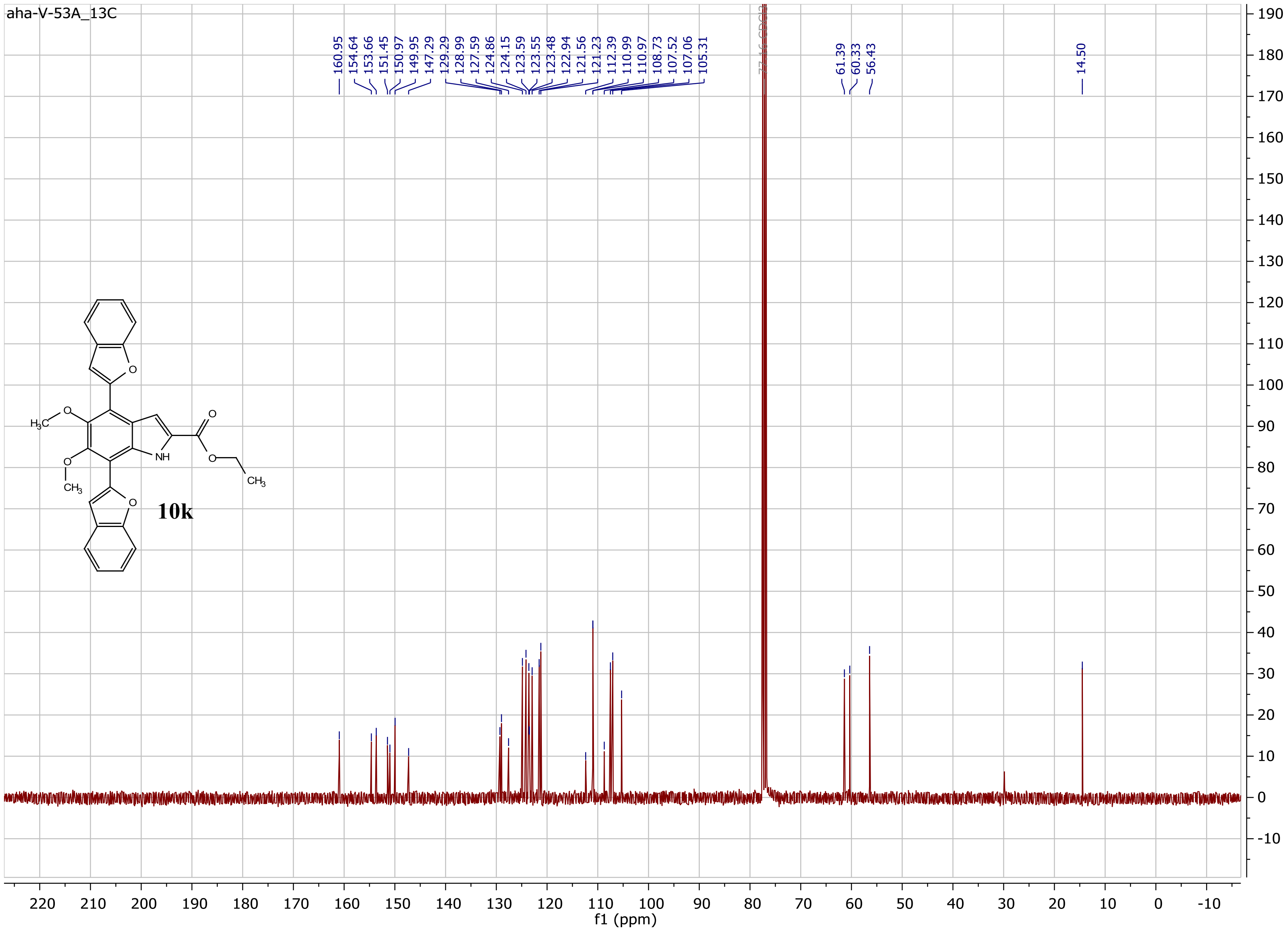
aha-V-53A_13C
160.95
154.64
153.66
151.45
150.97
149.95
147.29
129.29
128.99
127.59
124.86
124.15
123.59
123.55
123.48
122.94
121.56
121.23
112.39
110.99
110.97
108.73
107.52
107.06
105.31
61.39
60.33
56.43
14.50
10k
f1 (ppm)

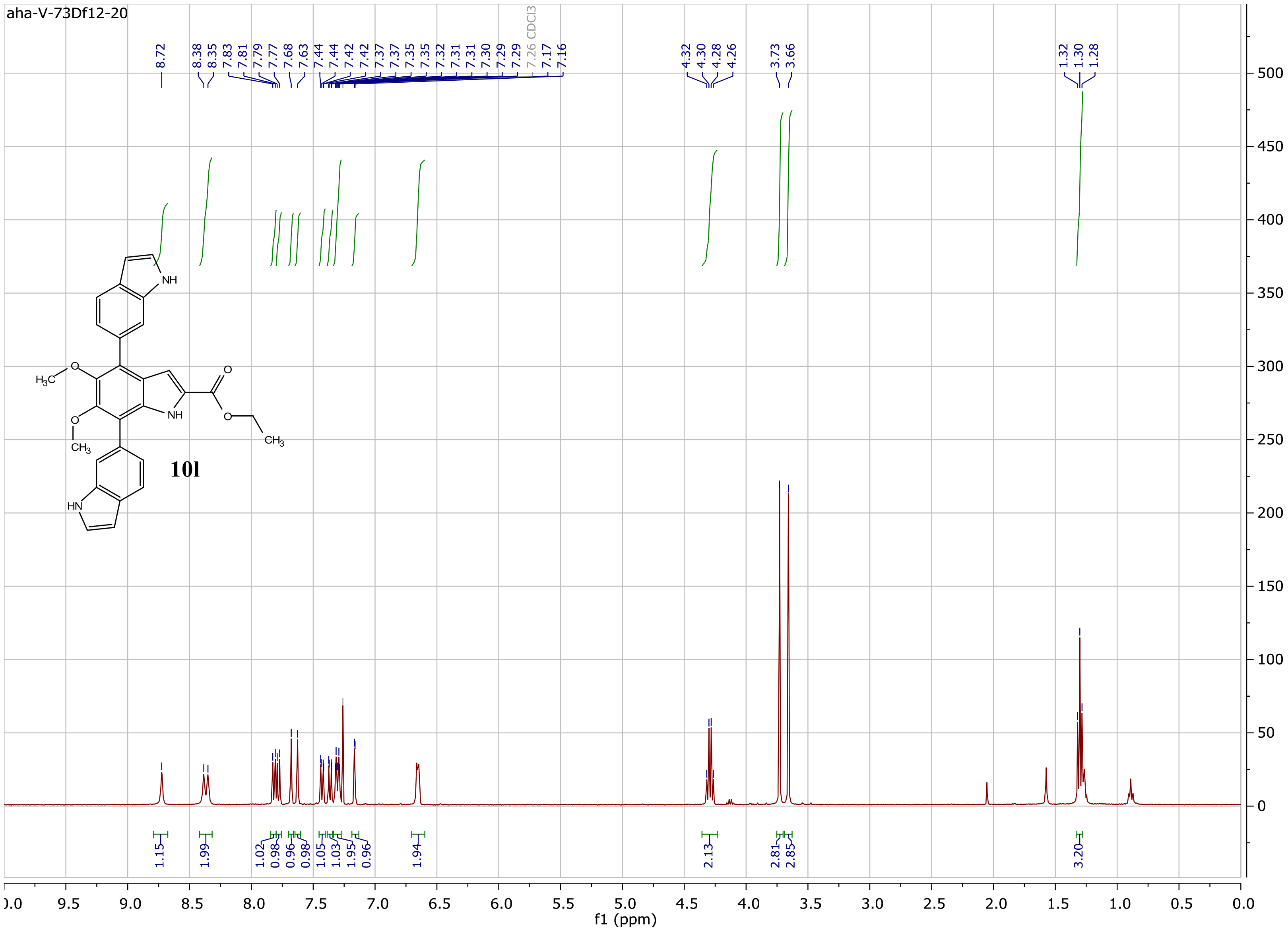
aha-V-73Df12-20
8.72
8.38
8.35
7.83
7.81
7.79
7.77
7.68
7.63
7.44
7.44
7.42
7.42
7.37
7.37
7.35
7.35
7.32
7.31
7.31
7.30
7.29
7.29
7.26 CDCl3
7.17
7.16
4.32
4.30
4.28
4.26
3.73
3.66
1.32
1.30
1.28
NH
O
H3C
O
O
CH3
NH
O
CH3
HN
10l
1.15
1.99
1.02
0.98
0.96
0.98
1.05
1.03
1.95
0.96
1.94
2.13
2.81
2.85
3.20
f1 (ppm)

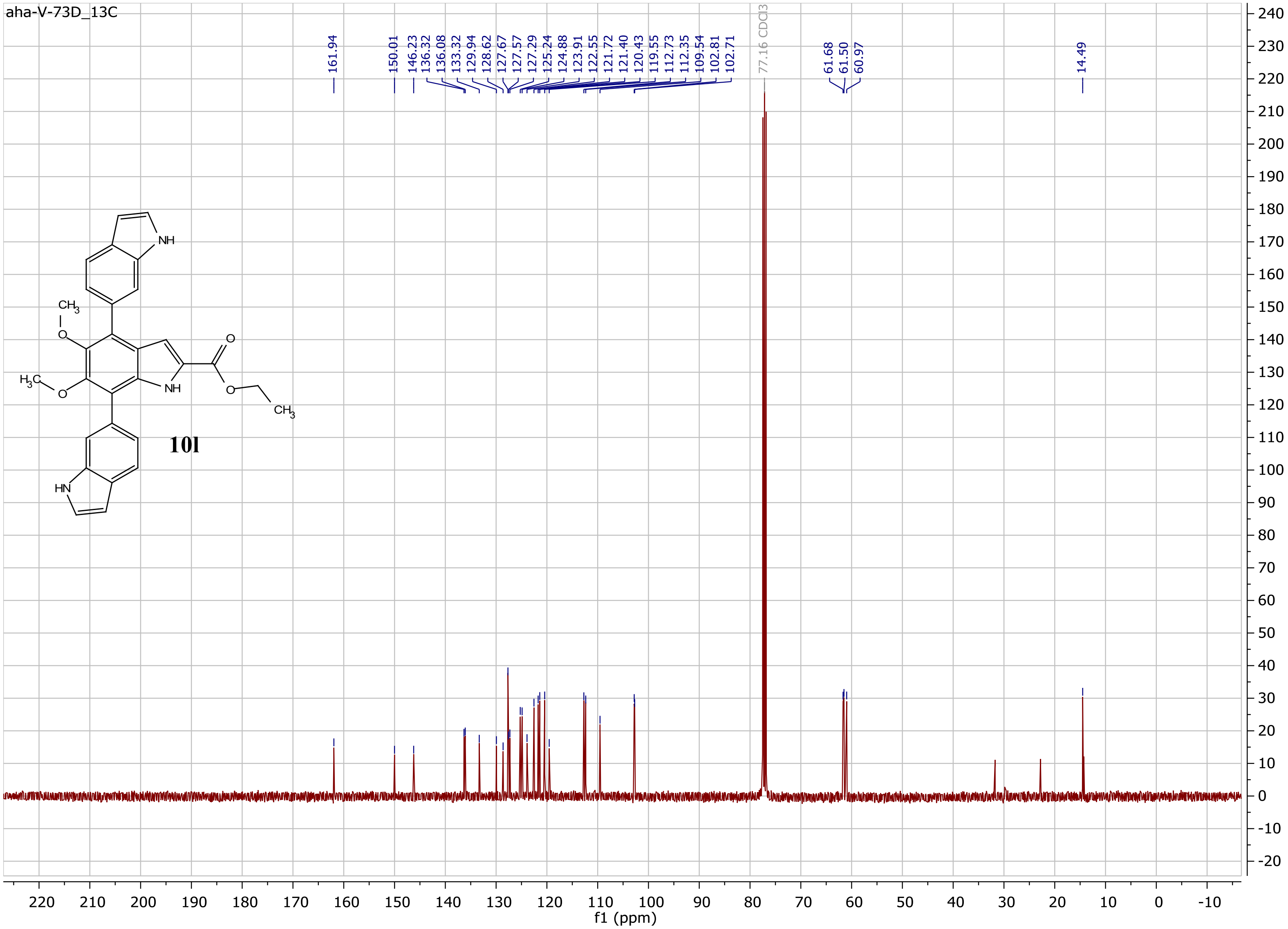

10l
aha-V-73D_13C
161.94
150.01
146.23
136.32
136.08
133.32
129.94
128.62
127.67
127.57
127.29
125.24
124.88
123.91
122.55
121.72
121.40
120.43
119.55
112.73
112.35
109.54
102.81
102.71
77.16 CDCl3
61.68
61.50
60.97
14.49
f1 (ppm)

High Resolution Mass Spectra

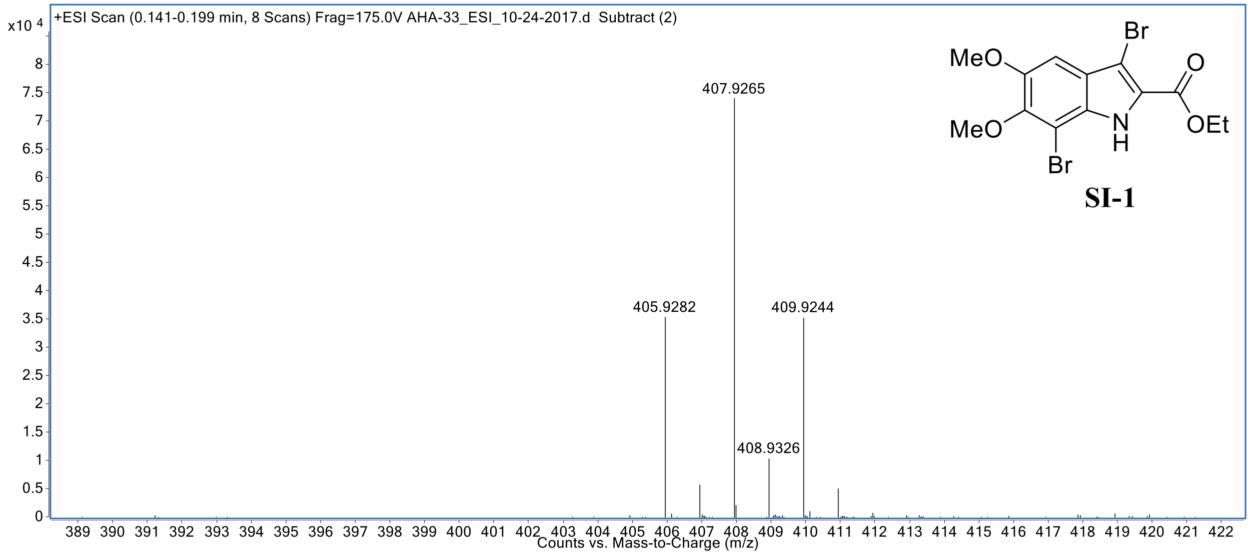

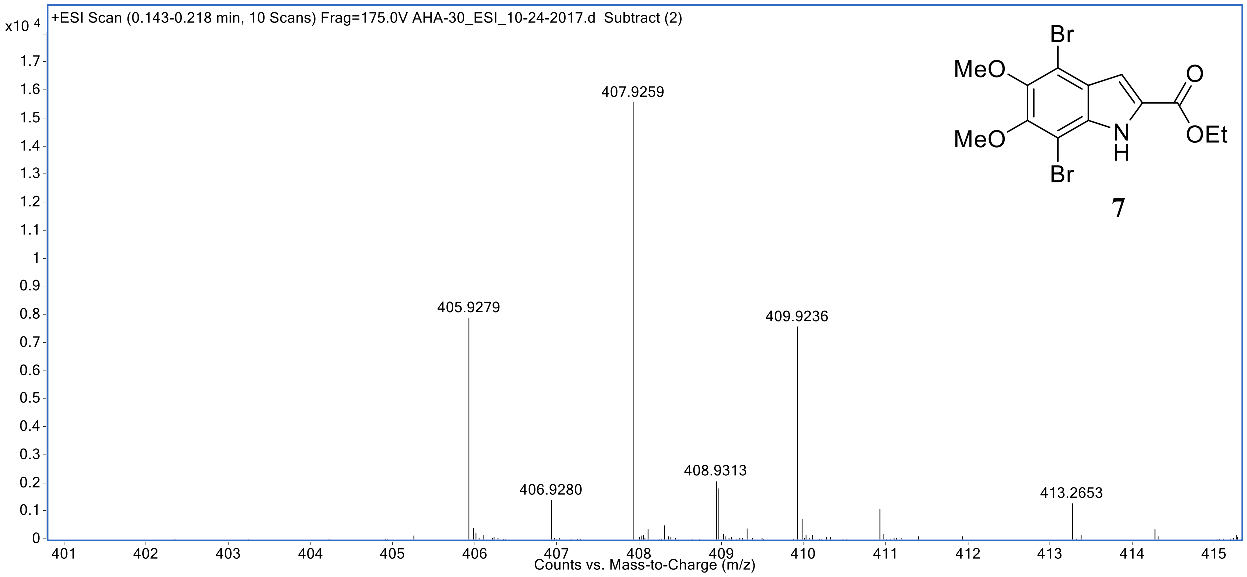

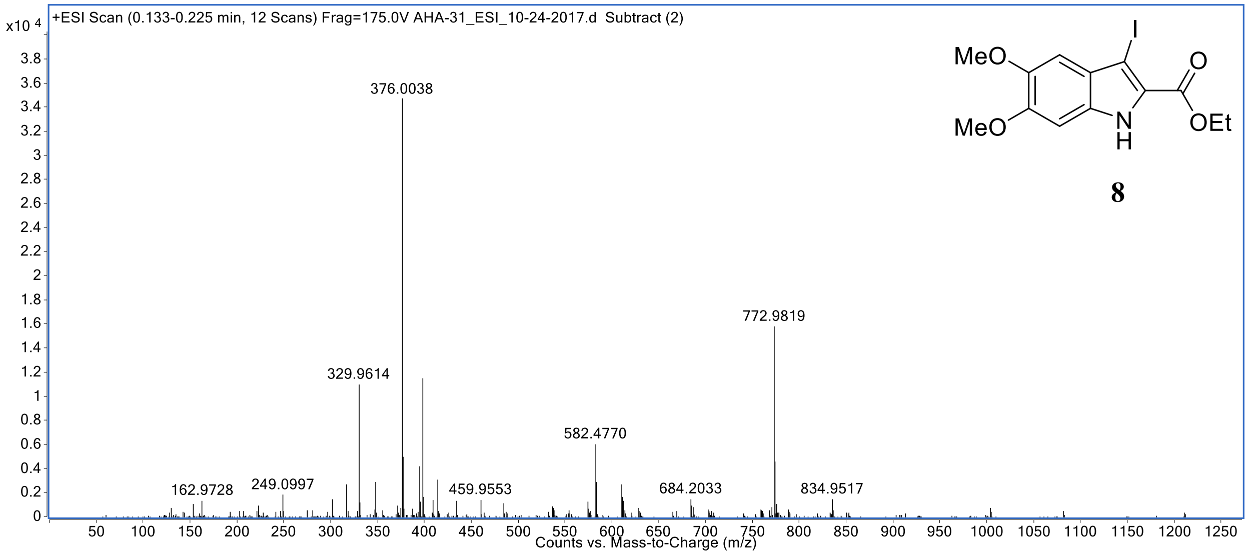
+ESI Scan (0.133-0.225 min, 12 Scans) Frag=175.0V AHA-31_ESI_10-24-2017.d Subtract (2)
x10 4
376.0038
772.9819
329.9614
582.4770
162.9728
249.0997
459.9553
684.2033
834.9517
Counts vs. Mass-to-Charge (m/z)
MeO
MeO
I
O
N
H
OEt
8

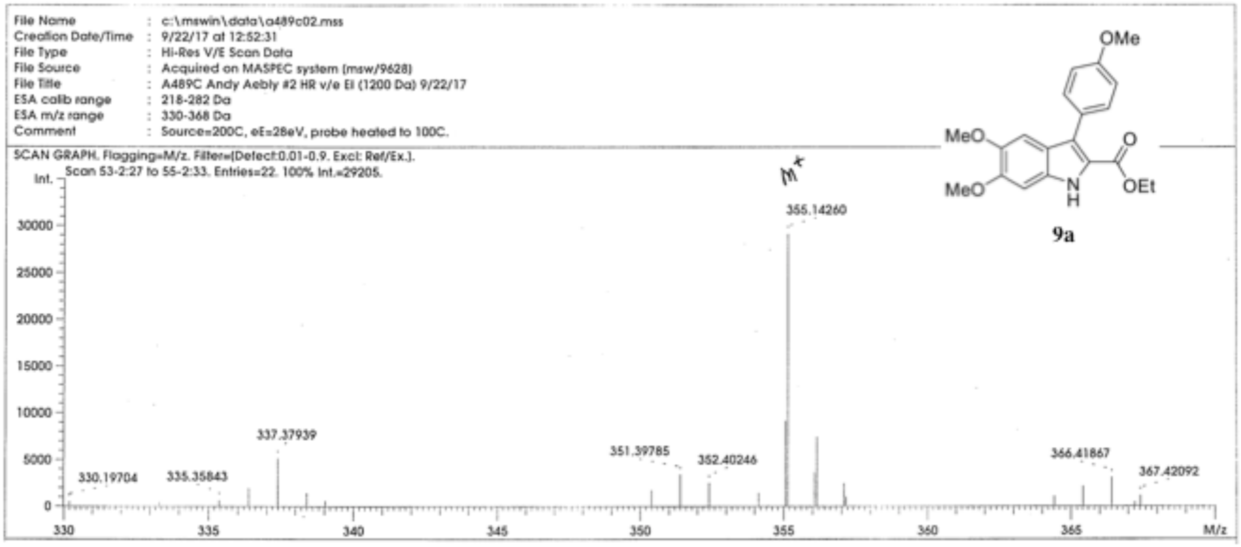
File Name : c:\mswin\data\a489c02.mss
Creation Date/Time : 9/22/17 at 12:52:31
File Type : Hi-Res V/E Scan Data
File Source : Acquired on MASPEC system (msw/9628)
File Title : A489C Andy Aebly #2 HR v/e EI (1200 Da) 9/22/17
ESA calib range : 218-282 Da
ESA m/z range : 330-368 Da
Comment : Source=200C, eE=28eV, probe heated to 100C.
SCAN GRAPH. Flagging=M/z. Filter=(Defect:0.01-0.9. Excl: Ref/Ex.).
Scan 53-2:27 to 55-2:33. Entries=22. 100% Int.=29205.
Int.
M+
355.14260
337.37939
330.19704
335.35843
351.39785
352.40246
366.41867
367.42092
M/z
OMe
MeO
MeO
O
N
H
OEt
9a

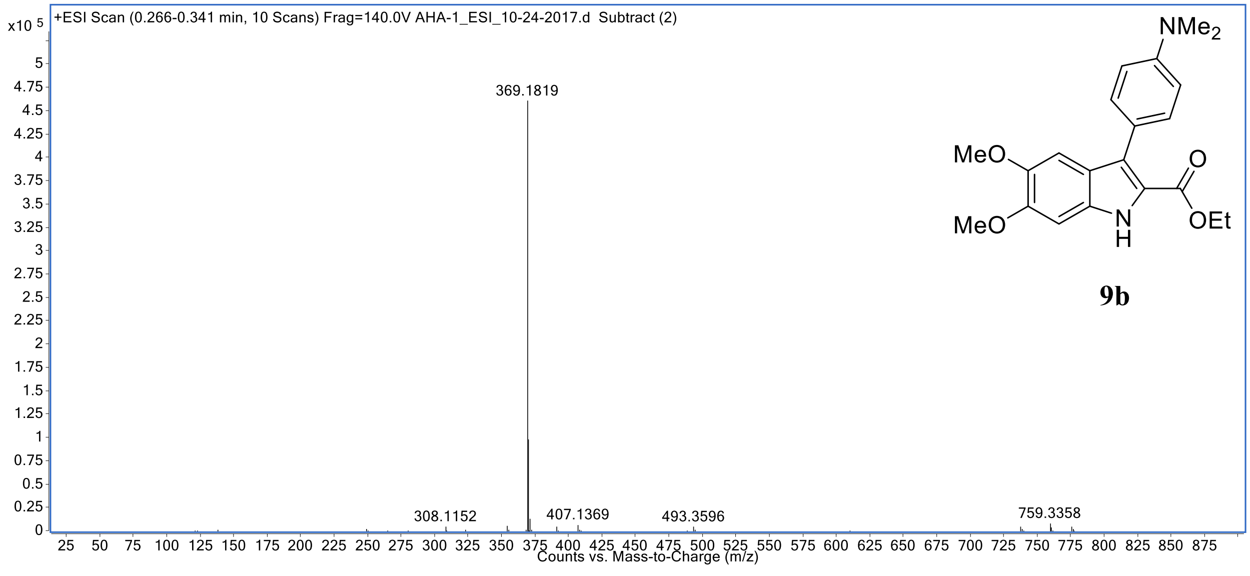
+ESI Scan (0.266-0.341 min, 10 Scans) Frag=140.0V AHA-1_ESI_10-24-2017.d Subtract (2)
x10 5
369.1819
308.1152
407.1369
493.3596
759.3358
Counts vs. Mass-to-Charge (m/z)
NMe2
MeO
MeO
O
OEt
N
H

9b

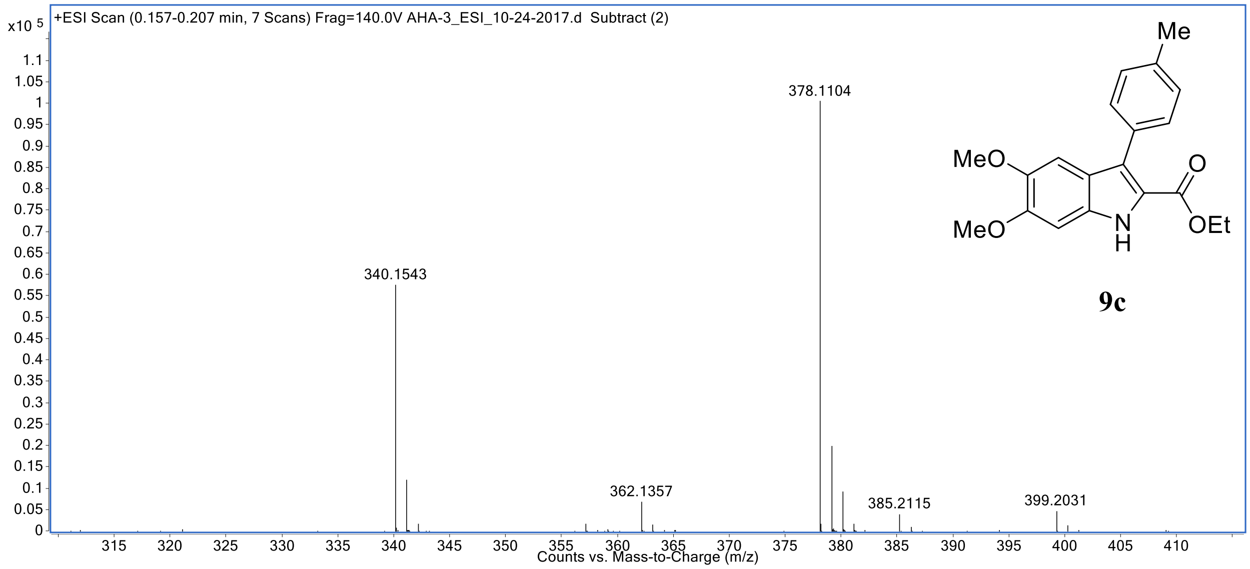
+ESI Scan (0.157-0.207 min, 7 Scans) Frag=140.0V AHA-3_ESI_10-24-2017.d Subtract (2)
x10 5
378.1104
340.1543
362.1357
385.2115
399.2031
Counts vs. Mass-to-Charge (m/z)
Me
MeO
MeO
O
OEt
N
H

9c

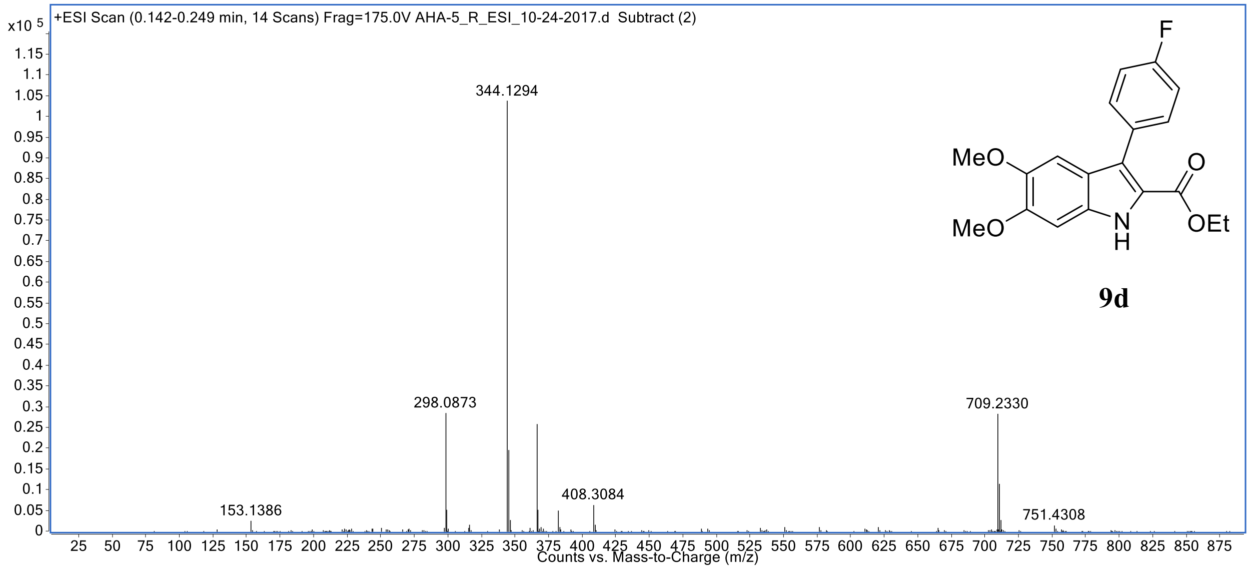

+ESI Scan (0.142-0.249 min, 14 Scans) Frag=175.0V AHA-5_R_ESI_10-24-2017.d Subtract (2)
x10 5
344.1294
298.0873
709.2330
408.3084
153.1386
751.4308
Counts vs. Mass-to-Charge (m/z)
F
MeO
MeO
O
OEt
N
H

9d

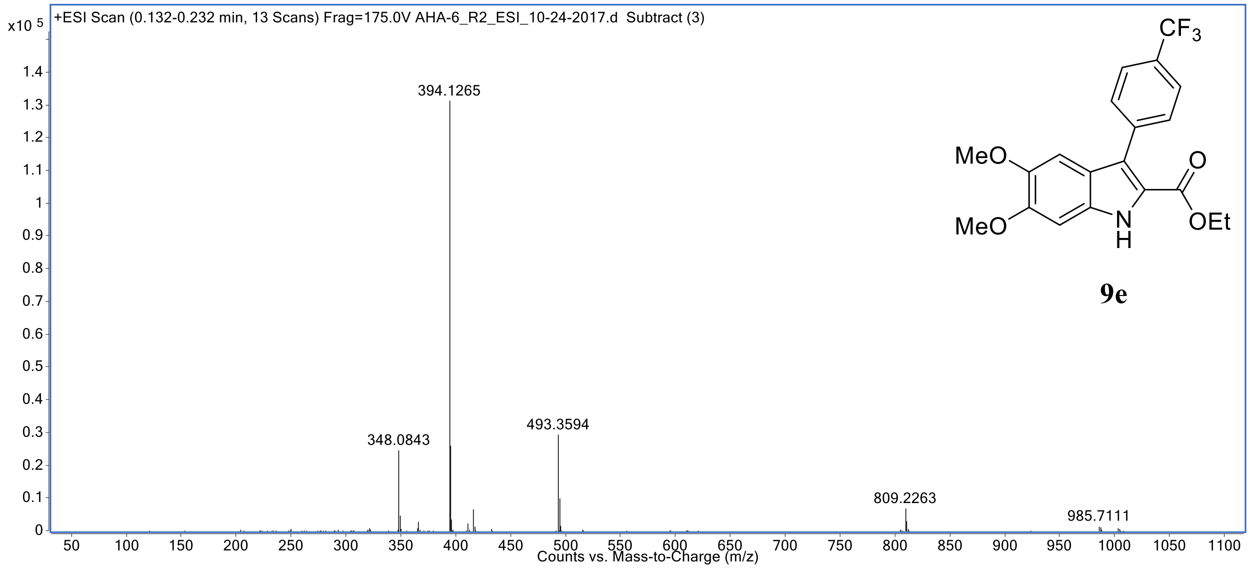

+ESI Scan (0.132-0.232 min, 13 Scans) Frag=175.0V AHA-6_R2_ESI_10-24-2017.d Subtract (3)
x10 5
394.1265
348.0843
493.3594
809.2263
985.7111
Counts vs. Mass-to-Charge (m/z)
CF3
MeO
MeO
O
OEt
N
H

9e

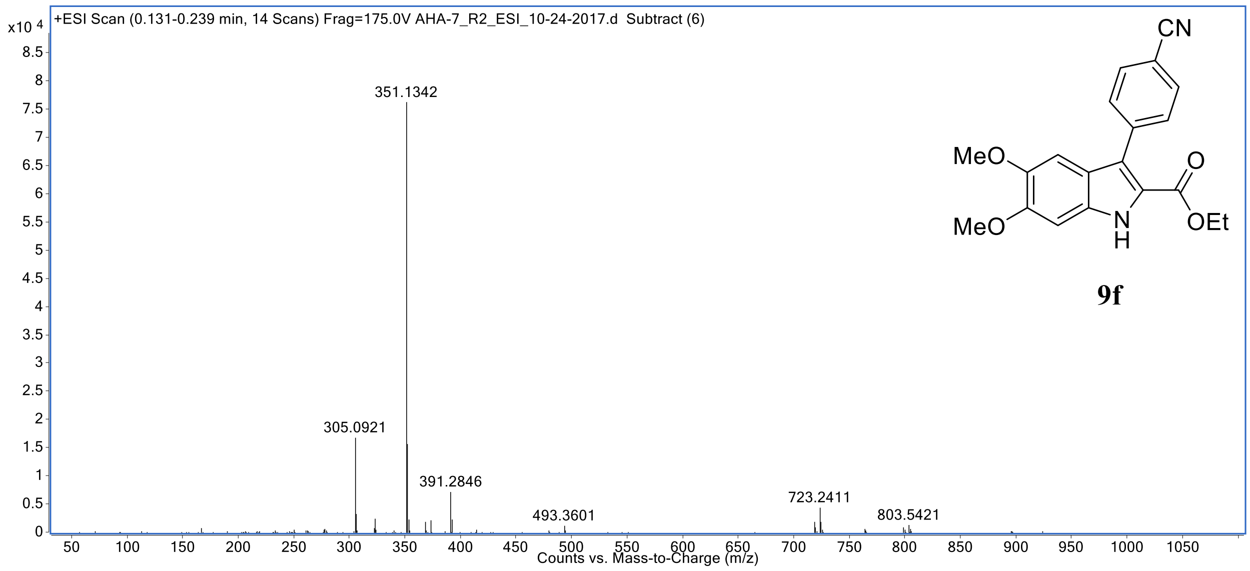
+ESI Scan (0.131-0.239 min, 14 Scans) Frag=175.0V AHA-7_R2_ESI_10-24-2017.d Subtract (6)
x10 4
351.1342
305.0921
391.2846
493.3601
723.2411
803.5421
Counts vs. Mass-to-Charge (m/z)
CN
MeO
MeO
N
H
O
OEt
9f

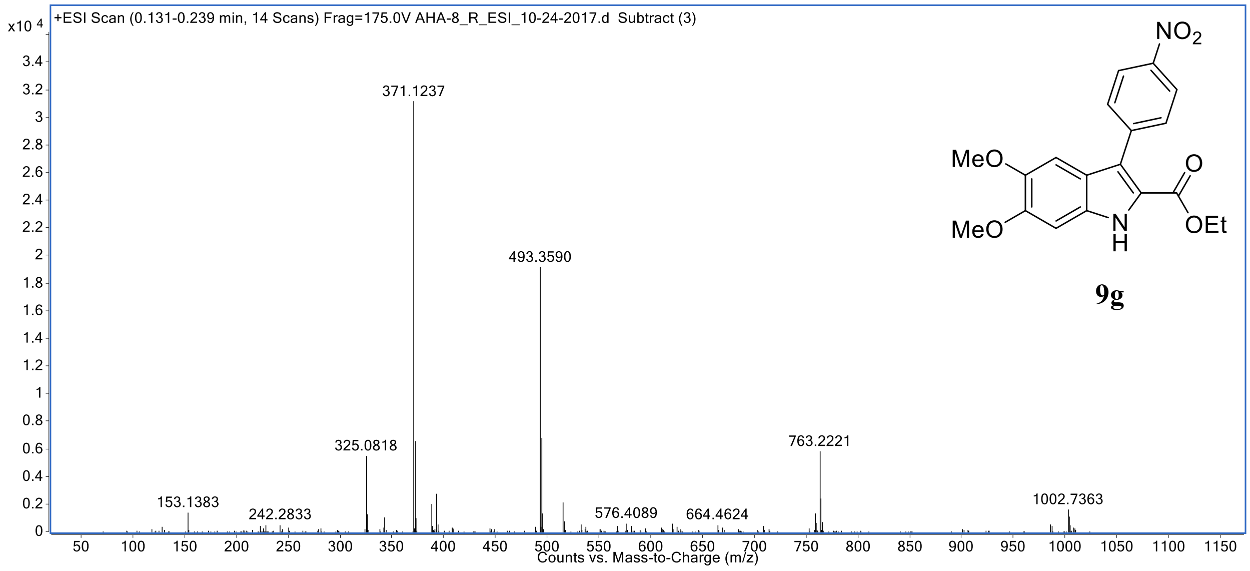
+ESI Scan (0.131-0.239 min, 14 Scans) Frag=175.0V AHA-8_R_ESI_10-24-2017.d Subtract (3)
x10 4
371.1237
493.3590
325.0818
153.1383
242.2833
576.4089
664.4624
763.2221
1002.7363
Counts vs. Mass-to-Charge (m/z)
NO2
MeO
MeO
N
H
O
OEt
9g

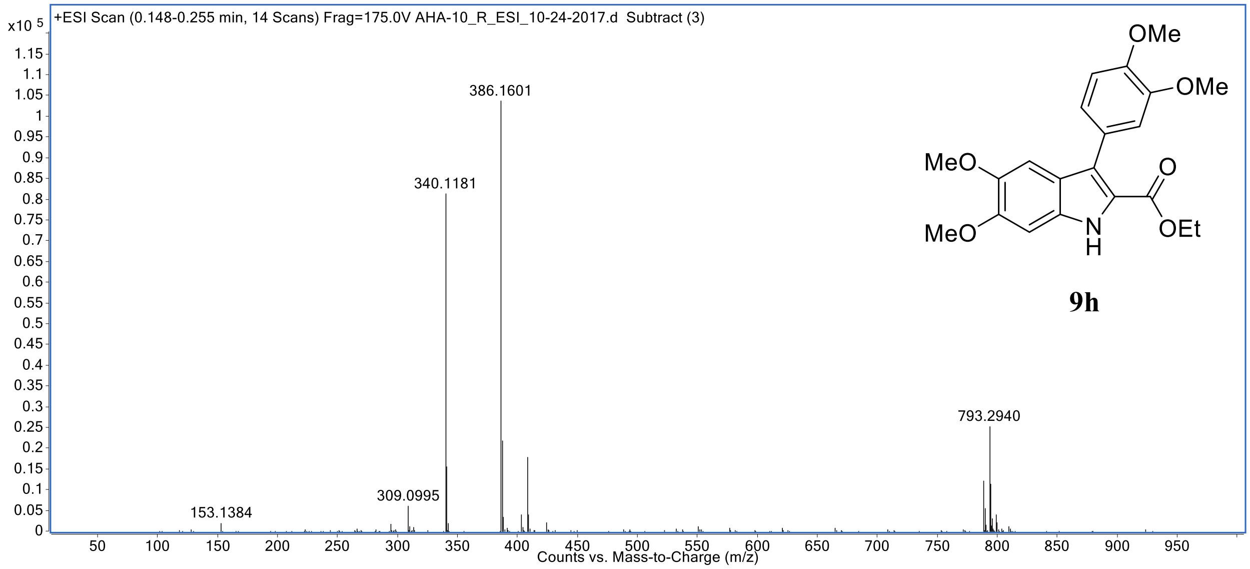
+ESI Scan (0.148-0.255 min, 14 Scans) Frag=175.0V AHA-10_R_ESI_10-24-2017.d Subtract (3)
x10 5
386.1601
340.1181
793.2940
309.0995
153.1384
Counts vs. Mass-to-Charge (m/z)
OMe
OMe
MeO
MeO
O
OEt
N
H

9h

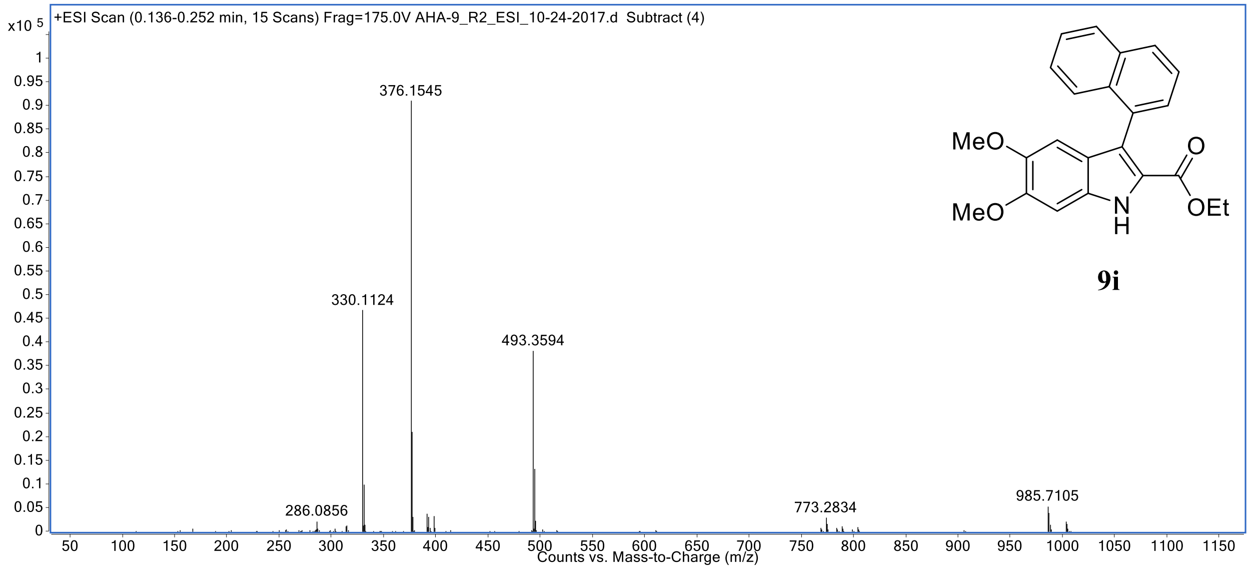
+ESI Scan (0.136-0.252 min, 15 Scans) Frag=175.0V AHA-9_R2_ESI_10-24-2017.d Subtract (4)
x10 5
376.1545
330.1124
493.3594
286.0856
773.2834
985.7105
Counts vs. Mass-to-Charge (m/z)
MeO
MeO
O
OEt
N
H

9i

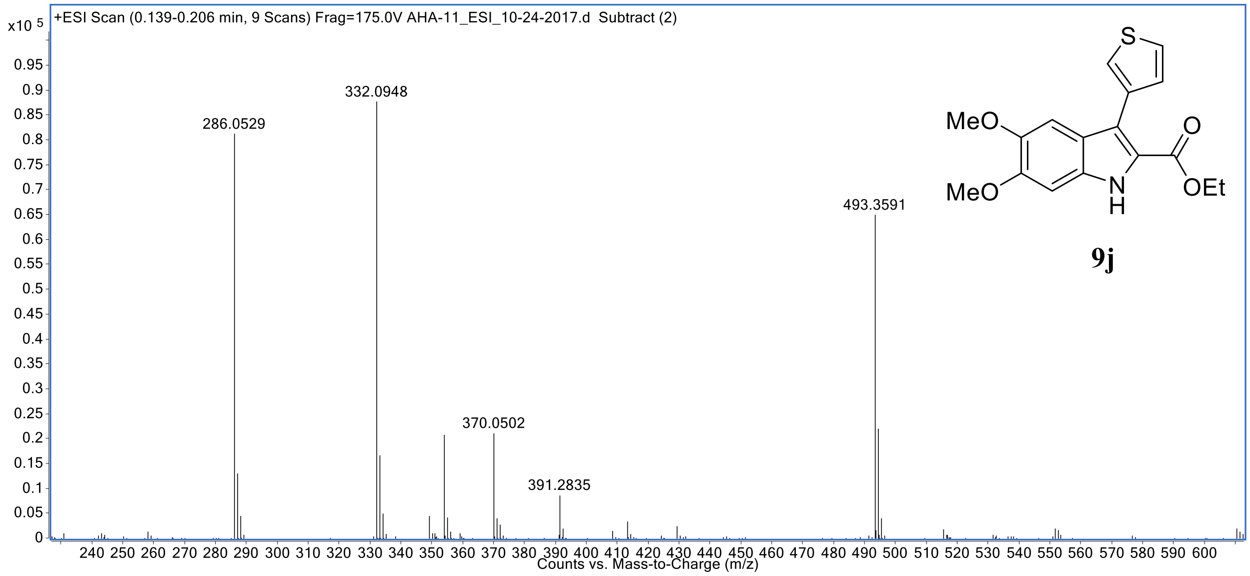
+ESI Scan (0.139-0.206 min, 9 Scans) Frag=175.0V AHA-11_ESI_10-24-2017.d Subtract (2)
x10 5
286.0529
332.0948
370.0502
391.2835
493.3591
MeO
MeO
S
O
OEt
N
H
9j
Counts vs. Mass-to-Charge (m/z)

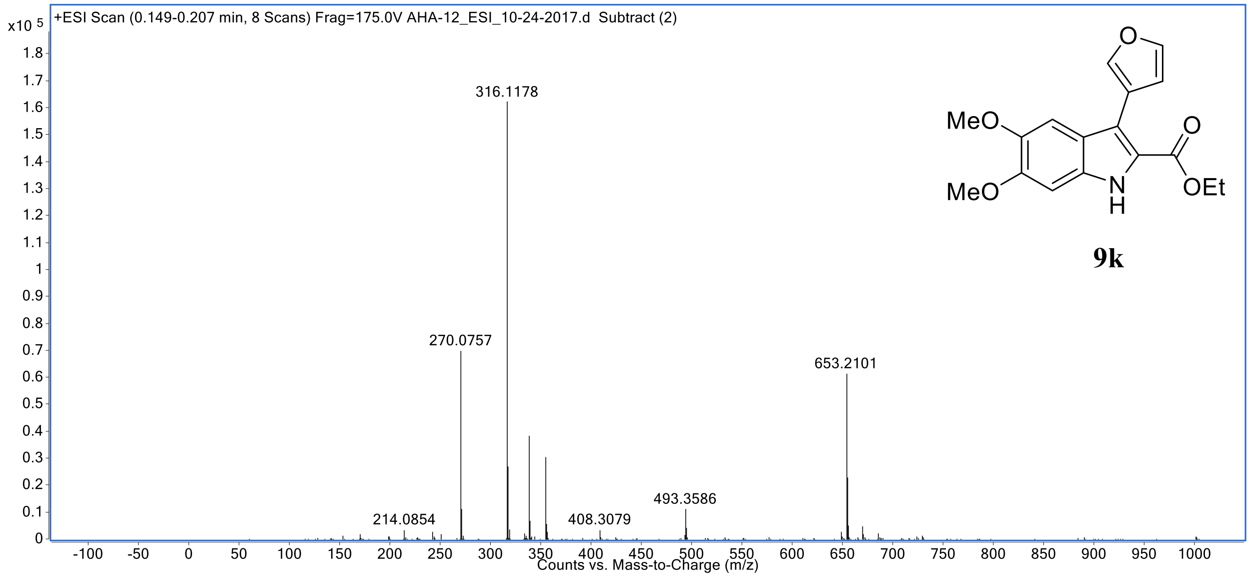
+ESI Scan (0.149-0.207 min, 8 Scans) Frag=175.0V AHA-12_ESI_10-24-2017.d Subtract (2)
x10 5
214.0854
270.0757
316.1178
408.3079
493.3586
653.2101
MeO
MeO
O
O
OEt
N
H
9k
Counts vs. Mass-to-Charge (m/z)

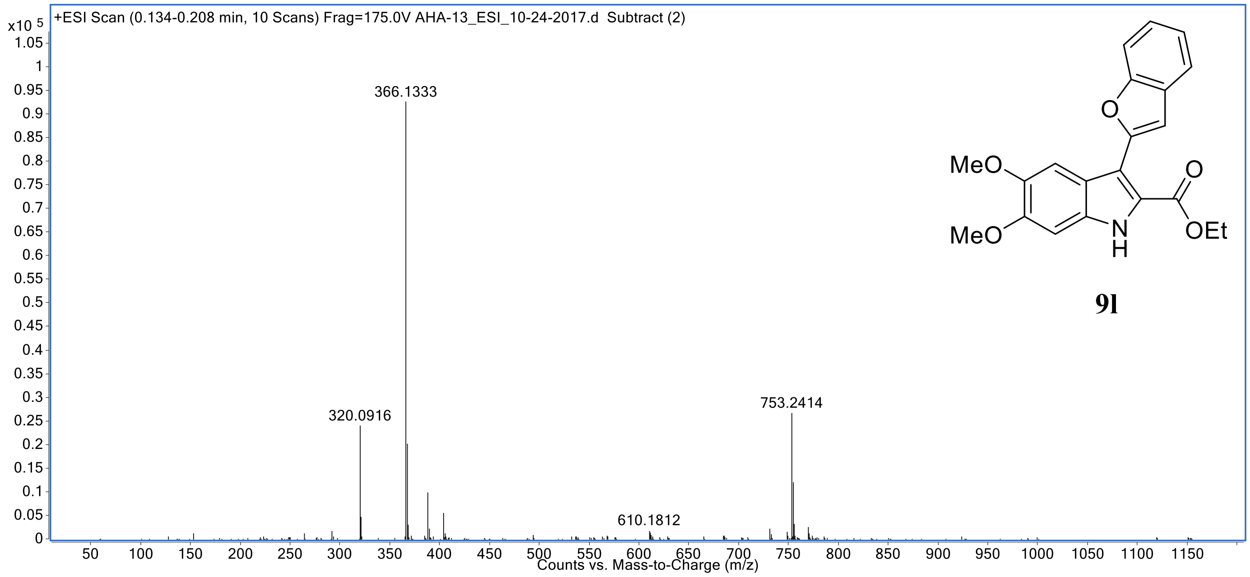
+ESI Scan (0.134-0.208 min, 10 Scans) Frag=175.0V AHA-13_ESI_10-24-2017.d Subtract (2)
x10 5
366.1333
320.0916
753.2414
610.1812
Counts vs. Mass-to-Charge (m/z)
MeO
MeO
O
N
H
O
OEt
9l

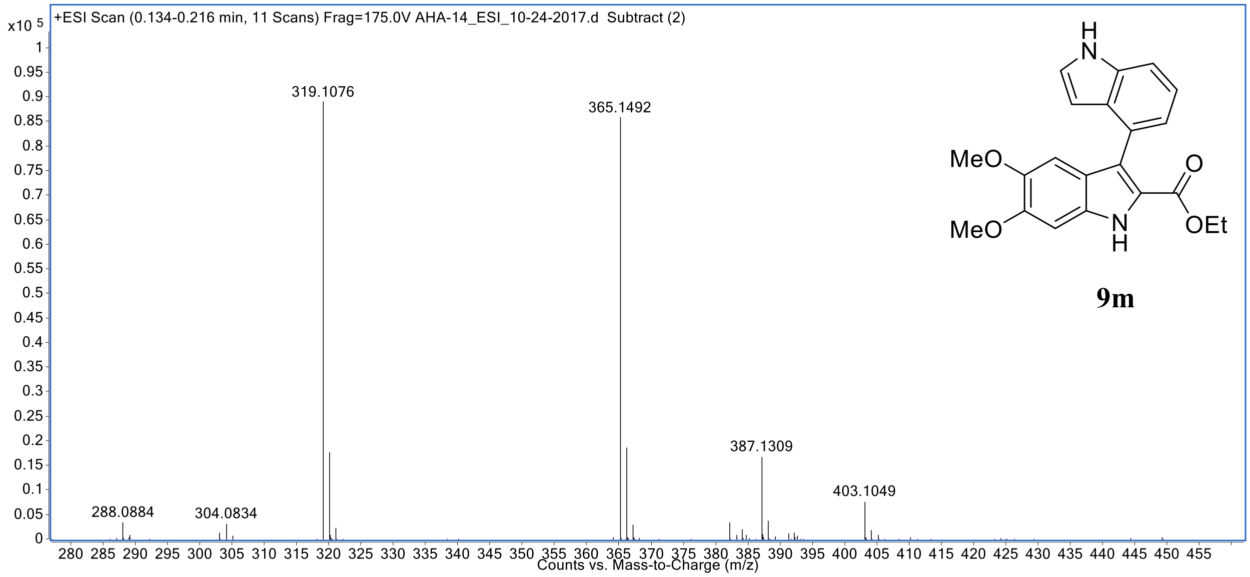
+ESI Scan (0.134-0.216 min, 11 Scans) Frag=175.0V AHA-14_ESI_10-24-2017.d Subtract (2)
x10 5
319.1076
365.1492
387.1309
403.1049
288.0884
304.0834
Counts vs. Mass-to-Charge (m/z)
H
N
MeO
MeO
N
H
O
OEt
9m

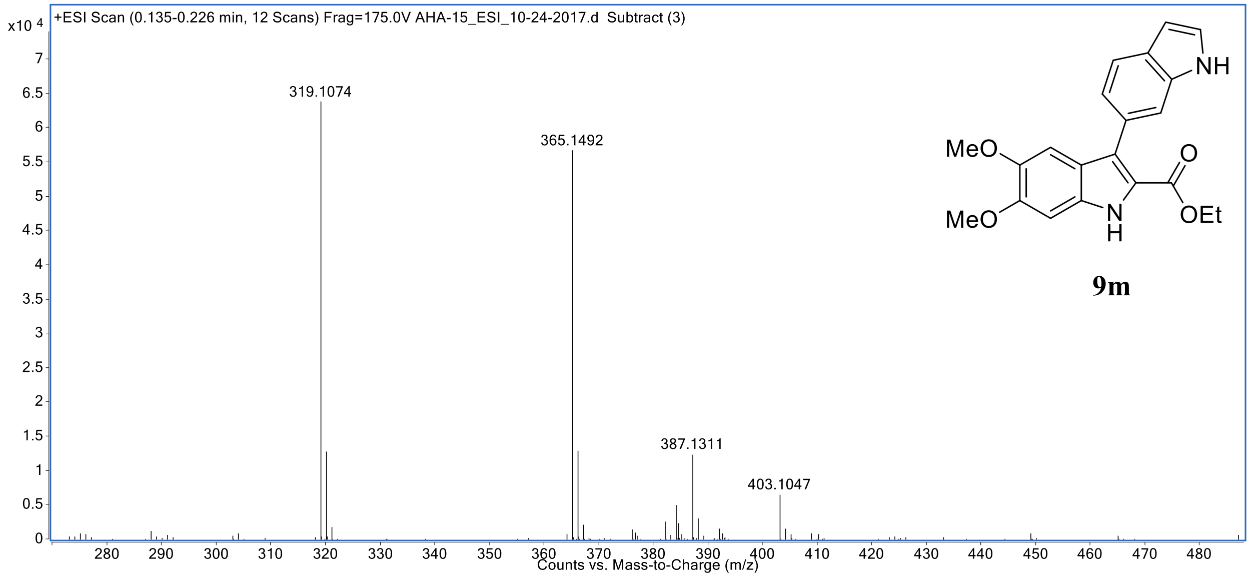
+ESI Scan (0.135-0.226 min, 12 Scans) Frag=175.0V AHA-15_ESI_10-24-2017.d Subtract (3)
x10 4
319.1074
365.1492
387.1311
403.1047
Counts vs. Mass-to-Charge (m/z)
MeO
MeO
NH
N
H
O
OEt
9m

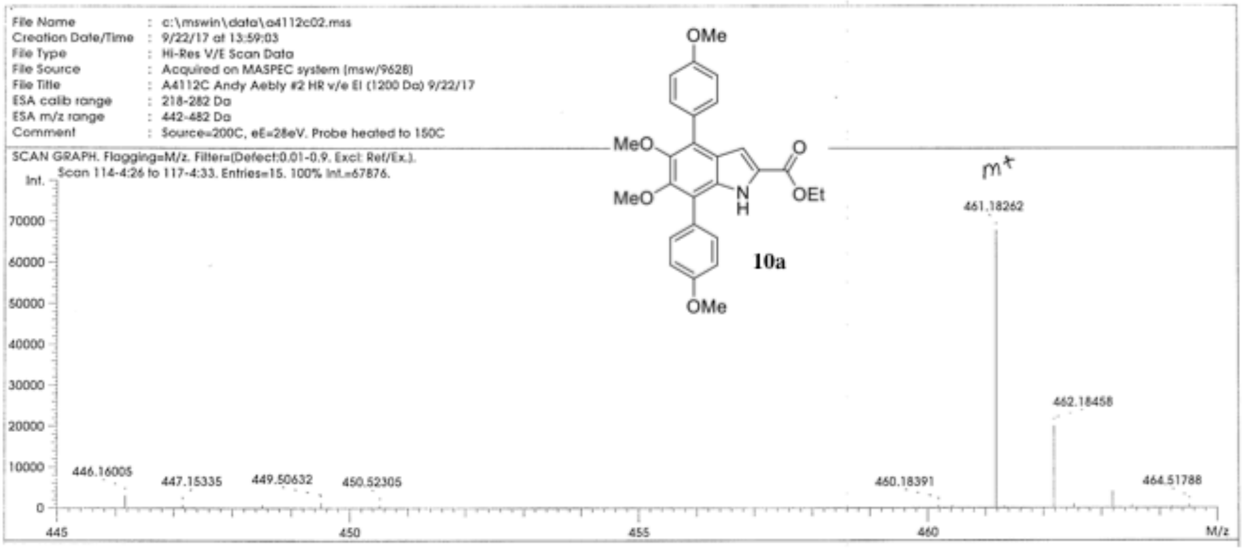
File Name : c:\mswin\data\a4112c02.mss
Creation Date/Time : 9/22/17 at 13:59:03
File Type : Hi-Res V/E Scan Data
File Source : Acquired on MASPEC system (msw/9628)
File Title : A4112C Andy Aebly #2 HR v/e EI (1200 Da) 9/22/17
ESA calib range : 218-282 Da
ESA m/z range : 442-482 Da
Comment : Source=200C, eE=28eV. Probe heated to 150C
SCAN GRAPH. Flagging=M/z. Filter=(Defect;0.01-0.9. Excl: Ref/Ex.).
Scan 114-4:26 to 117-4:33. Entries=15. 100% Int.=67876.
Int.
OMe
MeO
MeO
N
H
O
OEt
OMe
10a
m+
461.18262
462.18458
446.16005
447.15335
449.50632
450.52305
460.18391
464.51788
M/z

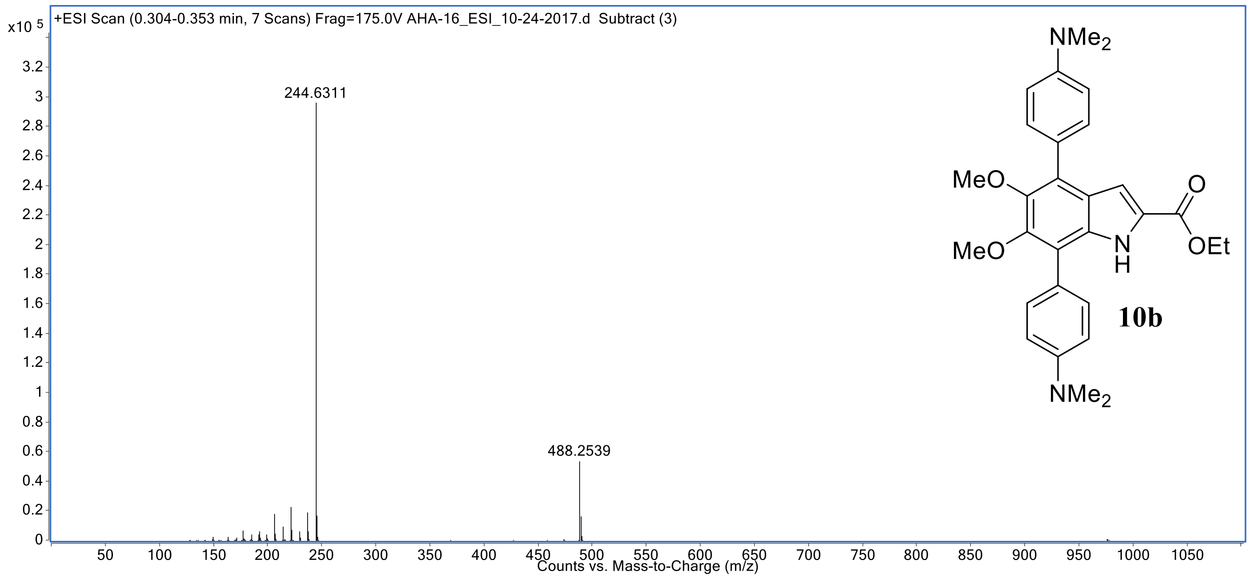
+ESI Scan (0.304-0.353 min, 7 Scans) Frag=175.0V AHA-16_ESI_10-24-2017.d Subtract (3)
x10 5
244.6311
488.2539
Counts vs. Mass-to-Charge (m/z)
NMe2
MeO
MeO
O
OEt
N
H
10b
NMe2

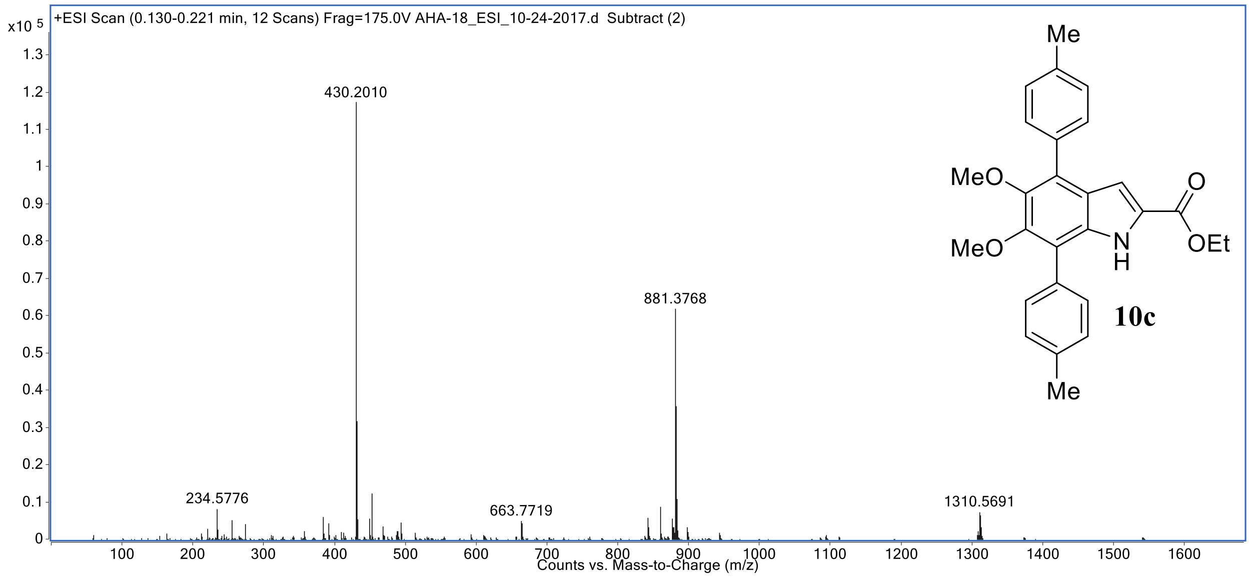
+ESI Scan (0.130-0.221 min, 12 Scans) Frag=175.0V AHA-18_ESI_10-24-2017.d Subtract (2)
x10 5
430.2010
881.3768
234.5776
663.7719
1310.5691
Counts vs. Mass-to-Charge (m/z)
Me
MeO
MeO
O
OEt
N
H
10c
Me

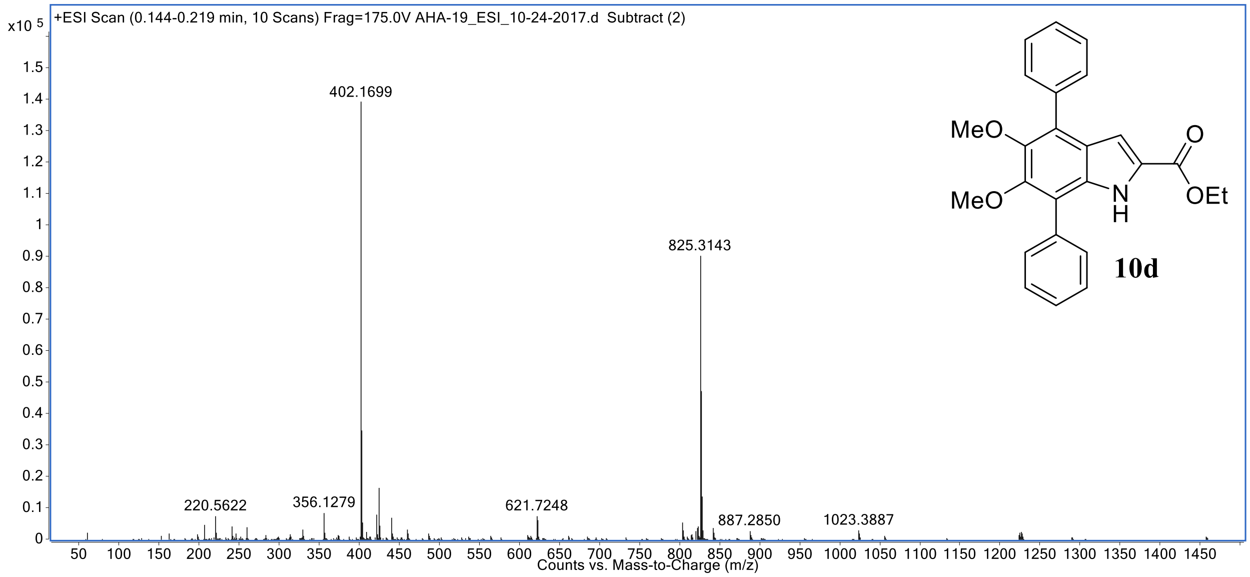
+ESI Scan (0.144-0.219 min, 10 Scans) Frag=175.0V AHA-19_ESI_10-24-2017.d Subtract (2)
x10 5
402.1699
825.3143
220.5622
356.1279
621.7248
887.2850
1023.3887
Counts vs. Mass-to-Charge (m/z)
MeO
MeO
N
H
O
OEt
10d

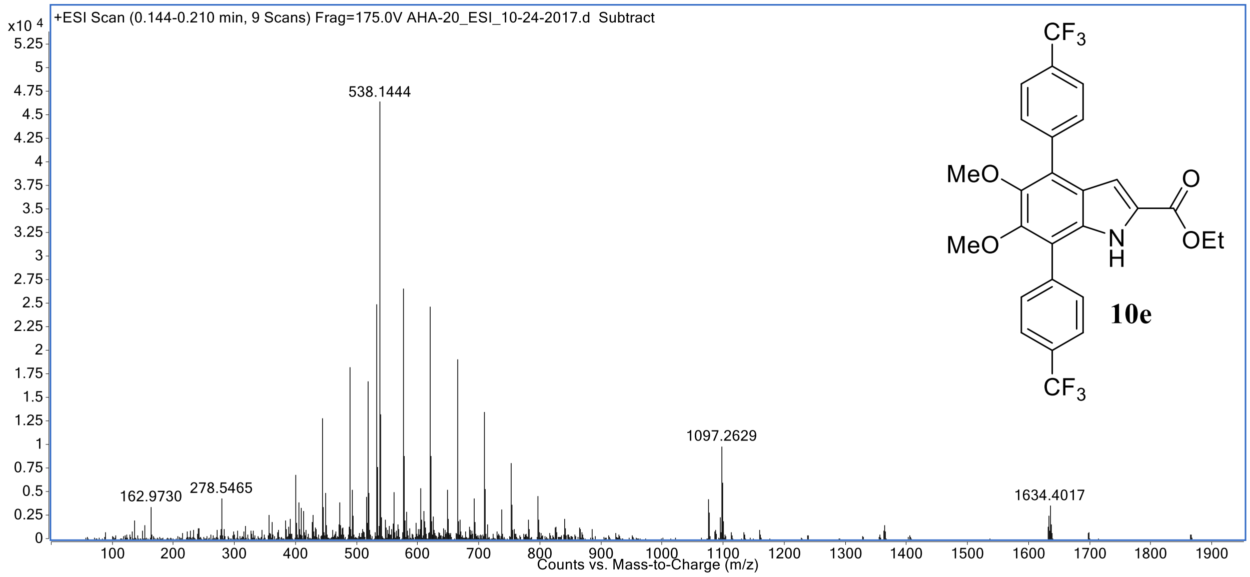
+ESI Scan (0.144-0.210 min, 9 Scans) Frag=175.0V AHA-20_ESI_10-24-2017.d Subtract
x10 4
538.1444
1097.2629
162.9730
278.5465
1634.4017
Counts vs. Mass-to-Charge (m/z)
CF3
MeO
MeO
N
H
O
OEt
CF3
10e

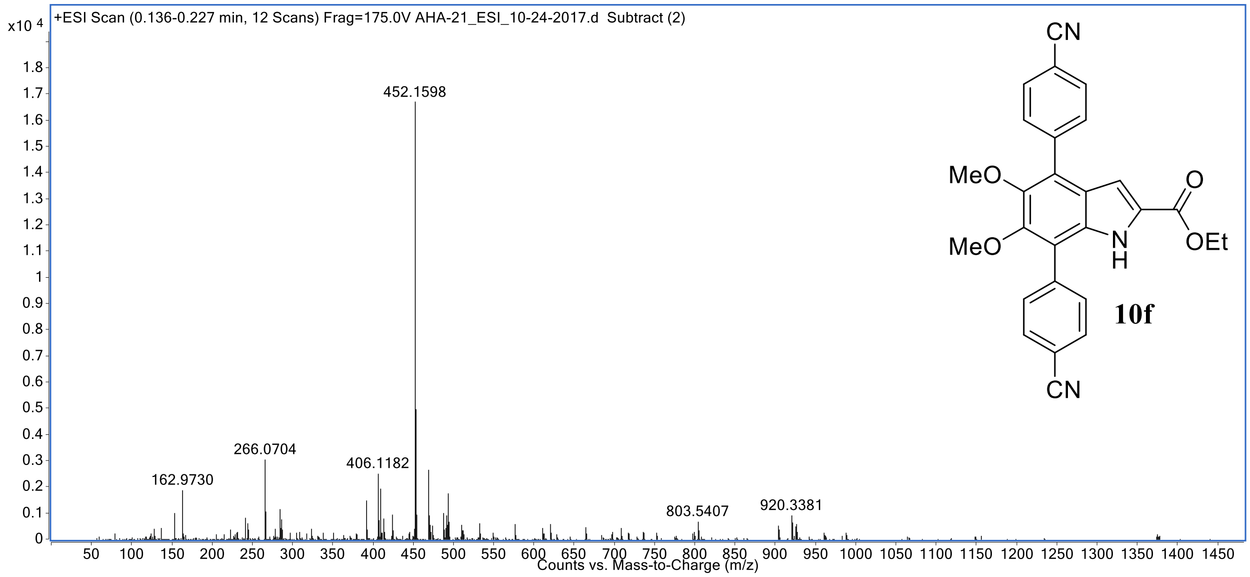
+ESI Scan (0.136-0.227 min, 12 Scans) Frag=175.0V AHA-21_ESI_10-24-2017.d Subtract (2)
x10 4
452.1598
266.0704
406.1182
162.9730
803.5407
920.3381
Counts vs. Mass-to-Charge (m/z)
CN
MeO
MeO
O
OEt
N
H
10f

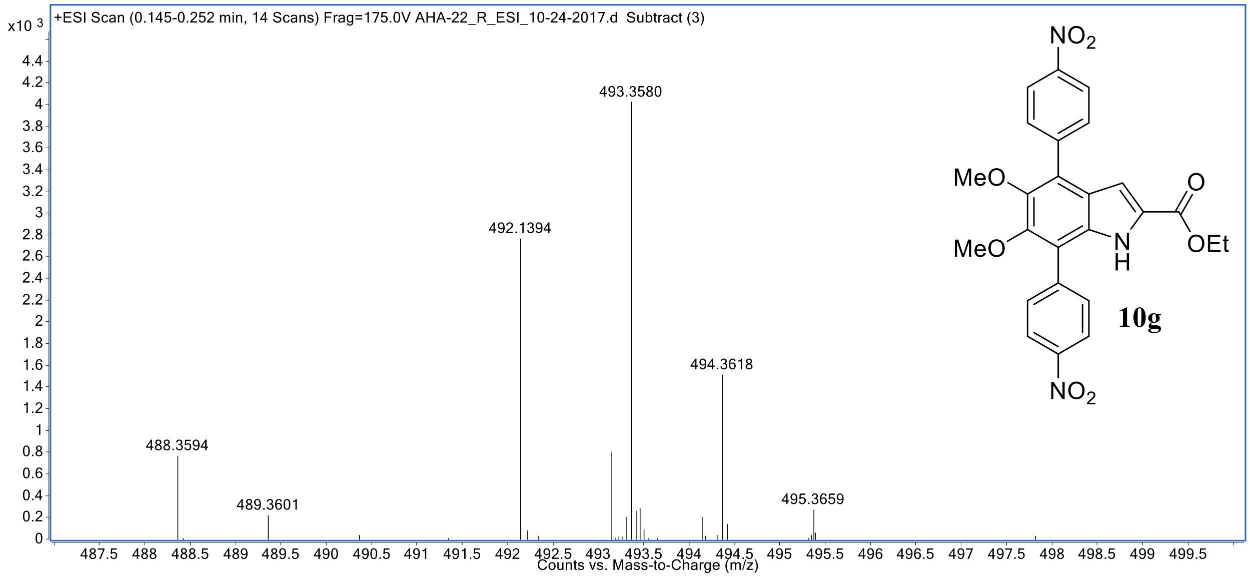
+ESI Scan (0.145-0.252 min, 14 Scans) Frag=175.0V AHA-22_R_ESI_10-24-2017.d Subtract (3)
x10 3
493.3580
492.1394
494.3618
488.3594
489.3601
495.3659
Counts vs. Mass-to-Charge (m/z)
NO2
MeO
MeO
O
OEt
N
H
10g

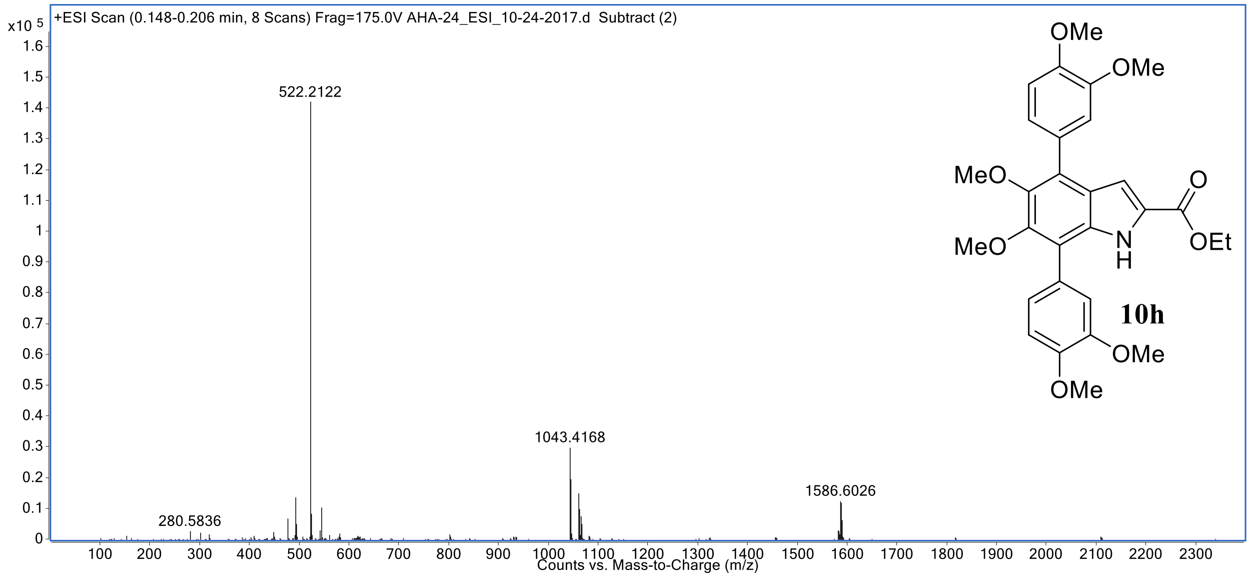
+ESI Scan (0.148-0.206 min, 8 Scans) Frag=175.0V AHA-24_ESI_10-24-2017.d Subtract (2)
x10 5
522.2122
1043.4168
1586.6026
280.5836
Counts vs. Mass-to-Charge (m/z)
OMe
OMe
MeO
MeO
O
OEt
N
H
10h
OMe
OMe

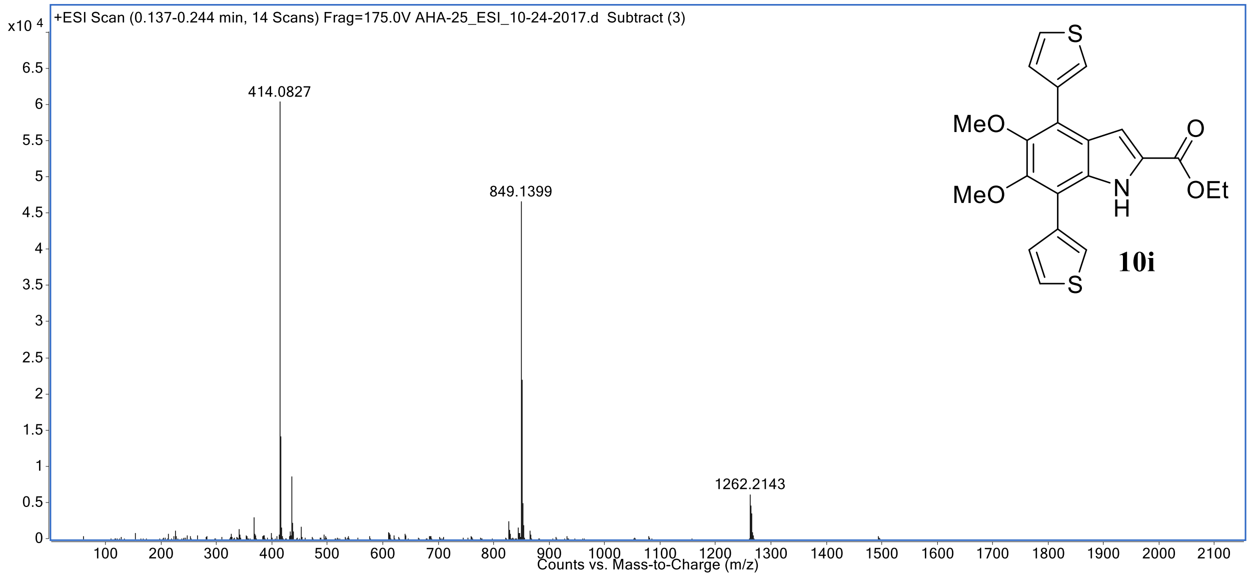
+ESI Scan (0.137-0.244 min, 14 Scans) Frag=175.0V AHA-25_ESI_10-24-2017.d Subtract (3)
x10 4
414.0827
849.1399
1262.2143
Counts vs. Mass-to-Charge (m/z)
S
MeO
MeO
O
OEt
N
H
10i
S

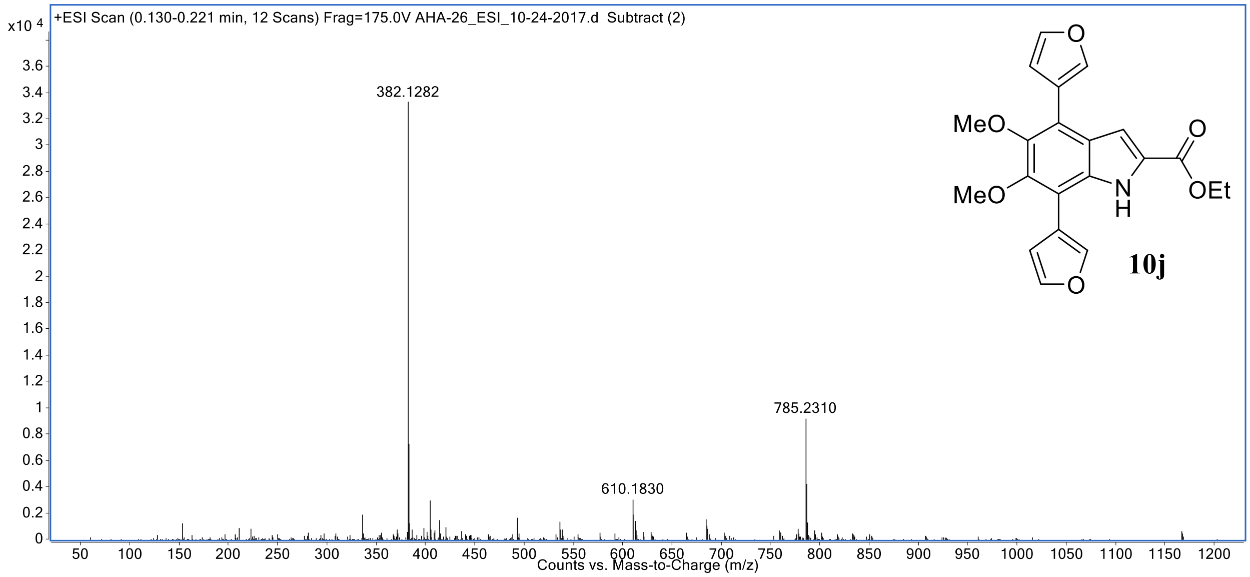
+ESI Scan (0.130-0.221 min, 12 Scans) Frag=175.0V AHA-26_ESI_10-24-2017.d Subtract (2)
x10 4
382.1282
785.2310
610.1830
Counts vs. Mass-to-Charge (m/z)
MeO
MeO
O
OEt
N
H
10j

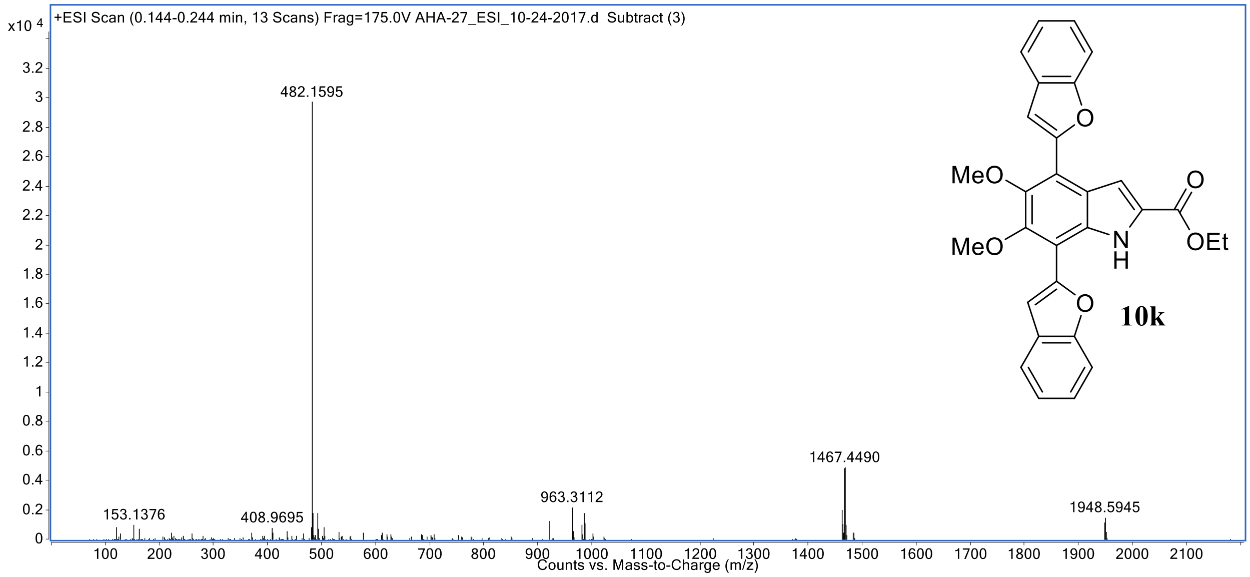
+ESI Scan (0.144-0.244 min, 13 Scans) Frag=175.0V AHA-27_ESI_10-24-2017.d Subtract (3)
x10 4
482.1595
1467.4490
963.3112
1948.5945
153.1376
408.9695
Counts vs. Mass-to-Charge (m/z)
MeO
MeO
O
OEt
N
H
10k

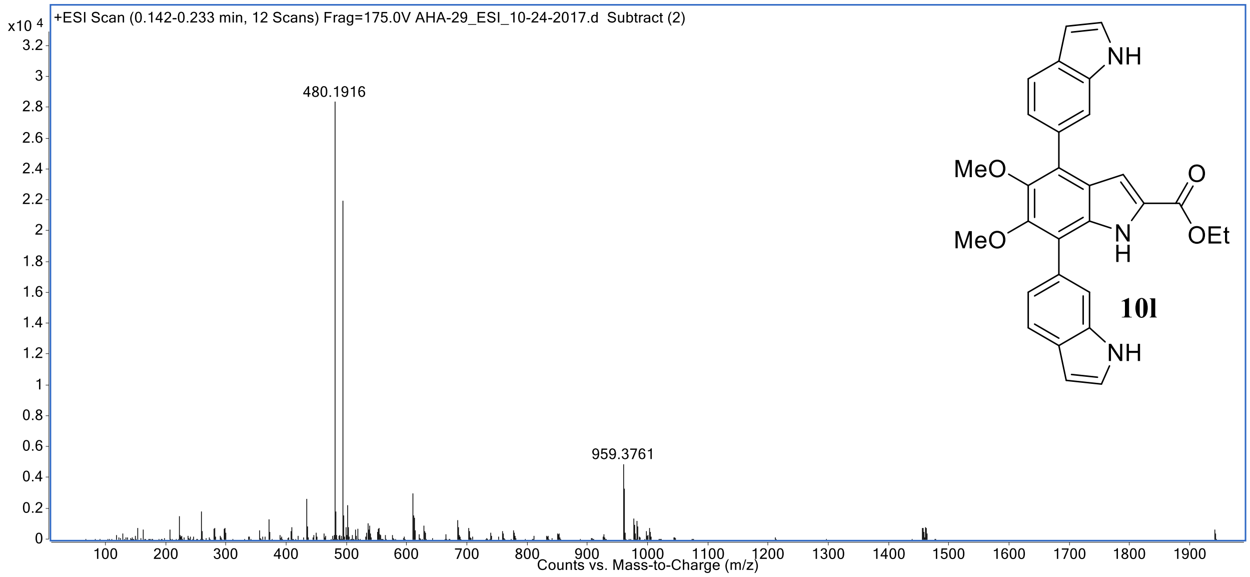
+ESI Scan (0.142-0.233 min, 12 Scans) Frag=175.0V AHA-29_ESI_10-24-2017.d Subtract (2)
x10 4
480.1916
959.3761
Counts vs. Mass-to-Charge (m/z)
MeO
MeO
NH
NH
N
H
O
OEt
10l
